# 'Not all that burns is wood'. A social perspective on fuel exploitation and use during the Indus urban period (2600-1900 BC)

**DOI:** 10.1371/journal.pone.0192364

**Published:** 2018-03-07

**Authors:** Carla Lancelotti

**Affiliations:** CaSEs Research Group, Department of Humanities, Universitat Pompeu Fabra, Barcelona, Spain; New York State Museum, UNITED STATES

## Abstract

Ancient civilisations depended heavily on natural fuel resources for a wide array of activities, and this had an impact on such resources that can be traced in the archaeological record. At its urban apex, the populations of the Indus Civilisation (2600–1900 BC) produced a wide range of objects and crafts, several of which involved highly specialised pyrotechnology. In the wake of increasing aridity and a period of weakened monsoon rainfall that affected South Asia from 2100 BC, these activities potentially put pressure on the natural resource base that may have had to be counterbalanced by differentiation in fuel use. The combined analysis of archaeobotanical and geoarchaeological remains from four Indus urban phase archaeological sites, has enable an assessment of the mechanisms through which people exploited wood, and diversified their fuel resources to adapt to the arid to semi-arid environments in which they lived. The combined use of local wood species with alternative fuels, such as dung and crop-processing leftovers, are evidence for resilient socio-ecological practices during the 700 years of Indus urbanism and perhaps beyond.

## Introduction

The reconstruction of how people exploited and used fuel resources in the past is one of the tools for exploring human-environment interactions. How societies related to the available natural resources is not only a matter of climate and environmental conditions. There are numerous practical as well as social and cultural motives that compel people to burn specific fuels or apply a determined strategy of fuel exploitation [1]. This paper explores the socio-ecological behaviours that underlie the gathering and utilisation of fuel resources during the Indus urban period (2600–1900 BC) of the Indus Civilisation of northern South Asia. This period corresponds to an expansion phase when large urban centres and small rural settlements co-existed across a wide area that comprises modern Pakistan and north-west India and encompassed several ecological zones [2–5]. In the past, it has been suggested that some aspects of the Indus urban period material culture were highly uniform throughout the area occupied by Indus populations [5–7] although it is increasingly recognised that a certain degree of cultural variability existed [3]. The nature of this regional diversity is particularly evident in the analyses of subsistence practices with recent works stressing the role of ecological variability within the vast area occupied by the Indus Civilisation [e.g. 3, 8–9].

This paper addresses one main question: how did a complex urban society that flourished in a range of distinctive environmental zones relate to the environment in respect to fuel resources? In order to tackle this question it is paramount to adopt methods that allow for the correct identification not only of fuelwood, but also of all types of fuel that were potentially used.

### Archaeological identification of fuel alternative to wood

Ethnographic research has demonstrated that fuels that are alternatives to wood, such as dung and crop processing leftovers, are extremely important in arid areas where wood is not easily available. However, these types of fuel do not always leave macroscopic evidences in the archaeological record, as they normally tend to burn out completely into ash. This limitation highlights the need of integrating different techniques to detect the use of these alternative fuels. Whereas the use of crop-processing leftover as fuel is not usually investigated, dung identification in archaeological context has received ample attention over the last few decades and several excellent reviews have been published [10–13]. The main methods for dung identification in archaeological samples have relied on the analysis of one of the following proxies (or a combination of two or more of them): presence/absence of spherulites [14–17]; charred macro remains of small seeds assemblages [18–25]; phytoliths presence, concentration and morphology [26–29]; physic-chemical characterization of sediments [30–31]; presence of specific parasites [32–33]; identification of biomarkers such as coprostanol and bile acids [34–37]; and DNA analysis [37–38].

In order to reconstruct fuel use practices, a number of different proxies have been analysed in this study. Anthracological (i.e. wood charcoal) remains representing the direct evidence of fuelwood has been complemented by a combination of phytoliths, spherulites and chemical elements according to the methodology published by Lancelotti and Madella [10], which enables the assessment of the presence of dung and crop processing leftovers being used as fuel. This methodology has been chosen for its demonstrated high level of confidence for dung identification, as well as for the discrimination between dung and crop processing leftover used as fuel in the study area [10].

### Environment and paleoenvironment

The area covered in this study is today classified as hyper- to semi-arid according to the CGIAR-CSI Global-Aridity and Global-PET Database [39, 40]. According to historical data supplied by the Indian and Pakistan Meteorological Departments [41–42] the average yearly precipitation varies between 500 mm in North Gujarat and Western Uttar Pradesh and 371 mm in Punjab; the average annual temperature (in degrees centigrade) is 26.7 for North Gujarat, 25.2 for Western Uttar Pradesh and 23 for Punjab.

The four sites object of this study (see next paragraph) span over the entire territory occupied during the Indus urban period and, despite the fact that they are located in different areas and on different river systems, they were all situated in zones with similar vegetation (albeit with some degree of regional difference) [43]. The modern Indian states of Gujarat and Uttar Pradesh, and the Pakistani province of Punjab fall into the Palaeotropical floristic kingdom, and they are all part of the Sudano-Zambesian floristic region. According to the Global Forest Resource Assessments compiled by the FAO in 2010 [44–46] Gujarat and Uttar Pradesh are characterised by tropical scrubland and Pakistani Punjab is characterised by tropical desert.

**Fig 1:**
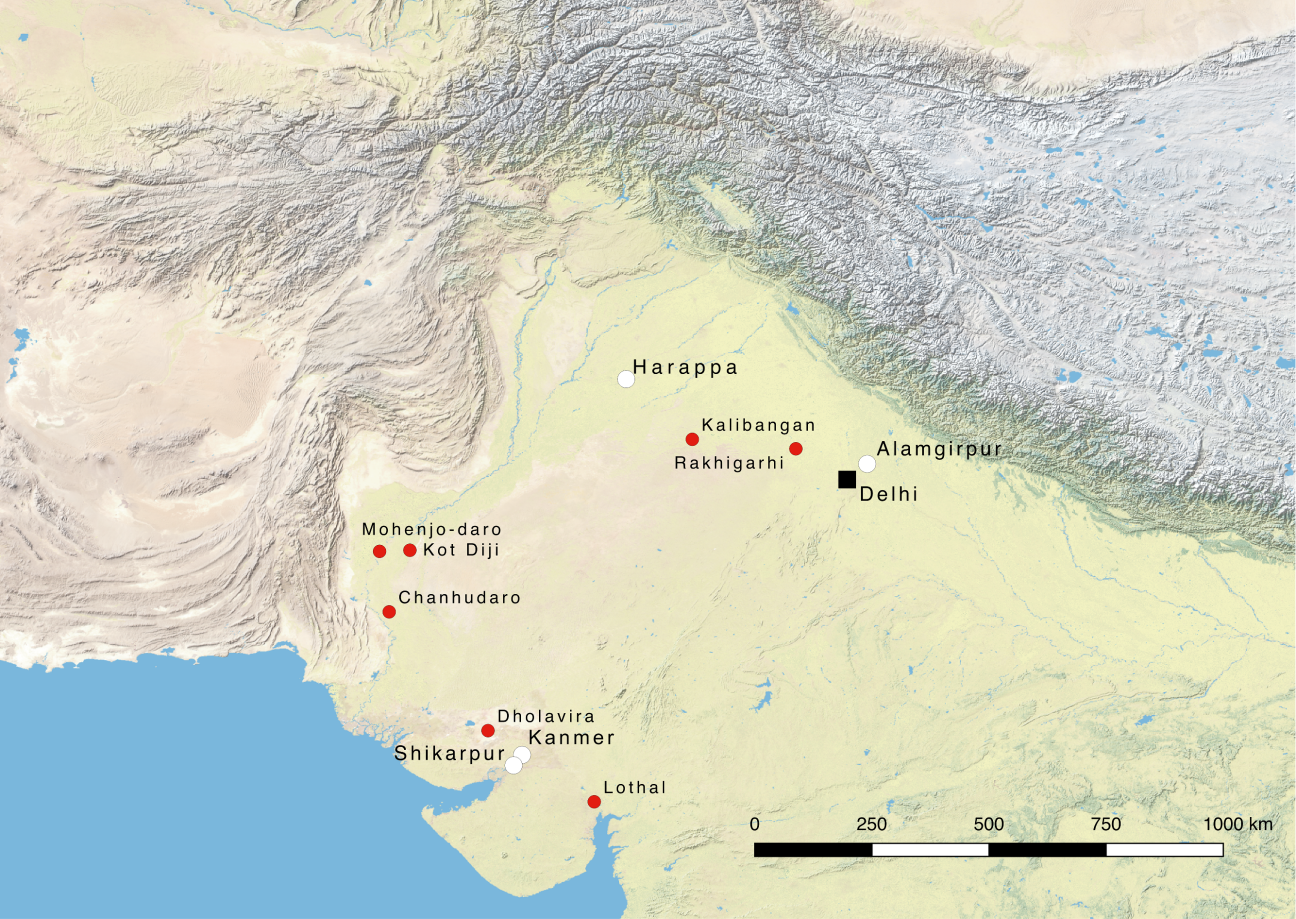
Map of northern South Asia. The map shows the location of the 4 sites discussed in this study (white) as well as some of the main archaeological sites pertaining to the Indus Civilisation.

Available palaeoclimatic data show that in the third millennium BC, this region was undergoing a period of progressive aridification marked by periods of acute monsoon weakening [47–48], and fuelwood may thus have been scarce. Alternative sources of fuel, such as dung and crop processing leftovers, may therefore needed to be exploited, which is very much in keeping with today’s practices. For the study presented here, biological and chemical proxies were combined to analyse samples from contexts directly related to fuel use (*i*.*e*. fireplaces and ashy patches), as well as material from contexts that might have been indirectly related to fuel discard (*i*.*e*. pits, floors and street deposits). These samples originated from levels dated to the Indus urban period of four different sites (Fig 1) [10, 49].

### Study-sites

The research presented here has set out to highlight similarities and differences in fuel exploitation practices across different geographical areas, and thus presents a synchronous study of a single cultural phase chronology rather than a multi-temporal analysis. This choice originates from the desire to understand human impact on the environment during the phase in which the Indus society was at its apex and the range of activities that were carried out and required the use of fuel was widest. Nonetheless, the Indus urban period lasts approximately 700 years and the samples analysed cover the entire span, thus allowing some inferences on chronological change in the fuel use. Moreover, in order to pinpoint differences related to occupation types, the sites chosen covered the entire spectrum, from a large city that is considered one of the "capitals" of the Indus Civilisation (Harappa, Punjab), to a medium-sized site with stone wall fortifications (Kanmer, Gujarat), a medium-sized site with mud-brick walls (Shikarpur, Gujarat), and a relatively small rural village (Alamgirpur, U.P.).

#### Harappa

The archaeological site of Harappa (30° 37’ 31” N, 72° 53’ 0” E) is situated in proximity of the homonymous modern town, in Sahiwal (former Montgomery) district of the Pakistani province of Punjab. The first inhabitants of Harappa settled on an elevated area of the terraced river plain [50–51] on the left bank of a channel of the Ravi River, one of the five rivers that flow into the Indus and give the name to Punjab, the land of five rivers. Studies of soil and landscape formation have shown that Harappa grew on one of the oldest soils of the area [52–53], which was probably chosen for its vicinity to the fertile alluvial plain and, at the same time, because its raised position offered some protection against flooding. The main channel of the Ravi River now flows *c*. 10 kilometres north of Harappa but appears to have meandered around the settlement location both before and after human occupation. Different sections of the settlement were occupied in different time periods, which resulted in the formation of many mounds [54].

The analysed material originated from samples collected over 6 field seasons (1988, 1989, 1990, 1993, 1997 and 2000). Most of the samples, both flotation and sediment, were collected in mound E; samples cover the entire span of Period 3 (Urban period) with the majority pertating to the central phase of this period (phase 3B). All the macro-botanical remains were recovered by flotation of c. 10 litres of sediment or less depending on the dimension of the contexts [55]. Some of the charcoal samples analysed in the present work were obtained from S. Thiébault and M. Tengberg, to whom they were initially sent for analysis. Other charcoal samples were obtained through sorting of samples stored with S. Weber at the Laboratory for Environmental Archaeology at the Washington State University in Vancouver, USA. Soil samples for phytolith analysis were collected in 2001 by M. Madella during his fieldwork at Harappa.

#### Kanmer

The archaeological mound, known locally as Bakar kot, stands to the north of the modern village of Kanmer (23°23’N; 70°52’E), located in Rapar Taluka, Kachchh District. The archaeological mound measures 105x115 m and rises 8 m above the surrounding plain [56–57]. It is encircled by a massive stonewall and its top is shallow with peripheral regions slightly higher than the central part. An ephemeral stream (*nullah*), known as Aludawaro Vokro that originates in an inselberg-type hill 2 km west of the mound, drains the area around the site. Rajaguru and Shuhsama [58] suggested that the *nullah* was permanently active during the Indus period. Presently, the only source of water, except deep ground water, is a large natural tank located to the south east of the hill. It is not as yet clear whether this water basin was in use during the Indus period.

Samples analysed in this work were collected by the author during the 2007–2008 field season. During this field season three areas were investigated through 5x5 trenches: two, in the south eastern corner and in the centre of the mound, to clarify the cultural and stratigraphic sequence; and the third, in the north west corner was excavated to understand and elucidate the fortification wall’s construction phases. A small number of domestic fireplaces, and one bigger furnace, dated to the Indus urban period were discovered during excavation.

#### Shikarpur

The archaeological mound, locally known as Valamiyo Timbo is located c. 4.5 km south of the modern village of Shikarpur along the margin of a narrow creek that runs eastwards towards the Rann of Kutch (23° 14’ 15” N, 70° 40’ 39” E). The site is approximately 3.4 hectares in area and occupies almost the entire top of a stabilised sand dune that rises 7.5 to 8 m from the surrounding plain [59]. Two water gullies, one deeper roughly oriented East-West and one shallower oriented North-South, cut the mound into three ridges. Three cultural phases were identified at the site on the basis of cultural material: Phase I attributed to the Indus Urban period; Phase II characterized by an increase in local pottery; and Phase III ascribed to the Indus post-urban phase.

The material analysed for this study were collected by the author during the 2008 field-season from 5 trenches and from layers culturally dated to the Indus urban period.

#### Alamgirpur

The archaeological site of Alamgirpur is located in District Meerut in the state of Uttar Pradesh. The site is located 27 km west of Meerut and 45 km northeast of New Delhi (29° 00.206’; 77° 29.057’. It is situated in the plain comprised between the rivers Ganga and Yamuna (the Ganga-Yamuna doab), presently ca. 3 km to the east of the Hindon River, a tributary of the Yamuna [60]. The archaeological mound, known locally as Parasuram-ka-khera, located on the east of the modern town, sits on a consolidated sand dune that raises ca. 1.5 m from the surrounding flood plain. The mound measures approximately 60 m east-west by 50 m north-south [60–61] and the top is about 6 m above the surrounding plain. Cultivated fields of sugar cane and wheat surround the site, hence the natural landscape has been heavily modified by modern anthropic activities. In 2008 the site was re-excavated by the UKIERI project “Land, Water and Settlements” [60]. The three trenches excavated on the top of the mound, ZB-1, ZB-2 and YD-2 presented deposits that dated from the Kushana period (1st-3rd century AD) to the Early Harappan (3300–2600 BC). On the southern side of the mound, erosion and recent undercutting have created a vertical section of about 5 m that was systematically sampled for archaeobotanical studies. The entire sequence in this area belongs to the Indus urban period with the uppermost layers covering the first part of the transition to post-urban. All samples analysed in this study were collected within this sequence by the author.

## Material and methods

Analyses of charcoal, phytoliths and spherulites were conducted at the George Pitt-Rivers Laboratory for Bioarchaeology, University of Cambridge. All samples were collected and exported with permission from the Archaeological Survey of India (ASI–for Kanmer, Shikarpur and Alamgirpur) and the Department of Archaeology, Government of Pakistan (Harappa). The details of the archaeological samples that have been analysed are presented in Table 1. All protocols and raw data used for this study are available as supplementary material.

**Table 1:** Summary of the samples analysed, indicating number of samples of each context and proxy at the four sites, and the main quantitative data. *density at Harappa is inferred from indirect data on quantity of soil floated derived from Steve Weber’s work on macroremains [62–63].

		Samples (n)			Charred remains (excl. seeds/grains)								Phytoliths			Spherulites
Site	Context		Flotation	Sediment	vol (ml)	Weight (g)	frag.N	Diversity	density (g/l)	% undet	r2 den/div	r2 div/und	conc (k)	Morpho-types (n)	r2	pres/ n. samples
**Harappa**	**fireplaces**		21	4	4.36	1.11	1370	0.53	0.123*	2.83	—	0.40	654	9.20	0.60	2/4
	**pits**		2	—	0.30	0.12	179	0.74	0.013*	13.19			—	—		—
	**floors**		3	2	1.86	0.47	473	0.70	0.052*	6.57			2948	11.50		0/2
	**ash**		—	3	—	—	—	—	—	—			780	16.30		0/3
	**street**		—	7	—	—	—	—	—	—			153	8.40		1/7
**Kanmer**	**fireplaces**		4	8	1.10	0.16	307	0.75	0.008	26.95	0.83	-0.45	11371	15.60	0.34	7/8
	**floors**		1	2	6.00	2.29	136	0.83	—	—			7027	12.50		0/2
	**ash**		2	4	3.90	1.16	248	0.85	0.015	15.77			16448	14.30		1/4
	**plaster**		—	2	—	—	—	—	—	—			8118	13.00		0/2
	**occupation**		3		5.55	1.83	531	0.83	0.038	25.89			—	—		—
**Shikarpur**	**fireplaces**		5	3	0.22	0.20	329	0.51	0.018	23.84	-0.26	0.43	18329	16.50	0.51	0/3
	**pits**		2	2	<1	0.12	86	0.44	0.006	12.69			15050	13.50		1/2
	**floors**		3	4	<1	0.14	150	0.68	0.007	17.19			6738	11.00		0/4
	**ash**		—	4	—	—	—	—	—	—			15872	13.75		1/4
	**plaster**		—	3	—	—	—	—	—	—			2975	9.00		0/3
	**occupation**		5	—	<1	0.08	197	0.72	0.006	23.62			—	—		—
**Alamgirpur**	**pits**		5	6	0.28	0.06	142	0.24	0.002	12.27	0.73	0.03	27950	17.20	-0.12	0/6
	**floors**		7	7	0.25	0.05	195	0.33	0.003	23.50			21903	16.40		0/7
	**ash**		3	3	0.30	0.03	49	0.07	0.002	34.07			31182	16.30		1/3

### Anthracological remains

Charcoal was collected by means of bucket flotation with a 500 μm mesh. A standard measure of 20 L of sediment was floated except for small contexts, in which case 10 L were floated. Charred remains were separated under a low-resolution microscope, and weight and volume of charcoal were recorded. Charcoal was then sieved through a 2 and 1 mm sieve column to separate fractions and facilitate analysis. Observation of fractions >1mm was carried out under a Leica DMRM, incident light microscope at 100x, 200x and 400x magnification. When possible a minimum of 150 fragments per contexts was analysed; however, preservation of charred remains in highly alkaline sediments is problematic and often the total number of remains was fewer than 150 fragments. Identification was performed by means of comparison to published material [e.g. 64–65] and to a reference collection of both fresh wood thin sections and charred wood from species indigenous to the study area. The reference collection was created by the author [49] using wood collected in the field as well as specimens provided by M. Tengberg (Muséum National d’Histoire Naturelle, Paris, France). The atlas developed from this reference collection, including identification keys, microphotograph of wood thin sections and charcoal and description of features observed is provided as supplementary material.

#### Quantification of charred remains

Common indices were used to evaluate the assemblages, namely ubiquity, density (total weight of charred material from all fractions per litre of sediment floated), percentage of undetermined fragments (over the total of fragments analysed) and Simpson’s diversity (the presence and distribution of a specific taxon over the entire sample) [66–67].

### Phytoliths

Phytoliths were extracted from sediments using a protocol adapted from Madella, Powers-Jones, and Jones [68]. Slides with permanent mounting (Entellan®) were observed under a Nikon Labshot 2 transmitted light microscope at 200X and 630x magnifications. Phytoliths were identified through comparison with published material [e.g. 69–70] and a reference collection of phytoliths from the leaves of local species created by the author [49]. A minimum of 350 single-cell phytoliths were identified for each sample and multi-cell phytolith (silica skeletons) were counted separately [71–73].

#### Phytolith concentration and quantification

Concentration of phytoliths per gram of Acid Insoluble Fraction (AIF) was calculated according to [74]. The degree of correlation between phytolith concentration and number of morphotypes identified was used as a proxy for the effect of taphonomic processes on the phytolith assemblages. High correlation between indices, implying that the concentration affected the number of identified morphotypes, indicates that taphonomic processes might have influenced the general composition of the phytolith assemblages [75].

### Spherulites

Slides for spherulite observation were prepared by rubbing a small quantity of sediment on a 47 μm mesh and the residue was mounted with permanent mounting medium (Entellan®). Spherulites were observed under the same microscope as phytoliths and counted over the entire slide [76].

### Multi-element chemical analyses

Multi-element data was obtained using Inductive Coupled Plasma Atomic Emission Spectroscopy (ICP-AES) with an aqua regia digestion, and was performed by ASL Laboratory Group, Seville (Spain). This method analyses the concentration of 35 elements, and elements that did not reach reliable instrument detection limits (IDL) in the majority of the samples were excluded from the analysis. To normalise the distribution of chemical data and to obviate the differences in scale of measurements produced by the instrumental analysis, the value of the chemical elements were transformed to LOG10 values prior to analysis.

### Statistical analyses

Several indices were used to assess the impact of taphonomic and post-depositional processes on both charcoal and phytoliths, and also to quantify results and integrate the different lines of evidence. Statistical analysis was carried out using the R [77] and SPSS statistical software packages. The data on the variables presented a mix of normal and non-normal distributions, so both parametric and non-parametric tests were used (e.g. Spearman’s rho was used to calculate correlations, and ANOVA was used to analyse variations in the mean values).

Correspondence Analysis (CA) was performed on charcoal and phytoliths combined datasets in order to explore the association of the variables under study in a two-dimensional plot and highlight the presence of groups within the data. Anthracological remains were grouped by ecological significance (Table 2) and phytoliths in morphological groups representing different plant parts. Results were grouped by site and context type, averaging all samples’ counts and rounding them to the nearest integer. Principal Component Analysis (PCA) was performed on the multi-element chemical results combining archaeological contexts and dung reference collection material [10]. R codes and datasets for both CA and PCA analyses available as supplementary material.

**Table 2:** Wood species mentioned in the text with ubiquity test scores for each site according to floras of South Asia [43, 78].

			Ubiquity scores (%)			
Family	Genus and species	Ecology	Harappa	Kanmer	Shikarpur	Alamgirpur
FABACEAE	Acacia Senegal (L.) Willd.	DRY THORN SCRUBLAND	6.9	50	13	—
MELIACEAE	Azadirachta indica A. Juss.		6.9	80	47	6.7
ZYGOPHILLACEAE	Balanites aegyptiaca (L.) Delile		—	—	—	13.0
ASCLEPIADACEAE	Calotropis procera (Aiton) W.T. Aiton		13.8	50	7	—
CAPPARACEAE	Capparis decidua (Forssk.) Edgew.		3.5	80	53	27
LAMIACEAE	Clerodendrum sp.		6.9	20	—	—
BORAGINACEAE	Cordia sp.		—	10	—	—
ASCLEPIADACEAE	Leptadenia pyrotecnica (Burm. f) Juss. ex Schult.		0.5	—	7	—
FABACEAE	Prosopis cineraria (L.) Druce		20.7	50	13	—
FABACEAE	Prosopis farcta (Banks & Sol.) J.F. Macbr.		20.7	—	—	—
SALVADORACEAE	Salvadora oleoides Decne		55.2	10	20	—
SALVADORACEAE	Salvadora persica L.		48.3	50	27	6.7
FABACEAE	Senna siamea (Lam.) H.S.Irwin & Barneby		—	20	—	—
CHENOPODIACEAE	Suaeda monoica Forssk.		3.5	—	—	—
MORACEAE	Ziziphus mauritiana Lam.		24.1	10	27	—
MORACEAE	Ziziphus nummularia (Burm. F.) Wight & Arn		17.2	50	27	—
FABACEAE	Acacia nilotica (L.) Delile *	RIVERINE	17.2	60	40	—
FABACEAE	Dalbergia sissoo Roxb. Ex DC.		3.5	10	—	—
SALICACEAE	Populus euphratica Oliv.		6.9	—	—	—
TAMARICACEAE	Tamarix aphylla (L.) Karsten *		79.3	10	—	—
ACANTHACEAE	Avicennia marina (Forssk.) Vierh.	MANGROVE	—	40	—	—
RIZOPHORACEAE	Bruguiera gymnorrhiza (L.) Lam		—	—	7	—
PINACEAE	Cedrus deodara (D. Don.) G. Don	MOUNTAIN FOREST	3.5	—	—	—
ADOXACEAE	cf. Viburnum sp.		3.5	—	—	—
MORACEAE	Morus macroura Miq.		3.5	—	—	—
PINACEAE	Pinus sp.		27.6	—	—	—
FAGACEAE	Quercus sp.		3.5	—	—	—
MORACEAE	Ficus sp.	ECOLOGICALLY NOT MEANINGFUL	—	10	—	—
MYRISTICACEAE	Myristica cf malabarica Lam.		—	—	—	—
OLEACEAE	Olea ferruginea Royle		—	—	—	—
ARECACEAE	Phoenix dactylifera/sylvestris		24.1	10	—	—
ANACARDACEAE	Pistcia integerrima J. L. Stewart ex Brandis.		6.9	—	—	—
APOCYNACEAE	Wrightia tinctoria (Roxb.) R. Br.		—	10	—	—

* These two species have been included in this category as, although they can grow far form watercourses they tend to thrive in presence of moist soils (personal observation in the field).

## Results

### Taphonomy and representativeness of results

#### Charcoal

The percentage of charcoal fragments > 2 mm in size retrieved from all sites was very low. Therefore, all fragments > 1 mm were analysed, coming to a total of over 4300 fragments. The small dimensions of the charcoal fragments from all sites and contexts and the general scarcity of charred remains suggest a high level of post depositional taphonomic influence. Indeed, values for Density Index were consistently low at all sites and the percentage of undetermined fragments was generally high, with floor samples from Alamgirpur reaching up to 25% (Table 1). There is, however, no correlation between diversity in the assemblages and number of underdetermined fragments, or between density and diversity, at any of the sites (note that only negative correlations are significant here as the Diversity Index goes from 0—no diversity- to 1—maximum diversity). This pattern indicates that generally taphonomy does not affect the representativeness of the assemblage regarding species composition, except in the case of pit samples from Harappa, and occupation level samples at Kanmer and Shikarpur.

#### Phytoliths

Phytolith concentration was very variable ranging from less than 100 k to over 27 M of phytoliths per gram of AIF. Pit and floor samples from Alamgirpur display the highest concentration of phytoliths, whereas street deposits at Harappa show the lowest. Overall, Harappa presents a concentration of phytoliths notably lower than the other three sites (average just over 640 k against 10 M at Kanmer and Shikarpur and 23 M at Alamgirpur). The low concentrations at Harappa do not seem to be an effect of taphonomic processes as there is no overall correlation between the two measurements (global r^2^ for Harappa is 0.15), although some contexts show a correlation between the phytolith concentration and the number of morphotypes identified. No positive correlation between phytolith concentration and number of morphotypes identified has been found at any of the sites, indicating that taphonomy does not affect the representativeness of the samples [75].

### Environment and vegetation

**Fig 2:**
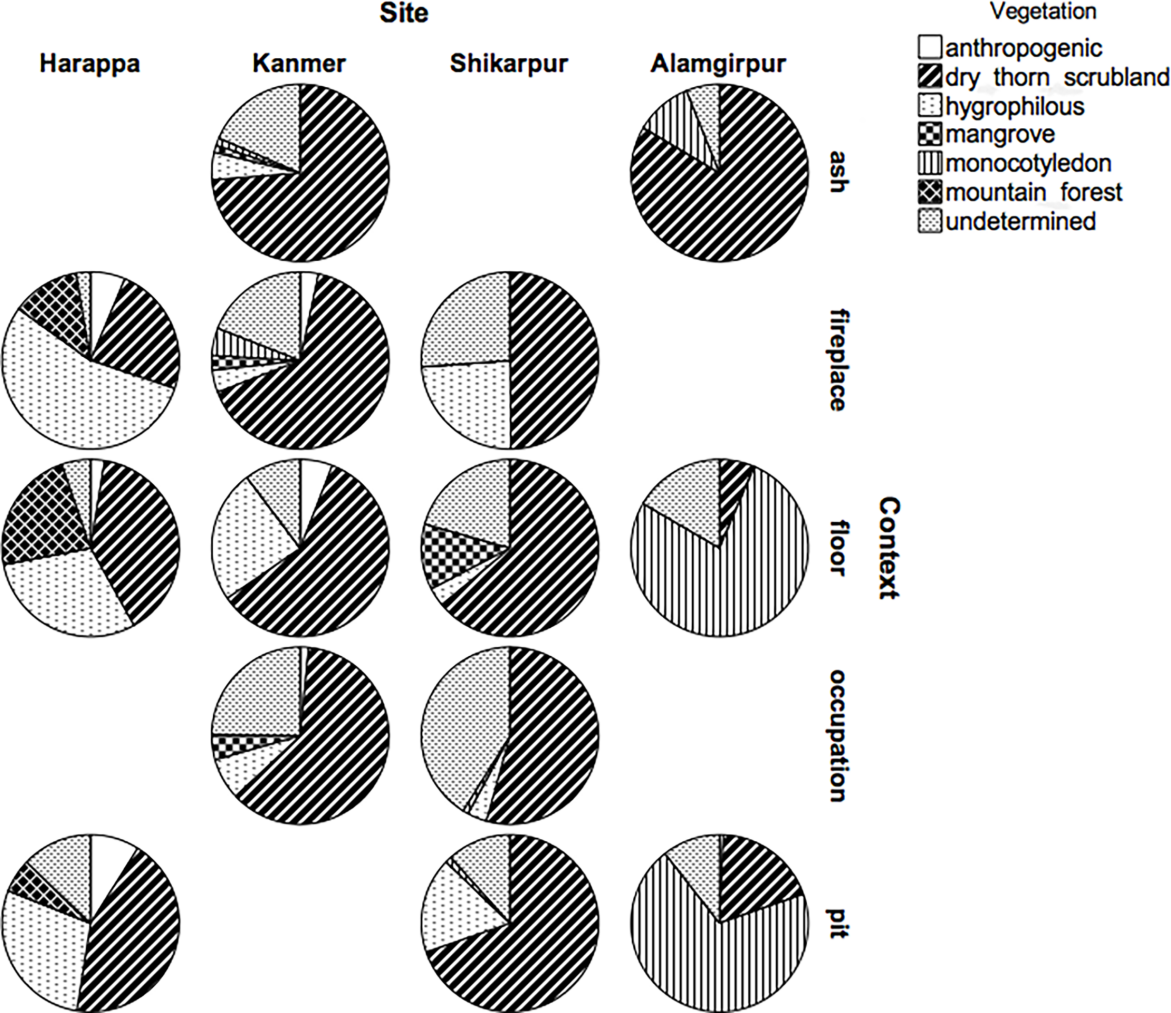
Pie charts summarising the results of the anthracological analyses. The charts represent the proportion of different types of vegetation exploited at the 4 study-sites divided by context-type.

It is notable that the anthracological assemblages are very similar throughout the four sites analysed in that species from the dry-thorn scrubland formation, dominant in all of these regions, represents between 23 and 62% (Fig 2 and Table 2). There are, however, some clear differences in the assemblages due to the specific geography of each area. For example, at Harappa, which is the northernmost site and is located near a major river system, the assemblage contains significant quantities of hygrophilous (44%) and Himalayan species (13%). Kanmer and Shikarpur present an almost identical assemblage in terms of the presence/absence of specific species, and dominated by dry-thorn scrubland species with few examples of hygrophilous (between 7 and 13%) and exotic taxa (between 2 and 4%). At Alamgirpur, dicotyledonous wood belongs almost exclusively to the dry-thorn formation (99% of the charred wood fragments identified), but monocotyledon remains dominate the assemblage (52% of the entire charred remains analysed).

### Fuel

#### Charcoal

A wide range of species were used as fuel at the four sites analysed. At Harappa, 25 species out of the 30 identified at the site were encountered in fireplaces. At Kanmer and Shikarpur 13 different species were present in fireplaces and ashy patches out of the 22 identified in Kanmer’s samples and the 17 encountered in Shikarpur’s. Alamgirpur represents an exception with almost 90% of the fragments identified belonging to a single species (*Capparis decidua* [Forssk.] Edgew.). However, at Alamgirpur wood does not seem to be the primary source of fuel as the assemblage is dominated by non-wood charred material, mainly represented by an unidentified monocotyledon (different from *Phoenix* sp). Although samples from Harappa yielded the highest taxa count, the diversity index of fireplaces from Kanmer is actually much higher (for fireplaces Kanmer = 0.75, Harappa = 0.53). The majority of fragments identified in fireplace samples from Harappa belong to hygrophilous species (Fig 2), whereas fireplace samples at Kanmer and Shikarpur and ash samples at Alamgirpur are composed mainly by dry-thorn scrubland species (Fig 2).

#### Phytoliths

**Fig 3:**
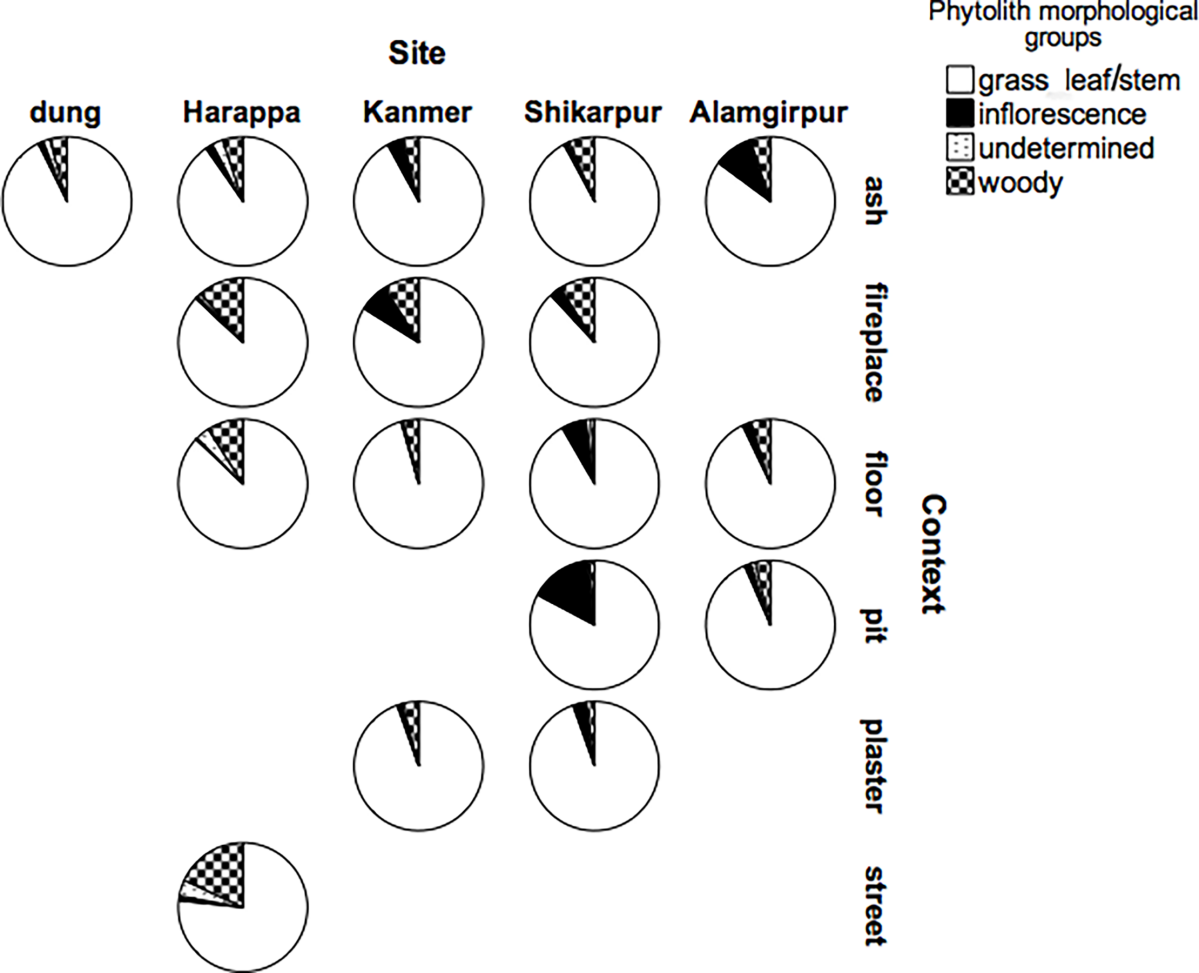
Pie charts summarising the results of the phytolith analyses. The charts represent the proportion of different phytoliths groups identified at the 4 study-sites divided by context-type.

Single morphotypes (identified either as single cells or part of multicells phytoliths) were grouped for analysis in morphological groups, representing different parts of grasses or groups of plants, (according to the groups published in [10]). Grass leaf/stem indicators represent the dominant phytolith group at all sites and in all samples (Fig 3). Notable exceptions to the average are Harappa street samples, where woody plants morphotypes are highly abundant, and Shikarpur pit samples that present a high number of inflorescence morphotypes.

#### Spherulite and chemical analyses

**Fig 4:**
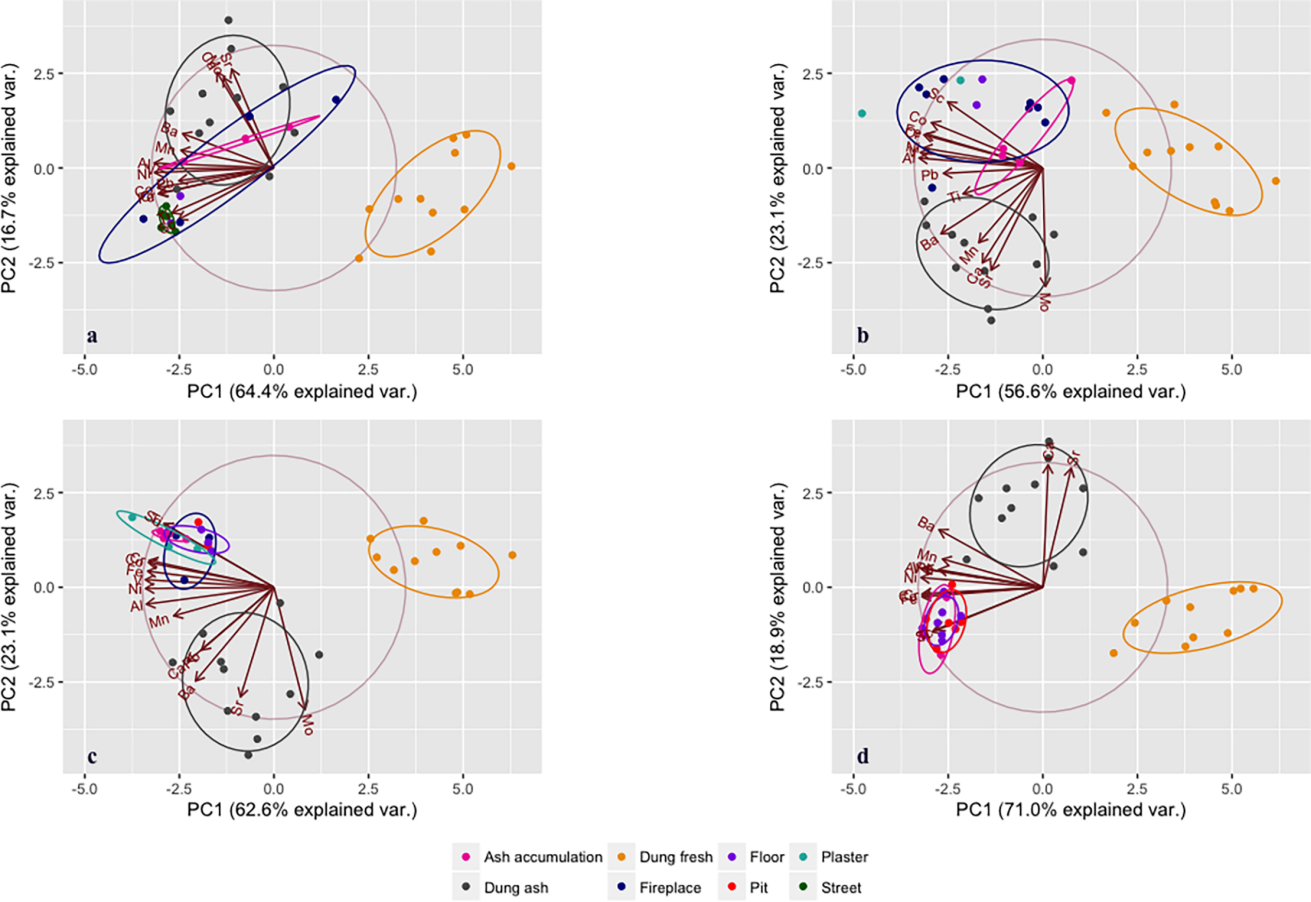
PCA biplots of multi-chemical analyses. The plots show the results of PCA analyses on multi-chemical data: a) Harappa, b) Kanmer, c) Shikarpur and d) Alamgirpur.

Spherulites are relatively scarce in the sediment samples analysed. Although 50% of samples from Kanmer contain spherulites, very few samples present spherulites at all other sites. Spherulites are common in fireplaces at Kanmer (7/8 samples) and relatively common at Harappa (2/4 samples), but only one fireplace from Alamgirpur and none from Shikarpur contained spherulites. To further clarify whether dung was being exploited, multi-element chemical analysis was performed on a combination of elements (Al, Ba, Ca, Co, Cr, Fe, Mn, Mo, Ni, Pb, Sc, Sr, Ti, V) previously identified as being characteristic of dung in the area under study [10]. PCAs show that in general samples tend to cluster with or close to dung ash (Fig 4). Particularly noticeable, at Harappa all ash accumulations and two of the four fireplaces cluster together with dung ash samples. At Kanmer, all samples show similar variance and are correlated on the first principal component, with one of the fireplaces acting as an outlier and approximating to dung ash. At Shikarpur and Alamgirpur, all samples show correlation both with dung ash (on PC1) and fresh dung (PC2) without clustering with one or the other.

#### Multivariate statistics of charcoal and phytoliths

**Fig 5:**
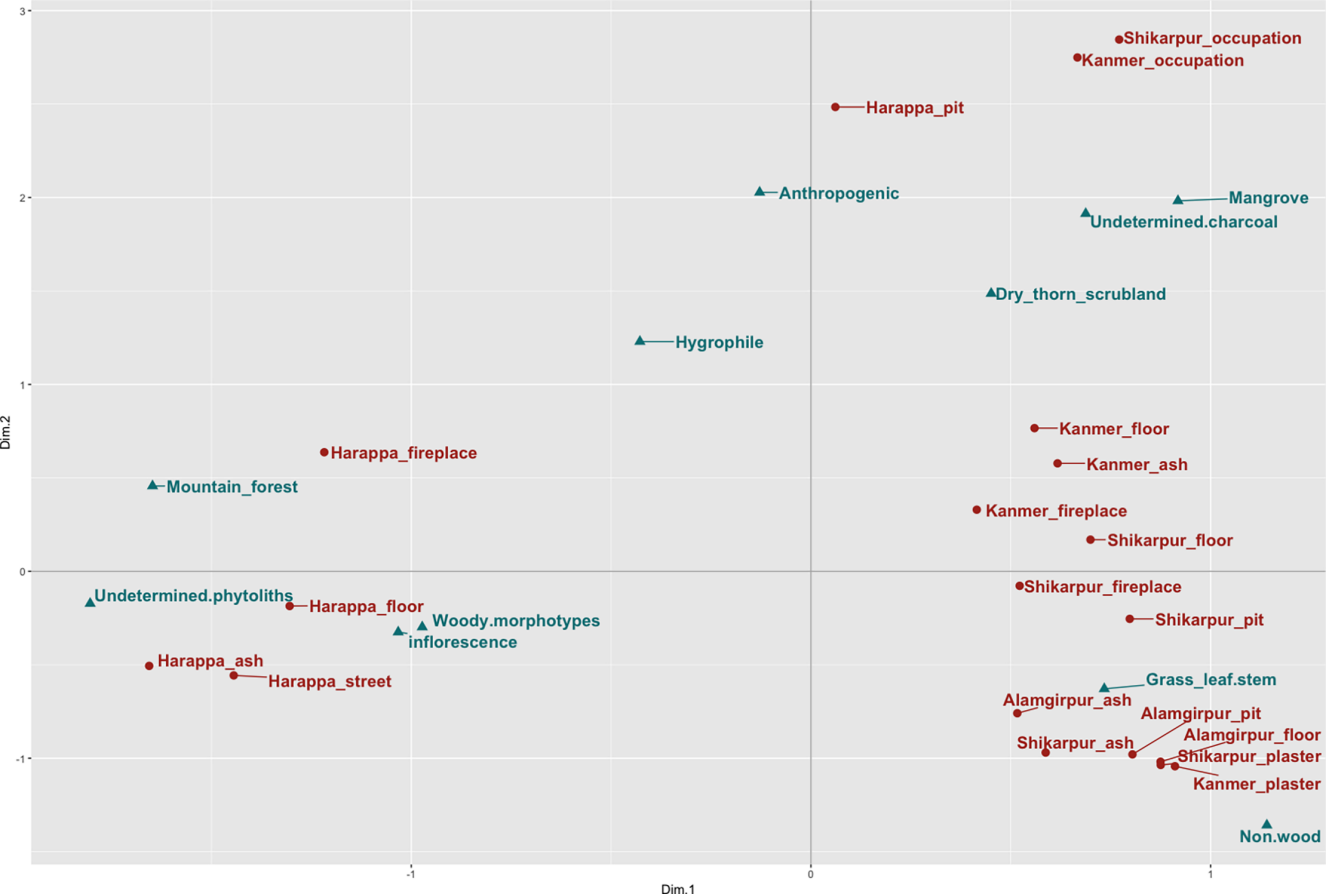
Correspondence analysis biplot. The plot shows the association between site/context and the type of wood charcoal or phytolith categories that most characterize them.

Correspondence analyses (Fig 5), combining both anthracological and phytolith results presents a picture that differs slightly from that evidenced by looking at each category of data separately. For example, at Harappa mountain species represented mainly by the coniferous taxa, *Pinus* sp. and *Cedrus deodara* ([Roxb.] G.Don) are closely associated with fireplaces. At Kanmer and Alamgirpur, phytoliths (grass leaf and culm indicators) and dry-thorn scrubland species are associated with fireplaces.

## Discussion

### Diversity of fuels during the Harappan Phase: Not all that burns is wood

#### Firewood exploitation

The contribution of anthracological analysis to the study of fuel exploitation and use is undoubted. Wood was the most important fuel resource in prehistory and charcoal recovery through flotation or dry sieving is pivotal to the understanding of past environments and human-environment interactions. It has been argued that charcoal retrieved from archaeological contexts always represents an anthropic choice and does not necessarily reflect the entire composition of the vegetation present around the site at that time [e.g. 79–80]. On this basis, charcoal retrieved from archaeological contexts can only be used with caution as a proxy to reconstruct past environments and delineate climatic changes. On the other hand, because archaeological charcoal assemblages are so strictly dependent on human choice, this material has great potential for investigating human behaviour.

The four case-study sites are located in different regions and, although they are situated on different river-systems, they are all within the same vegetation zone, characterised by the tropical thorn forest formation. Therefore, similar patterns of fuelwood exploitation (i.e., similar anthracological assemblages) would be expected. Indeed, anthracological analyses have shown that most of the wood exploited belongs to species of the arid dry-thorn scrubland formation (Fig 2). Evidently, during the Harappan period this vegetation type was easily accessible and widely exploited for fuel resources. Nevertheless, each site also provides evidence of specific adaptations to the local environments and the exploitation of a variety of wood resources at small scale. For instance, hygrophile species played an important role as fuelwood at Harappa (Fig 2), and these are likely to have been collected in the riparian forest that bordered the course of the Ravi, which flowed in close proximity to the site (exploitation of the riverine environments by Indus populations is confirmed by archaeozoological studies [81]). At the same time, wood originating in distant places was also exploited, as indicated by the abundance of deciduous and evergreen, mountain species at Harappa (Fig 2). These species have been found predominantly in floor deposits, but they were also found in fireplaces, which means that they were either used as fuel (e.g. for specialised fires) or became fuel after having been exploited for a different purpose. The exploitation of mountain forest environments can perhaps be linked to internal trade within the orbit of the Indus Civilisation. Indeed, Indus populations exploited several raw materials from mountain regions including lead, steatite, grounding stones of various types, alabaster, gold and silver [5, 82]. Wood could have been potentially exploited in conjunction with these raw materials and transported to Harappa along the same trading networks. It is clear that local wood resources were available, and the presence of mountain species indicates that they were intentionally imported for some specific purpose. Some species of conifer like *Pinus* sp. can be used to produce high temperature fires due to the high content of resin [83–84]. Coniferous wood is also considerably softer to cut than deciduous species, and is thus easier to exploit when tools were made using a relatively soft metal like copper or bronze. It is also possible that the exploitation of mountain wood resources may have been a response to some pressure on the local environments that reduced the availability of easily accessed quality wood supplies. The possibility that there was resource impoverishment is indicated by the increased presence of *Tamarix* sp. in the charcoal assemblage analysed in this study, towards the end of the Indus urban period (period 3C according to Kenoyer’s chronology). Although tamarisk grows preferentially along watercourses on loamy soils, it is also very resistant to high salinity and aridity, and it dominates in depleted environments as it is usually the last species to disappear in the processes of deforestation in arid and semi-arid zones [85]. The assemblages of Kanmer and Shikarpur are indicative of similar situations, with evidence for different environments being exploited at the same time (Fig 2). At both sites, the majority of species identified belong to the dry-thorn scrubland formation. However, a considerable number of fragments belong to riverine formations, and coastal mangrove species are also present. In both cases, charcoal assemblages provide some evidence of palaeoenvironment, as at present there is no trace of riverine or mangrove formations near the site. In addition, one non-local species was identified at Kanmer, *Myristica* sp., which grew on the Maharashtra coast some 500 km to the southeast. As in the case of mountain species at Harappa, it is unclear whether this wood was imported specifically to be used as fuel. Unlike the evidence from Harappa where at least one fireplace showed a predominance of exotic taxa, at Kanmer the presence of non-local species was extremely rare. It is perhaps most likely that these woods were not imported to be used as a fuel but moved along the trade networks (as discussed for Harappa) as small objects or tools together with other goods. Alamgirpur presents an interesting dataset that is different to that of the other sites (Fig 2). Given its location in northwest India near the Hindon River and close to the Yamuna and Ganges Rivers, it was expected that fuelwood would have been more abundant, but anthracological analysis has revealed a scarce use of wood and a predominance of alternative fuels. Although most of the charcoal fragments analysed at Alamgirpur derive from contexts that are only indirectly related to fuel use (*i*.*e*. floors and pits), the constant and almost exclusive presence of monocotyledon remains coupled with the absence of other wood remains, indicate that people at Alamgirpur during the Harappan period could not, or choose not, to use wood, but burned alternative fuels (either the monocotyledon, directly or after having used it for other purposes, or dung). As above, a possible interpretation of this behaviour is human pressure on the environment and resource depletion. Archaeological research and surveys conducted in this region suggest that it is unlikely that population pressure (in terms of number of people) in the area was so high as to cause a drastic depletion of fuelwood resources [86]. Another possible explanation is connected to the management of the landscape for agricultural purposes. Indeed, the predominant species of wood identified, *Capparis decidua* (Forssk.) Edgew., is extremely tolerant to fire and that, together with the constant presence of monocot remains, suggests the use of fire to manage the vegetation surrounding the site.

#### Dung as a fuel resource

Amongst the alternative fuel types, dung in the form of dung cakes or patties, was likely to have been a common resource. Bovine remains (including both *Bos indicus* L. and *Bubalis bubalus* L.) are common at Indus sites [e.g. 87–92], and dung is widely used in India today as a supplement or main domestic fuel. Experimental firing of clay figurines in a small kiln has shown that dung fuel is capable of reaching a temperature of 107°C in 20 minutes, over 1000°C in 1 hour, and 1095°C in 1 hour and 20 minutes [2]. These capabilities make dung an ideal fuel source for pottery firing activities, which is a practice that has been observed ethnographically for example in northern Maghreb [93–94] and Sindh, Pakistan [95] where pottery is still traditionally fired with dung fuel either alone or in combination with other non-ligneous materials. The fuel chamber of the kiln structure discovered in trench Z17 at Kanmer presented a deposit composed only of dung ash (according to previous analyses performed on dung reference material [10]). Although the function of this particular kiln is not as yet clear, its shape and location indicate that it was not a domestic fireplace for food preparation. Typologically the kiln is akin to the structures used for bead production and the different fuel layer identified inside the main and secondary fuel chambers have some ethnographical parallels with bead production and firing in modern Khambat (personal observations). Remains of dung were present, in varying concentrations, in almost all the samples examined from all sites. In many cases, in firing contexts deposits such as fireplaces and ash accumulations, dung was found mixed with wood or crop processing leftovers, but occasionally phytolith and chemical analyses data combined with the lack of charcoal showed that dung was the main constituent of the archaeological sediment analysed. The archaeological study of fuels alternative to wood has concentrated mostly on dung [10, 21, 94, 96–99], whereas the use of crop-processing leftovers has seldom been investigated. At this point, it needs to be stressed that even with a sound methodology to infer the presence of dung in archaeological sediments, its quantification is still an unsolved problem. An increase in the number of features that display dung signatures might be interpreted as an escalation of dung use. However, it needs to be remembered that fireplace deposits often represent one or more episodes of burning and can therefore be misleading in terms of long-term fuel choice. Indeed, it would be impossible to exclude the possibility that an analysed sample is not the result of an occasional burning of dung in one location. On the contrary, in pits and ash accumulations, which represent long-term discard of fuel refuse, a possible signature for dung might be confused by the heterogeneous origin of these constituent material. However, the widespread use of dung as fuel over several contexts and several sub-phases strongly suggests that wood alone was not sufficient to fulfil the needs of fuel resources during the Harappan Phase at all the study-case sites.

#### Crop processing leftovers: Were they used as fuel or not?

The identification of the direct use of crop processing by-products as fuel in archaeological deposits is not straightforward. The first stage of cereal processing, *i*.*e*. the elimination of culms and leaves, produces a phytolith signature that is similar to that of dung, which is the product of animals being fed on or eating the same plant parts [10]. Therefore, the discrimination between the two must rely on other methods or additional considerations. In fact, it can be argued that in arid countries, where grassland is scarce and grazing material in short supply, culms and leaves are more valuable as fodder than as fuel, considering their low calorific power. On the other hand, refuse from the second stage of cereal processing of hulled cereals, *i*.*e*. husk and chaff, is rarely used to supplement animal diet because of its high content of opal silica [72, 100]. Such remains can enter the fuel cycle separately via a non-dung route, and helpfully they can easily be distinguished as they leave a very distinctive phytolith signature. However, in the case of free threshing cereals, husk and chaff are released during the threshing and first winnowing stages and are therefore mixed with leaves and culms. During the Harappan Phase, second stage crop processing by-products were used as fuel, but only occasionally, and they were not as important a fuel as wood or dung.

#### What goes where: Differential fuel use according to firing activity

One of the questions explored by this paper was the possibility of identifying different fuel types used for different firing activities. As demonstrated by the analysis of the kiln structure exposed in trench Z17 at Kanmer, not only is it possible to distinguish between different fuels in different firing structures, but also it is possible to differentiate individual firing episodes in the same structure. Indeed, in this structure at Kanmer, different episodes of burning were identified and revealed the use of different fuel types: including wood, dung, and a mixture of wood and dung [10]. In addition, among the samples from Harappa, one fireplace has been identified where conifer and mountain species were used almost exclusively. This finding suggests that a specific choice was made to use non-local and probably expensive fuelwood. As no indication was given of the possible function of this fireplace we can only speculate on the reason why this choice was made. One of the possible explanations is that this was just an extraordinary episode and that the charcoal retrieved from this context is the result of a one-time burning of, maybe, discarded objects. However, another possible explanation involves the deliberate, intentional use of these species for an activity that required a particular type of fuel. This could have happened either for practical reasons (e.g. temperature, smoke, scent etc.) or for cultural motives, such as the use of firing structure for rituals and religionous practices. Indeed, in modern India and Pakistan, the use of plants for cultural or religious activities is very common [101–102] and it seems that some of these plants had a particular importance in the past as well, as they are found depicted on pottery or carved on seals (see for example the discussion on the importance of the pipal tree [*Ficus religiosa* L.] by Parpola [103–105].

### Socio-ecological dynamics in northwest South Asia during the Harappan phase (2500–1900 BC)

The evidence obtained from the study of the four archaeological sites under discussion will now be reviewed in terms of comparison of fuel exploitation and use strategies. During the Harappan Phase Indus populations exploited a range of different fuel resources sourced from all the vegetation types present in the area where the settlement was located but also relying on alternative types of fuels. This exploitation pattern is broadly homogeneous throughout the regions studied, in the sense that a mixing of local species and fuels alternative to wood are the principal sources of fuel. To this core set of choices, however, some non-local species were added that not necessarily being imported specifically to be used as a fuel, but that nonetheless entered the burning cycles as well. The sites analysed are located roughly at the southern, northern and eastern limits of the area occupied by Indus populations during the Harappan period (Fig 1). If one agrees with the assumption that wood is the preferred source of fuel for its burning qualities, the presence of fuels alternative to wood in almost every archaeological sample analysed suggests a scarcity of fuelwood. This, together with the evidence of similar strategies of wood exploitation within the entire area studied, implies that human impact on woodland resources was substantial during the Harappan period. The assessment of this impact and especially the evaluation of how human exploitation of woodland resources has influenced and changed the natural landscape can only be tentative when looking at a single chronological phase. Nevertheless, this work provides interesting information about how people exploited and used fuel during the Harappan period, as the study of a fixed time period offers the opportunity to compare different settlement types.

#### Big cities and small towns: Dealing with different yet similar necessities

There are clear archaeological differences in size and function between the case-study sites. Although it has been demonstrated that the strategies of resource exploitation were overall similar at all sites analysed, a marked difference was found in the amount of charcoal retrieved. Keeping in mind the general low density of charcoal at all sites, it is evident that at Harappa and Kanmer flotation produced higher volumes of wood charcoal than at the other sites (with density in the range of 2 decimals against 3 decimals, Table 1). It is likely that burning conditions affecting the formation of the charcoal and the choice of fuels are the principal factors involved in the creation of differences in the amount of charcoal retrieved from the archaeological excavations. The formation and preservation of charcoal during the firing process depends largely on the burning atmosphere: charcoal is formed and preserved in reducing conditions, but in oxidising conditions charcoal transforms into ash [106]. The quantity of charcoal retrieved therefore can be just an indication of the burning conditions present at the moment of its formation. However, as the contexts analysed were varied, it can be assumed that the burning conditions were different and/or random in each context and therefore general comparisons can be drawn between sites. Moreover, the average number of species identified is higher at Harappa and Kanmer than in the other two sites. As these data do not reflect preservation conditions or the number of samples analysed (Table 1), it can be concluded that the two bigger settlements exploited a wider range of wood fuel resources than the smaller settlements. In fact, it is highly probable that the strategies of exploiting woodland resources changed according to the level of social and economic organisation of a settlement. Thus, in smaller settlements, like Shikarpur and Alamgirpur, the collection of fuelwood was likely carried out at the family level with each household supporting its own needs. However, in bigger urban centres like Harappa, where the simple act of reaching the woodland could have taken longer time, and where people with more resources could afford it, there might have been people dedicated to the procurement of fuel from the hinterland for others in the population. In this respect, the comparison with the evidence of water management and crop processing activities is particularly interesting. Archaeological evidence suggests that water was managed at a household or local level in big centres whereas small settlements relied on communal water sources almost certainly managed by dedicated people (for a general discussion see for example [2, 4–5]. On the other hand, specialised people outside the urban cities carried out crop processing as opposed to small settlements where crop processing happened inside each household [65, 107–108]. Archaeology has shown that water was very important for the Indus Valley Civilisation and that all major cities had a complex system of drainages, wells and water storage. From the archaeological evidence it seems as if in the big urban centres water resources were widely distributed within the site and, if not at the household level, at least at a neighbourhood level. Small settlements do not show this type of evidence, probably because each family could easily obtain water from off-site structures (basins, small rivers, wells etc.). However, it is also possible that specialised people transported water from tanks into the site as happens nowadays in Gujarat (personal observations in Kanmer, January-March 2008). Weber [65] first introduced the idea of a shift from large-scale field crop processing in the Mature Harappan to household crop processing during the Late Harappan Period. Recent analyses by Bates et al. [107] on macrobotanical remains at different rural sites in Haryana, shows that household processing was carried out also during the Mature Harappan and the same conclusion is confirmed by the phytolith analyses conducted for the present work. Indeed, at Shikarpur the amount of inflorescence phytoliths found in floor deposits was six times higher than what was found at Harappa (Fig 3). This pattern indicates that the final stages of crop processing were carried out in the house at Shikarpur. The situation does not appear to be the same at Alamgirpur, where inflorescence phytoliths on floor deposits comprise only 2% of the assemblage (which in any case is double the amount found at Harappa; Fig 3). However, it has been shown how the fuel exploitation at Alamgirpur was different than at the other sites because of a possible scarcity of trees, and thus people at this settlement used all the available resources as fuel. This conclusion is also supported by the fact that at Alamgirpur inflorescence phytoliths were very abundant in ash deposits, suggesting the use of this resource as a fuel. The data show that depending on the type of settlement under study, the picture of the intensity of resource exploitation can be very variable.

## Conclusions

This paper has explored the relationship between people and their environment during the Harappan period of the Indus Civilisation (2500–1900 B.C.) by analysing fuel exploitation and use strategies. Wood and other plant materials (chaff, straw, etc) played a pivotal role in all Early Civilisations as fuel for domestic and industrial uses. The continuous and extensive exploitation of fuelwood has the potential to negatively impinge on the natural environment, especially in arid countries where woodland is scarce. This is particularly true during periods of rapid urban expansion and population growth when the demand for wood resources is high. In these cases, alternative sources of fuel, such as dung or crop-processing leftovers, become vital and their widespread use, associated to specific variations in the wood assemblage, provide hints to assess the human impact on the environment.

Fuelwood is relatively easy to detect in the archaeological record but alternative forms of fuel, such as dung and crop processing leftovers, do not leave a clear signature. For this reason, a specific methodology involving the study of charred wood, phytoliths, spherulites and geochemistry has been applied. The combined application of these different but related techniques offers the means of clearly discriminate between the different fuel resources, thus evaluating their relative importance.

Four case-study sites have been investigated: Harappa (Pakistan), Alamgirpur (Uttar Pradesh, India), Kanmer and Shikarpur (Gujarat, India). These sites are all located in an arid to semi-arid region, but they are situated in slightly different zones (hyper-dry, hot semi-arid and hot moist semi-arid), facilitating an assessment of local adaptations. In addition, the four sites represent different settlement types which were likely distinct socio-economically: from the big urban centre of Harappa to the small village of Alamgirpur, which is situated at the eastern border of the area occupied by the Indus populations. At all four sites the overall strategies of fuel exploitation were similar, concentrating on local, easily available resources, but in each case “exotic” taxa were also exploited. In terms of types of fuel used, at three sites, Harappa, Kanmer and Shikarpur, wood was used alongside alternative fuels such as dung and probably crop processing leftovers. Alamgirpur presents a different situation where the most commonly used fuel was not wood, but some form of cane. In addition, this study provides new knowledge on the ecological settings in which the Mature Harappan period settlements evolved. It suggests that there was environmental stability at Harappa and Alamgirpur, and a dynamic hydrological situation in Gujarat.

## Supporting information

S1 Catalogue Wood and charcoal reference collection catalogue. (PDF)

S1 Dataset Harappa: Charcoal analyses raw data. (XLS)

S2 Dataset Kanmer: Charcoal analyses raw data. (XLS)

S3 Dataset Shikarpur: Charcoal analyses raw data. (XLS)

S4 Dataset Alamgirpur: Charcoal analyses raw data. (XLS)

S5 Dataset Phytolith analyses raw data. (XLS)

S6 Dataset Spherulite analyses raw data. (XLS)

S7 Dataset Multi-element analyses raw data. (XLS)

S1 Folder R scripts for CA and PCA with datasets used for statistical analyses. (ZIP)

S1 Protocols Laboratory protocols used in this study. (DOC)
