# Supplementary material for: 'Not all that burns is wood'. A social perspective on fuel exploitation and use during the Indus urban period (2600-1900 BC)
Source: PLoS One. 2018 Mar 7;13(3):e0192364. doi: 10.1371/journal.pone.0192364 (PMC5841642; doi:10.1371/journal.pone.0192364)
Supplement: S1 Catalogue — (PDF) [file pone.0192364.s001.pdf]

S2: Wood and charcoal catalogue

This Appendix presents the work on the wood and charcoal reference collection. The descriptions are based on the IAWA list of characters for wood identification (Wheeler et al., 2007). All the descriptions were based on the observation of fresh wood thin sections except for *Ziziphus mauritiana*, which description was performed on the charcoal sample. Features underlined were observed in the samples but are not present in the Inside Wood Database description (Inside Wood Database, 2004-onwards); features between brackets are present in the Inside Wood Database descriptions but were not observed in the samples.

REFERENCES

INSIDE WOOD DATABASE (2004-onwards), *Inside Wood Database*, http://insidewood.lib.ncsu.edu/search.

WHEELER, E. A., BAAS, P. & P.E., G. (Eds.) (2007) *IAWA List of Microscopic Features for Hardwood Identification by an IAWA Committee,* IAWA Bulletin 10(3), 219-332.

IDENTIFICATION KEYS

**1 Rays homogeneous**	go to n. 2
**Rays heterogeneous**	go to n. 16
**2 All ray cells procumbent**	go to n. 3
**All ray cells upright/square**	go to n. 13
**3 Rays of to distinct classes**	go to n. 8
**Rays of one class**	go to n. 4
**4 Ray width 1-3 cells**	go to n. 10
**Ray width 4-10 cells**	go to n. 5
**5 Ray height >1 mm**	*Acacia leucophloea*
**Ray height <1 mm**	go to n. 6
**6 Growth ring boundaries distinct**	*Acacia catechu*
**Growth ring boundaries indistinct**	go to n. 7
**7 Fibres very thick walled**	*Acacia ferruginea*
**Fibres thin- to thick-walled**	*Acacia nilotica*

8 Vessels clusters common……………………………………….……… go to n. 9

Vessels solitary or in short radial rows………………………… *Acacia senegal*

9 Fibres very thick walled and axial parenchyma in bands….... *Acacia farnesiana*

Fibres thin- to thick-walled and parenchyma vasicentric…… *Capparis decidua*

10 Vessels clusters common and in tangential bands………….. *Azadirachta indica*

Vessels in radial multiple of 4 or more……………………………… go to n. 11

11 Fibres very thick-walled……………………………………………. go to n. 12

Fibres thin-to thick walled………………………………….. *Prosopis cineraria*

12 Axial parenchyma vasicentric, aliform and in irregular bands………*Senna auriculata*

Axial parenchyma in regular bands, sometimes scalariform and vasicentric parenchyma almost absent…………………………………………… *Senna siamea*

13 Rays uniseriate…………………………………..…………….. go to n. 14

Rays multiseriate……………………………………..………….. go to n. 15

14 Rays height > 1mm and axial parenchyma unilateral paratracheal and in bands up to 3 cells wide……………………………………….. *Ziziphus nummularia*

Wood not as above……………………………………........ *Ziziphus mauritiana*

15 Rays present………………………………………………………. *Salvadora oleoides*

Wood rayless……………………………………………………………. *Suaeda monoica*

16 Ray cells procumbent with marginal rows of upright/square cells go to n. 17

Procumbent, square and upright cells mixed throughout the ray go to n. 22

17 2-4 and mostly over 4 rows of marginal cells………………………… go to n. 18

1 row of marginal cells………………………………………………… go to n. 19

18 Ray width 1-3, fibres very thin walled and lactifers tube present *Calotropis procera*

Rays >10 cells in width and >1 mm height…………………….. *Tamarix aphylla*

19 Storied structure present……………………………………………. go to n. 20

Storied structure absent……………………………………………. go to n. 21

20 Fibres stories and parenchyma diffuse…………………..….. *Balanites aegyptiaca*

Axial parenchyma and vessel elements storied……………… *Salvadora persica*

21 Tyloses common, fibres exclusively non septate and vessels of two distinct diameter classes………………………………………………... *Ficus benghalensis*

Wood not as above………………………………………………… *Ficus religiosa*

22 Rays uniseriate……………………………………………… ANACARDIACEAE

Rays multiseriate……………………………………………………… go to n. 23

23 Storied structure present…………………………………………… go to n. 24

Storied structure absent……………………………... *Clerodendrum multiflorum*

24 Rays of two distinct sizes, larger rays >10 seriate, sheath cells present and fibres storied…………………………………………………. *Leptadenia pyrotecnica*

Wood not as above……………………………………………….. *Cordia wallichi*

Leptadenia pyrotecnica **(Forssk.) Decne**

2. Growth ring boundaries indistinct or absent
5. Wood diffuse-porous
7. Vessels in diagonal and/or radial pattern
9. Vessels exclusively solitaire (90% or more)
10. Vessels in radial multiples of 4 or more common
13. Simple perforation plates
22. Intervessel pits alternate
23. Shape of alternate pits polygonal
25. Small – 4-7 μm
29. Vestured pits
30. Vessel-ray pits with distinct borders; similar to intervessel pits in size and shape throughout the ray cell
62. Fibers with distinctively border pits
63. Fiber pits common in both radial and tangential walls
66. Non septate fibers present
69. Fibers thin- to thick-walled
78. Axial parenchyma scanty paratracheal
85. Axial parenchyma in bands more than 3 cells wide
99. Larger rays commonly <10-seriate
102. Ray height > 1 mm
103. Rays of two distinct sizes (uniseriate and multiseriate with more than 10 cells)
109. Rays with procumbent, upright and square cells mixed throughout the ray
110. Sheath cells
119. Low rays storied, high rays not storied
120. Axial parenchyma and/or vessel elements storied
121. Fibers storied
136. Prismatic crystals present
137. Prismatic crystals in upright and/or square cells

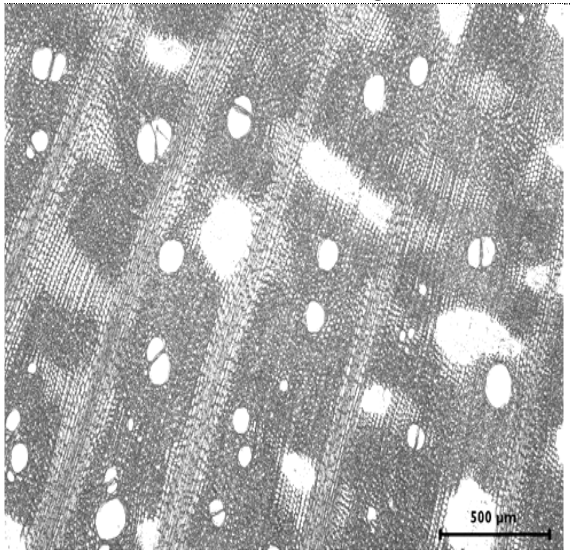
500 µm

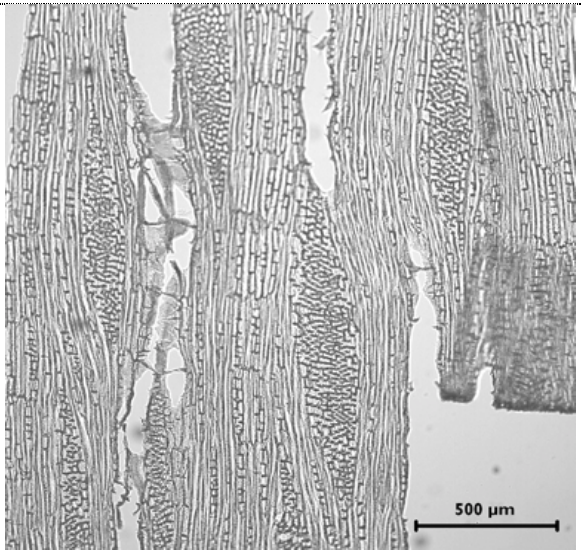
500 µm

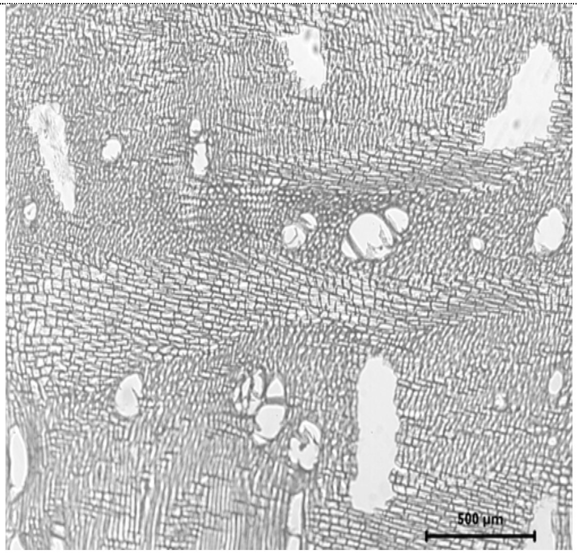
500 µm

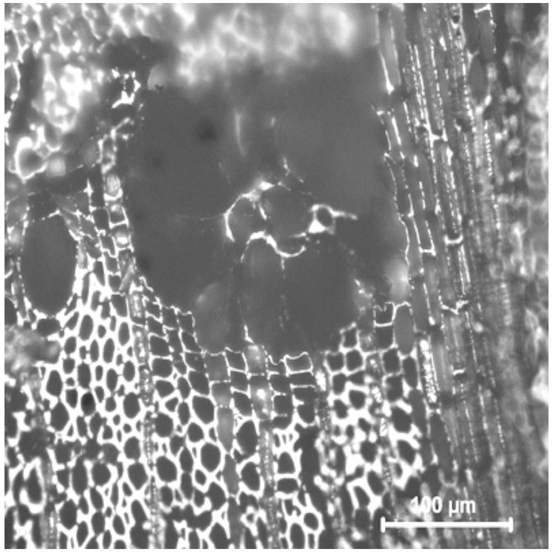
100 µm

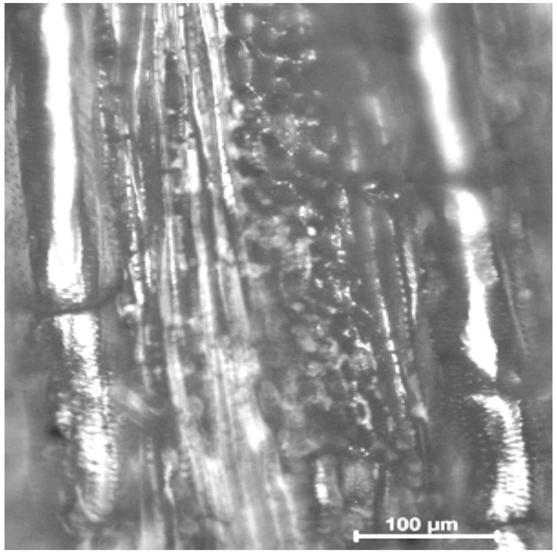
100 µm

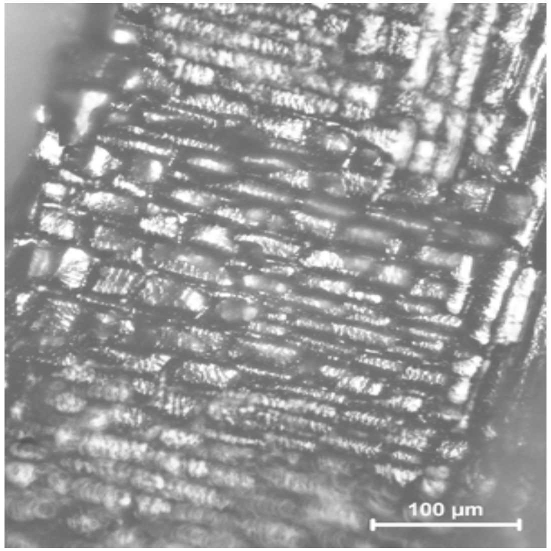
100 µm

ANACARDIACEAE

1. Growth ring boundaries distinct
2. Growth ring boundaries indistinct or absent
5. Wood diffuse-porous
7. Vessels in diagonal and/or radial pattern
10. Vessels in radial multiples of 4 or more common
13. Simple perforation plates
22. Intervessel pits alternate
23. Shape of alternate pits polygonal
26. Medium – 7-10 µm
27. Large - ≥ 10 µm
29. Vestured pits
79. Axial parenchyma vasicentric
83. Axial parenchyma confluent
84. Axial parenchyma unilateral paratracheal
85. Axial parenchyma in bands more than 3 cells wide
96. Rays exclusively uniseriate
109. Rays with procumbent, upright and square cells mixed throughout the ray
136. Prismatic crystals present
137. Prismatic crystals in upright and/or square cells

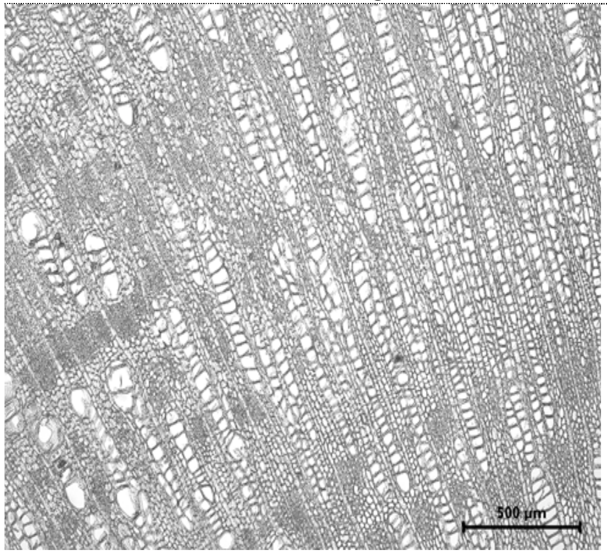
500 µm

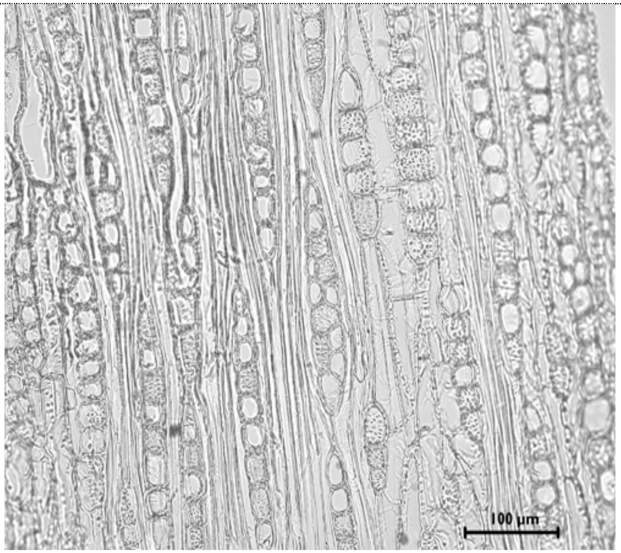
100 µm

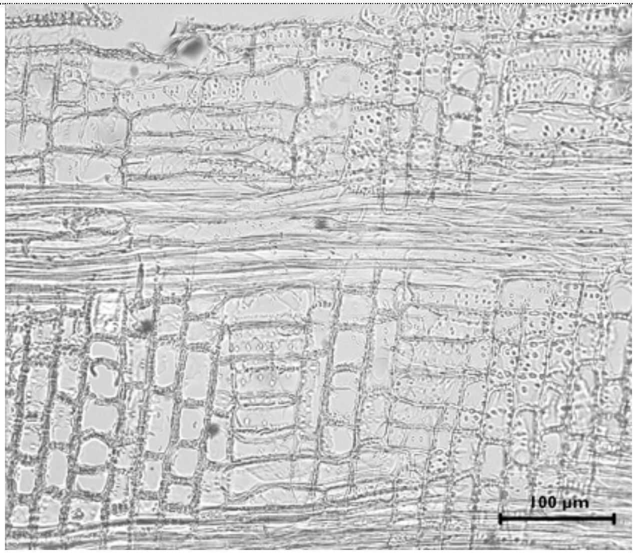
100 µm

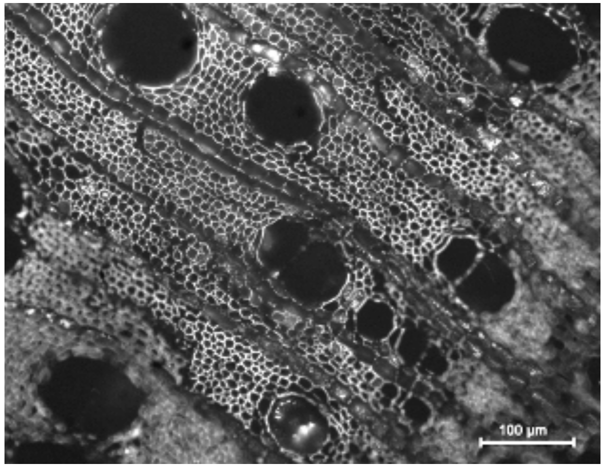
100 µm

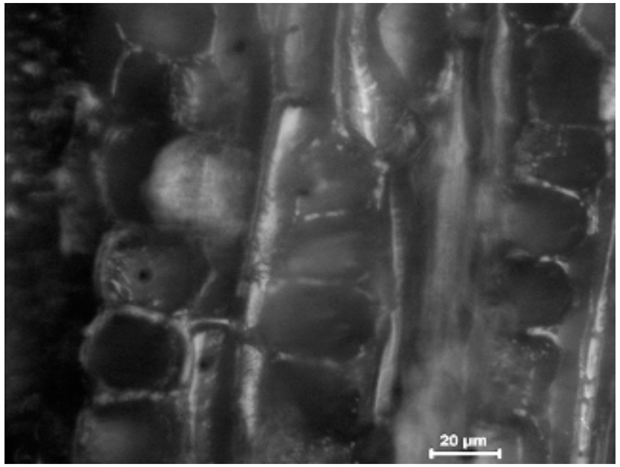
20 µm

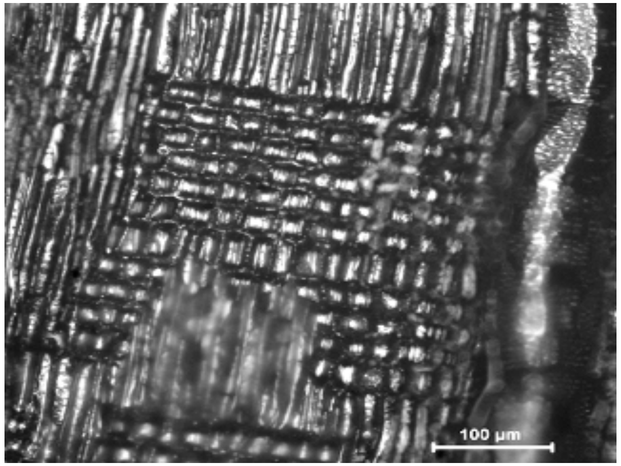
100 µm

***Calotropis procera* (Aiton) W.T. Aiton**

2. Growth ring boundaries indistinct or absent
5. Wood diffuse-porous
10. Vessels in radial multiples of 4 or more common
13. Simple perforation plates
22. Intervessel pits alternate
25. Small – 4-7 µm
26. Medium – 7-10 µm
29. Vestured pits
61. Fibers with simple to minutely bordered pits
66. Non septate fibers present
68. Fibers very thin-walled
76. Axial parenchyma diffuse
78. Axial parenchyma scanty paratracheal
91. Two cells per parenchyma strand
97. Ray width 1 to 3 cells
107. Body ray cells procumbent with mostly 2-4 rows of upright and/or square marginal cells
108. Body ray cells procumbent with over 4 rows of upright and/or square marginal cells
109. Rays with procumbent, square and upright cell mixed throughout the ray
132. Lactifers or tanniniferous tubes

S2 Wood and charcoal catalogue

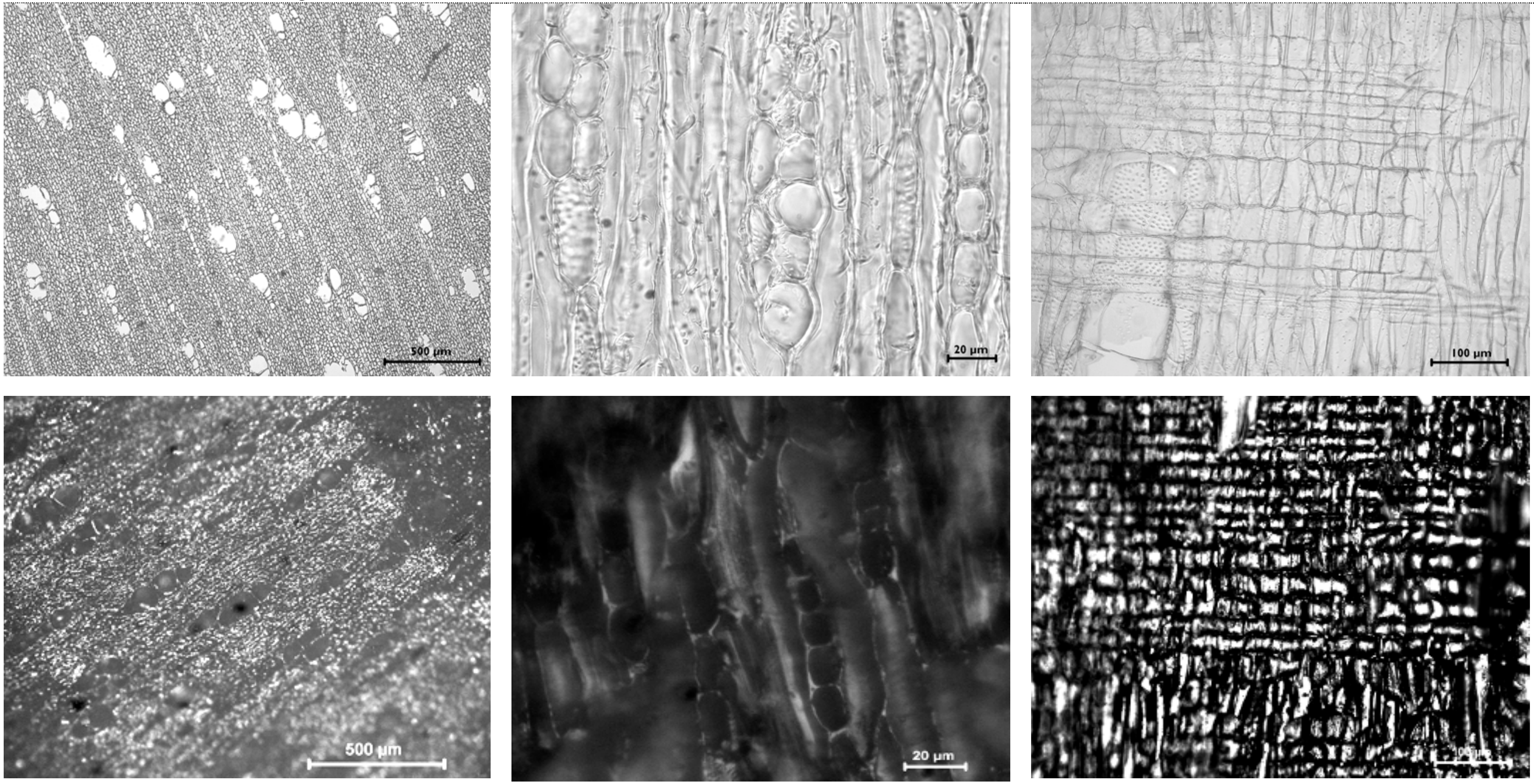

***Cordia* sp.**

2. Growth ring boundaries indistinct or absent
5. Wood diffuse-porous
10. Vessels in radial multiples of 4 or more common
13. Simple perforation plates
22. Intervessel pits alternate
45. Vessels of two distinct diameter class, wood not ring-porous
61. Fibers with simple to minutely bordered pits
66. Non septate fibers present
69. Fibers thin- to thick-walled
70. Fibers very thick-walled
78. Axial parenchyma scanty paratracheal
79. Axial parenchyma vasicentric
83. Axial parenchyma confluent
85. Axial parenchyma in bands more than 3 cells wide
86. Axial parenchyma in narrow bands or lines up to 3 cells wide
91. Two cells per parenchyma strands
92. Four (3-4) cells per parenchyma strand
97. Ray width 1 to 3 cells
98. Larger rays commonly 4 to 10-seriate
109. Rays with procumbent, upright and square cells mixed throughout the ray
120. Axial parenchyma and/or vessel elements storied
136. Prismatic crystals present
137. Prismatic crystals in upright and/or square cells
154. More than one crystal of about the same size per cell or chamber
155. Two distinct sizes of crystals per cell or chamber

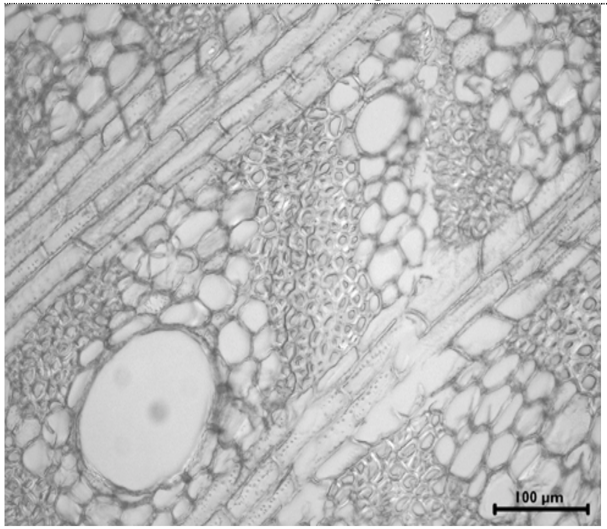
100 µm

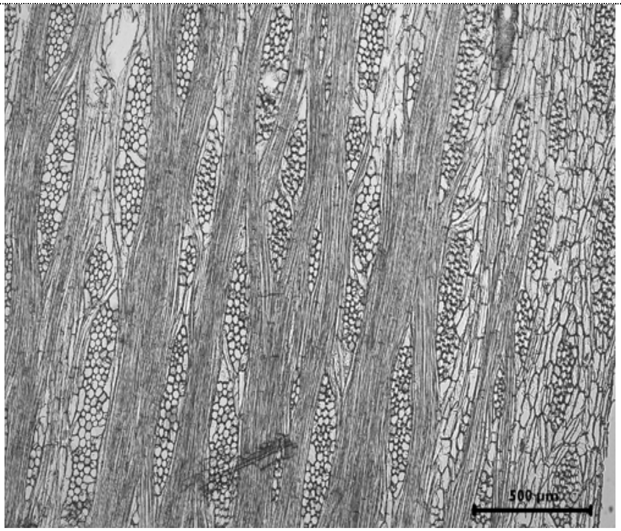
500 µm

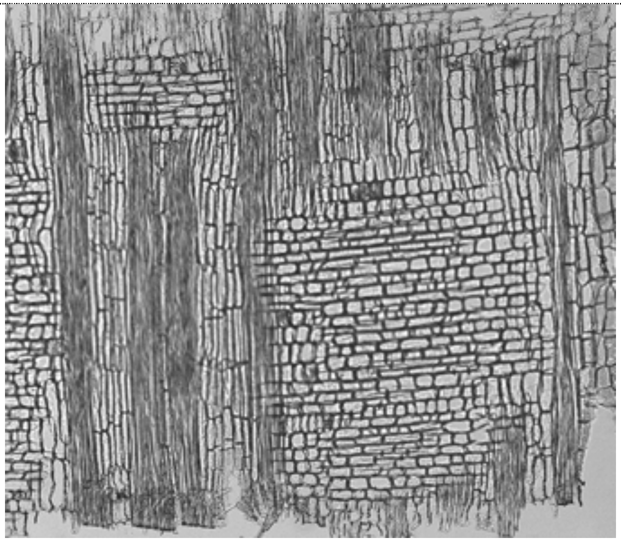

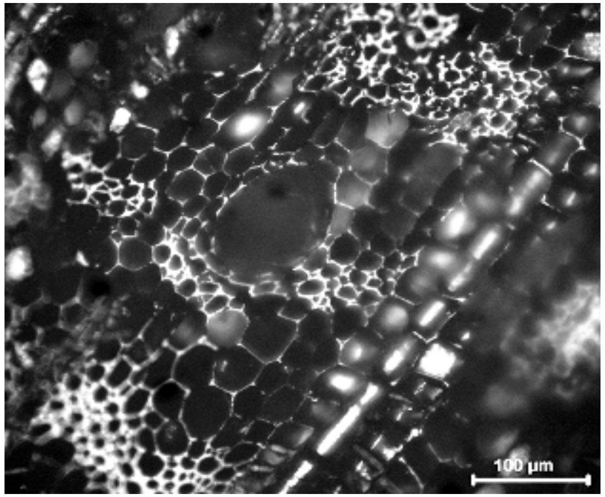
100 µm

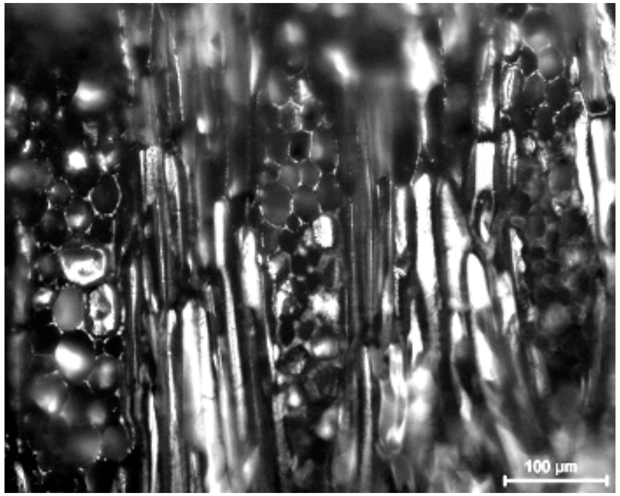
100 µm

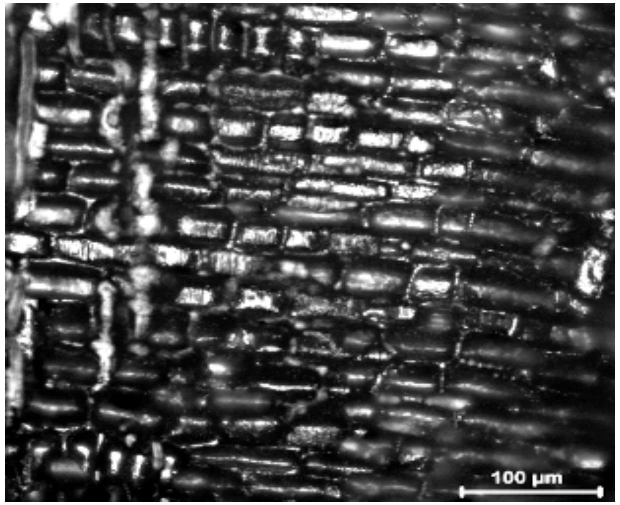
100 µm

***Capparis decidua* (Forssk.) Edgew**

2. Growth ring boundaries indistinct or absent

5. Wood diff use-porous

11. Vessels clusters common

13. Simple perforation plates

22. Intervessel pits alternate

24. Minute - ≤ 4 µm

29. Vestured pits

30. Vessel-ray pits with distinct borders; similar to intervessel pits in size and shape throughout the ray cell

45. Vessels of two distinct diameter class, wood not ring-porous

57. Tyloses sclerotic

61. Fibers with simple to minutely bordered pits

66. Non septate fibers present

69. Fibers thin- to thick-walled

79. Axial parenchyma vasicentric

91. Two cells per parenchyma strands

93. Eight (5-8) cells per parenchyma strand

97. Ray width 1 to 3 cells

98. Larger rays commonly 4 to 10-seriate

103. Rays of two distinct sizes

104. All ray cells procumbent

106. Body ray cell procumbent with one row of upright and/or square marginal cells

136. Prismatic crystals present

(138. Prismatic crystals in procumbent ray cells)

S2: Wood and charcoal catalogue

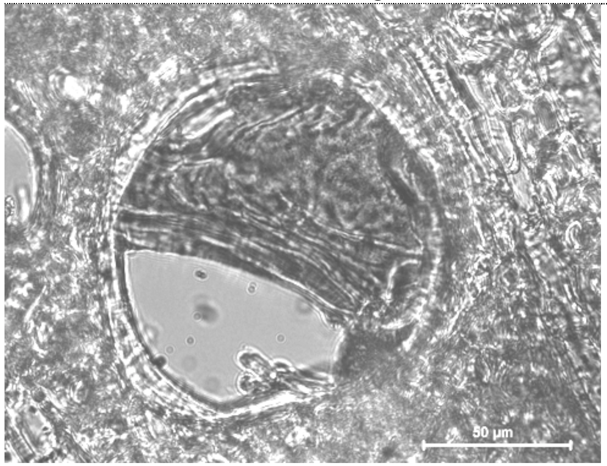

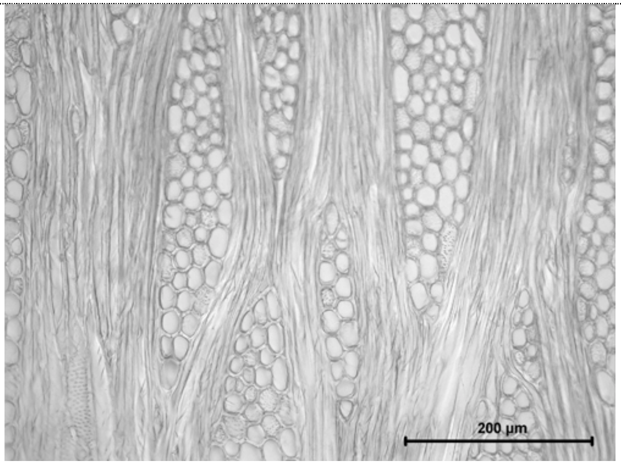

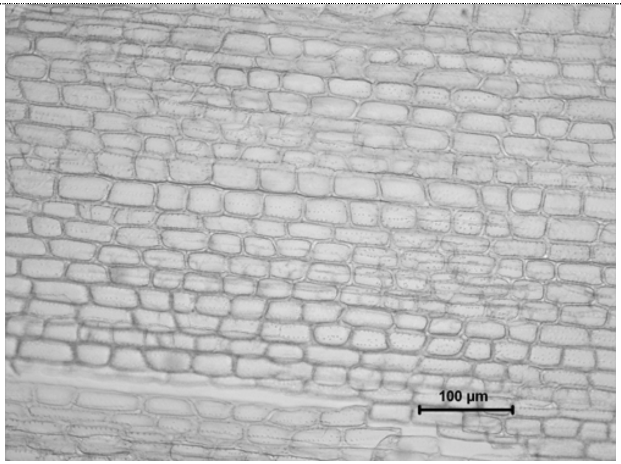

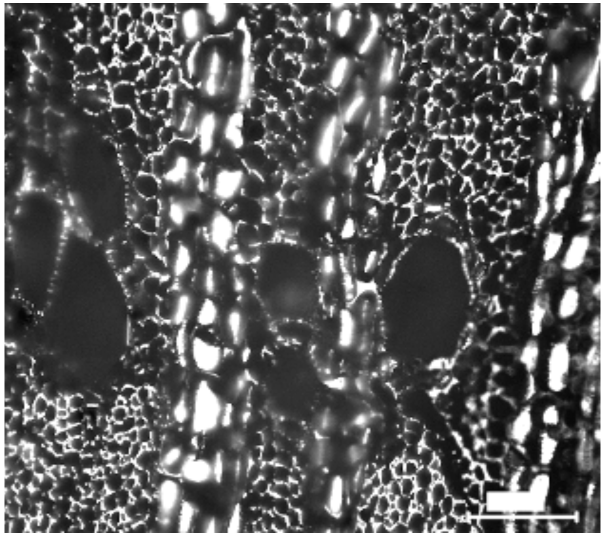

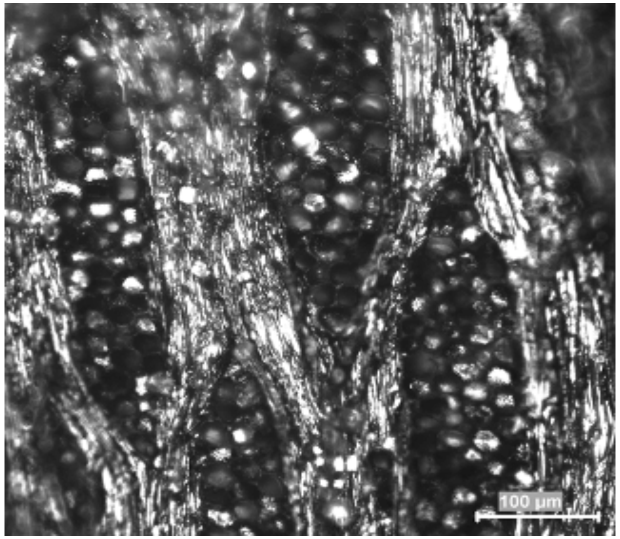

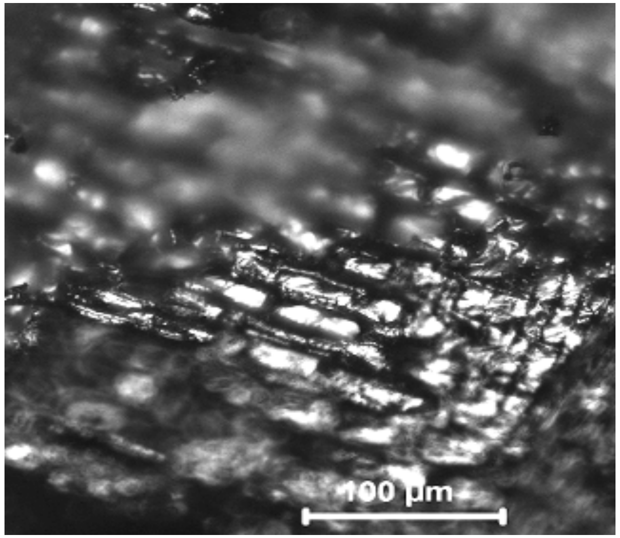

***Suaeda monoica* Forssk. Ex J.F.Gmel**

2. Growth ring boundaries indistinct or absent

5. Wood diffuse-porous

6. Vessels in tangential bands (festooned)

11. Vessel clusters common

13. Simple perforation plates

45. Vessels of two distinct diameter class, wood not ring-porous

(60. Vascular/vasicentric sclereids present)

(61. Fibers with simple to minutely bordered pits)

69. Fibers thin- to thick-walled

78. Axial parenchyma scanty paratracheal

84. Axial parenchyma unilateral paratracheal

91. Two cells per parenchyma strands

92. Four (3-4) cells per parenchyma strand

117. Wood rayless

120. Axial parenchyma and/or vessel elements storied

136. Prismatic crystals present

141. Crystals in non chambered axial parenchyma cells

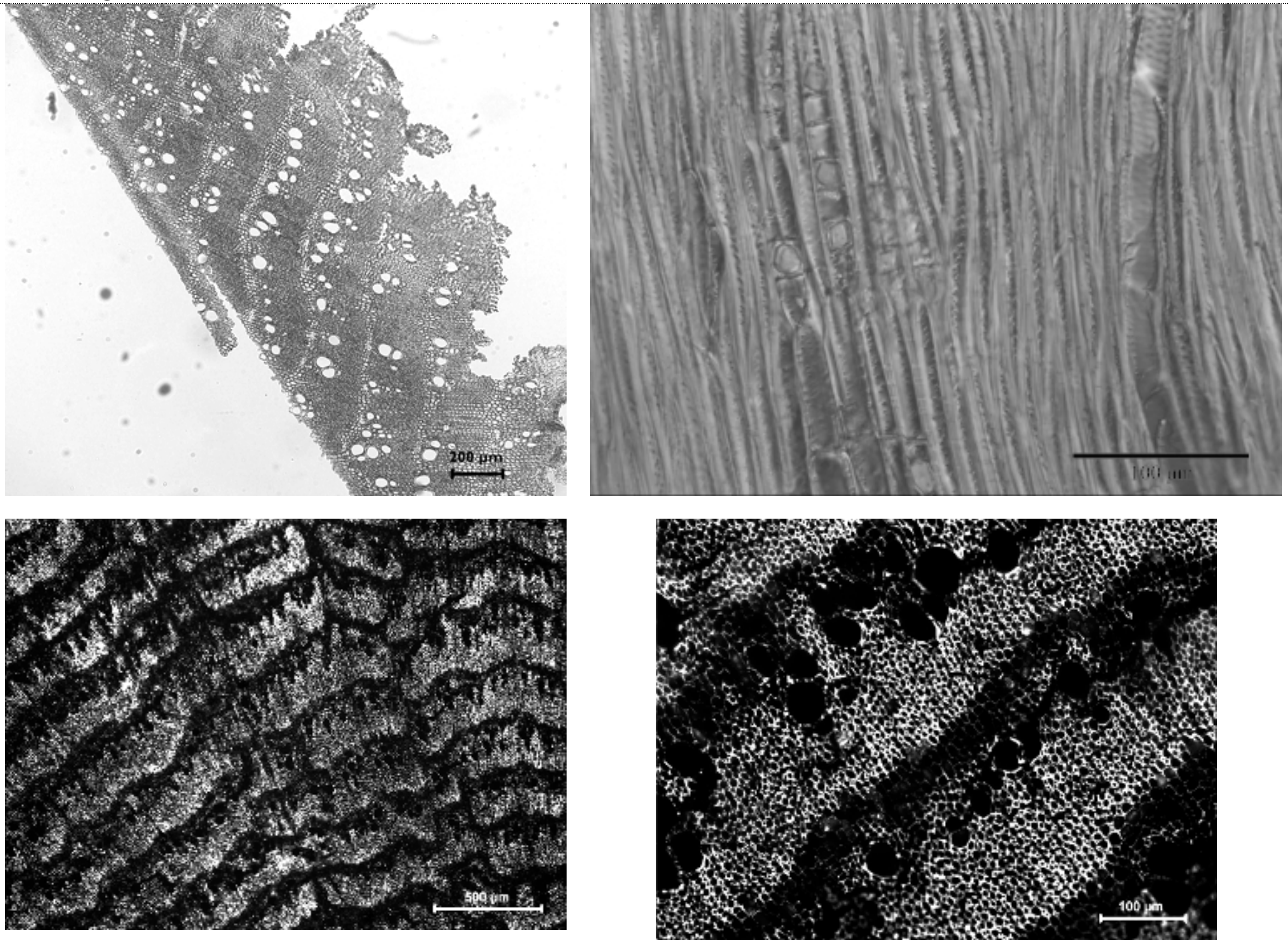
200 µm
100 µm
500 µm
100 µm

Acacia farnesiana **(L.) Willd.**

2. Growth ring boundaries indistinct or absent
5. Wood diffuse-porous
11. Vessel cluster common
13. Simple perforation plates
22. Intervessel pits alternate
23. Shape of alternate pits polygonal
26. Medium – 7-10 µm
45. Vessels of two distinct diameter class, wood not ring-porous
56. Tyloses common
70. Fibers very thick-walled
79. Axial parenchyma vasicentric
83. Axial parenchyma confluent
85. Axial parenchyma in bands more than 3 cells wide
92. Four (3-4) cells per parenchyma strand
98. Larger rays commonly 4 to 10-seriate
103. Rays of two distinct sizes
104. All ray cells procumbent
136. Prismatic crystals present
141. Prismatic crystals in non chambered axial parenchyma cells
142. Prismatic crystals in chambered axial parenchyma cells

S2: Wood and charcoal catalogue

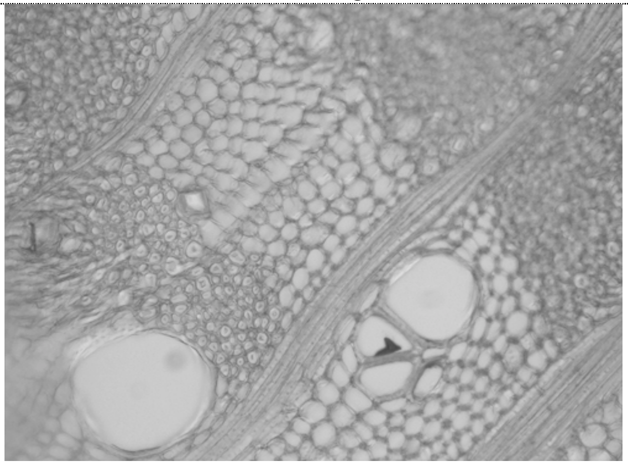

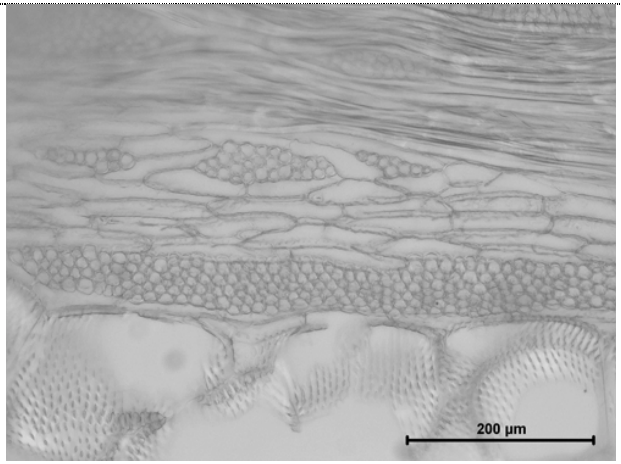

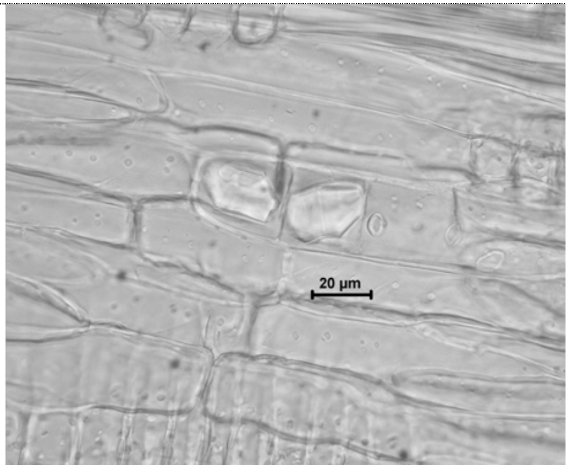

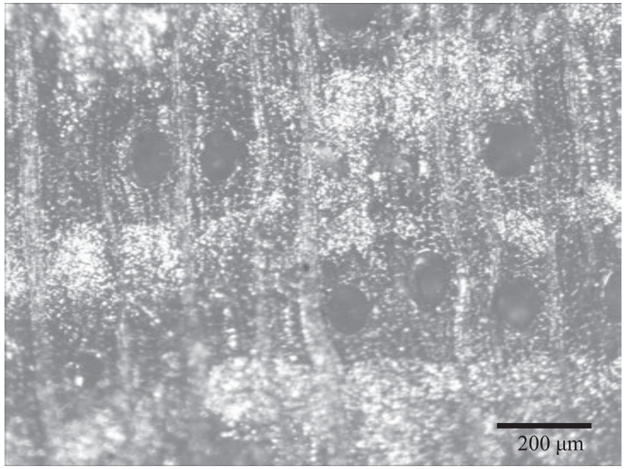

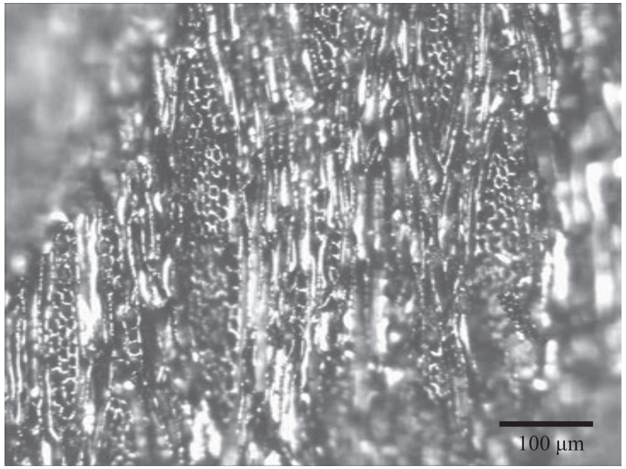

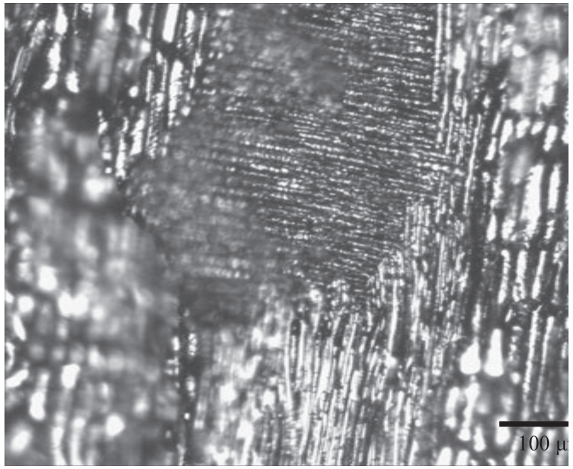

***Acacia nilotica* (L) Delile**

2. Growth ring boundaries indistinct or absent
5. Wood diffuse-porous
13. Simple perforation plates
22. Intervessel pits alternate
23. Shape of alternate pits polygonal
29. Vestured pits
61. Fibres with simple to minutely bordered pits
66. Non-septate fibres present
69. Fibres thin- to thick-walled
79. Axial parenchyma vasicentric
98. Larger rays commonly 4 - to 10 seriate
104. All ray cells procumbent

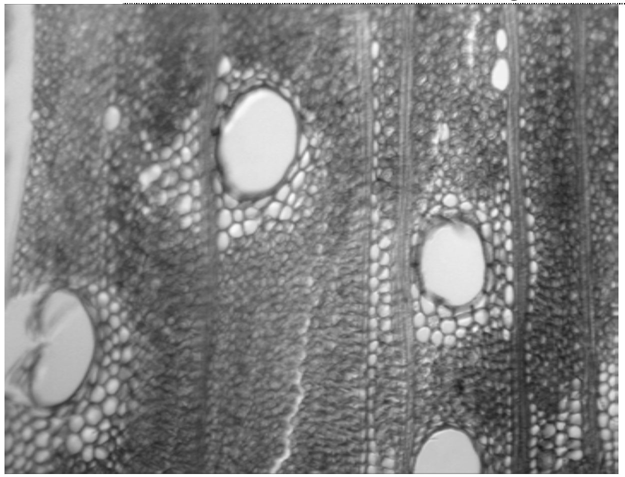

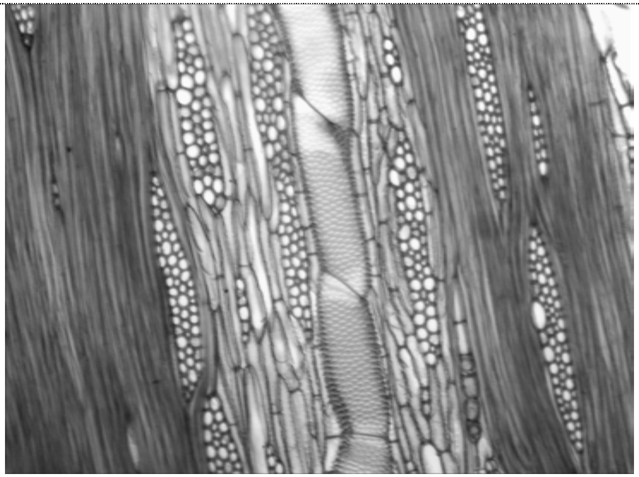

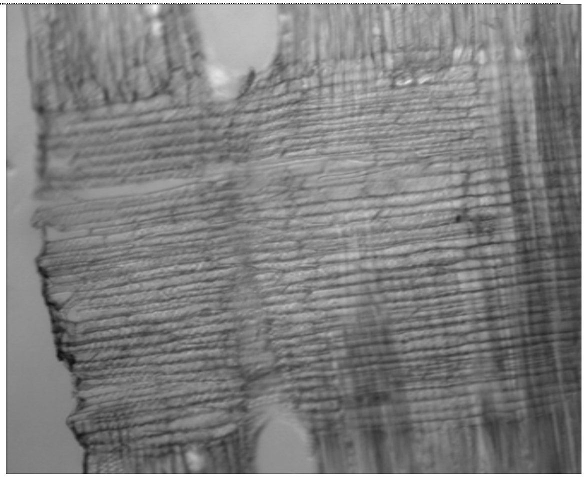

***Acacia cf. senegal* (L.) Willd.**

2. Growth ring boundaries indistinct or absent

5. Wood diffuse-porous

7. Vessels in diagonal and/or radial pattern

13. Simple perforation plates

22. Intervessel pits alternate

23. Shape of alternate pits polygonal

24. Minute - ≤ 4 µm

26. Medium – 7-10 µm

29. Vestured pits

30. Vessel-ray pits with distinct borders; similar to intervessel pits in size and shape throughout the ray cell

45. Vessels of two distinct diameter class, wood not ring-porous

69. Fibers thin- to thick-walled

80. Axial parenchyma aliform

81. Axial parenchyma lozenghe-aliform

83. Axial parenchyma confluent

97. Ray width 1 to 3 cells

98. Larger rays commonly 4 to 10-seriate

103. Rays of two distinct sizes

104. All ray cells procumbent

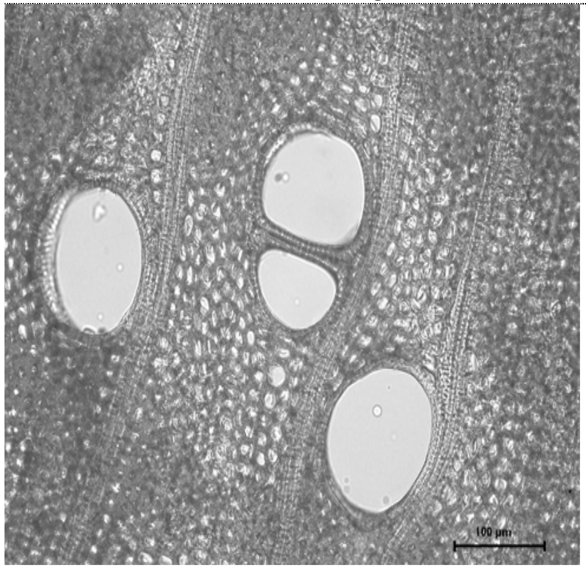
100 µm

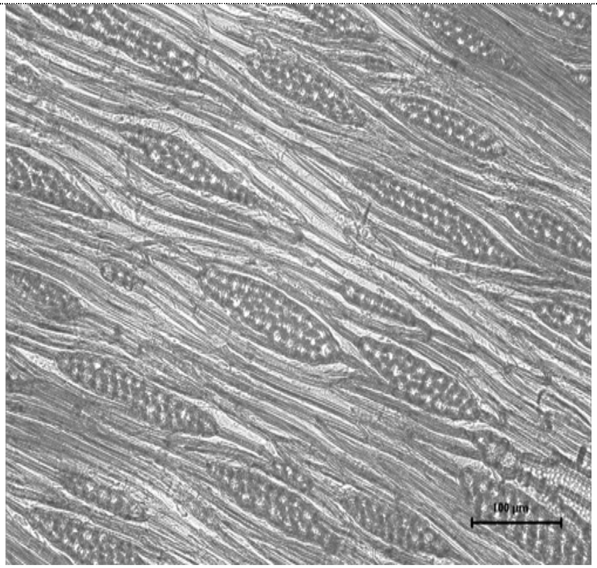
100 µm

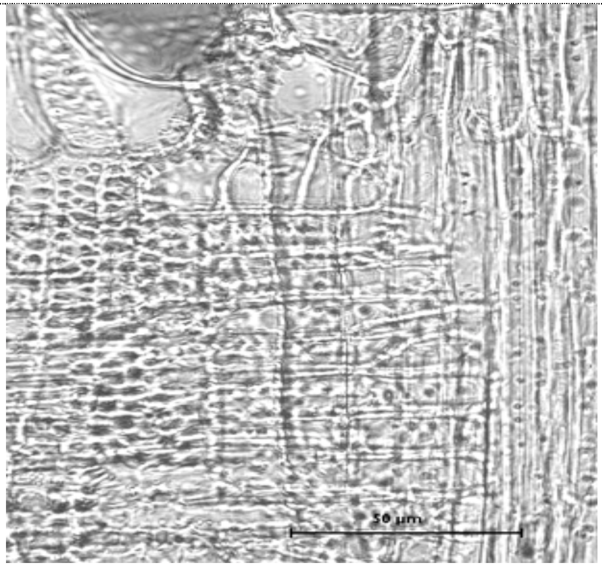
50 µm

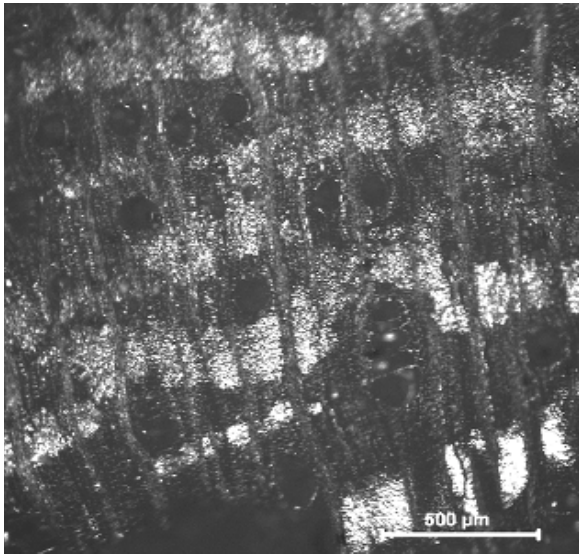
500 µm

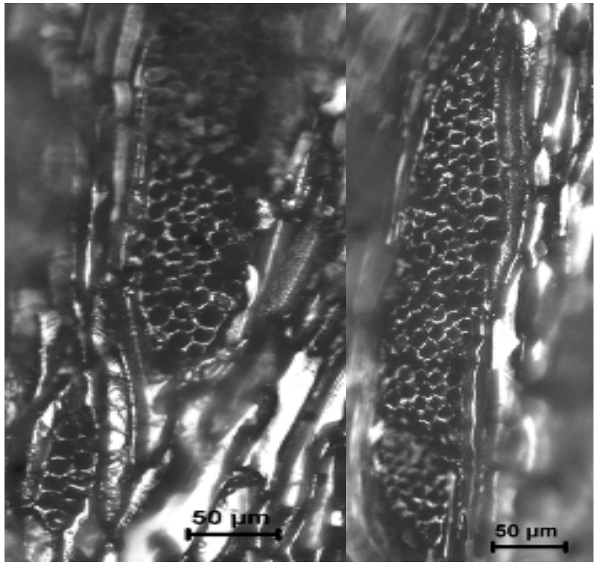
50 µm
50 µm

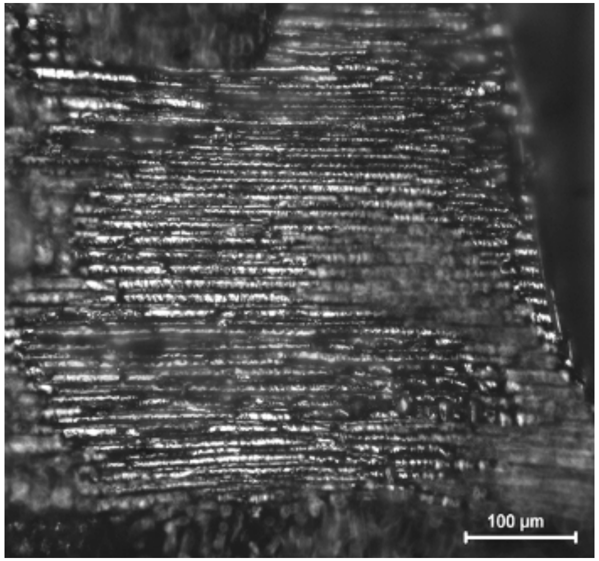
100 µm

***Leucaena leucocephala* (Lam.) de Wit**

1. Growth ring boundaries distinct

5. Wood diffuse-porous

10. Vessels in radial multiples of 4 or more common

13. Simple perforation plates

22. Intervessel pits alternate

27. Large - ≥ 10 µm

30. Vessel-ray pits with distinct borders; similar to intervessel pits in size and shape throughout the ray cell

45. Vessels of two distinct diameter class, wood not ring-porous

65. Septate fibers present

66. Non septate fibers present

68. Fibers very thin-walled

79. Axial parenchyma vasicentric

83. Axial parenchyma confluent

92. Four (3-4) cells per parenchyma strand

97. Rays width 1 to 3 cells

104. All ray cells procumbent

136. Prismatic crystals present

143. Prismatic crystals in fibers

S2: Wood and charcoal catalogue

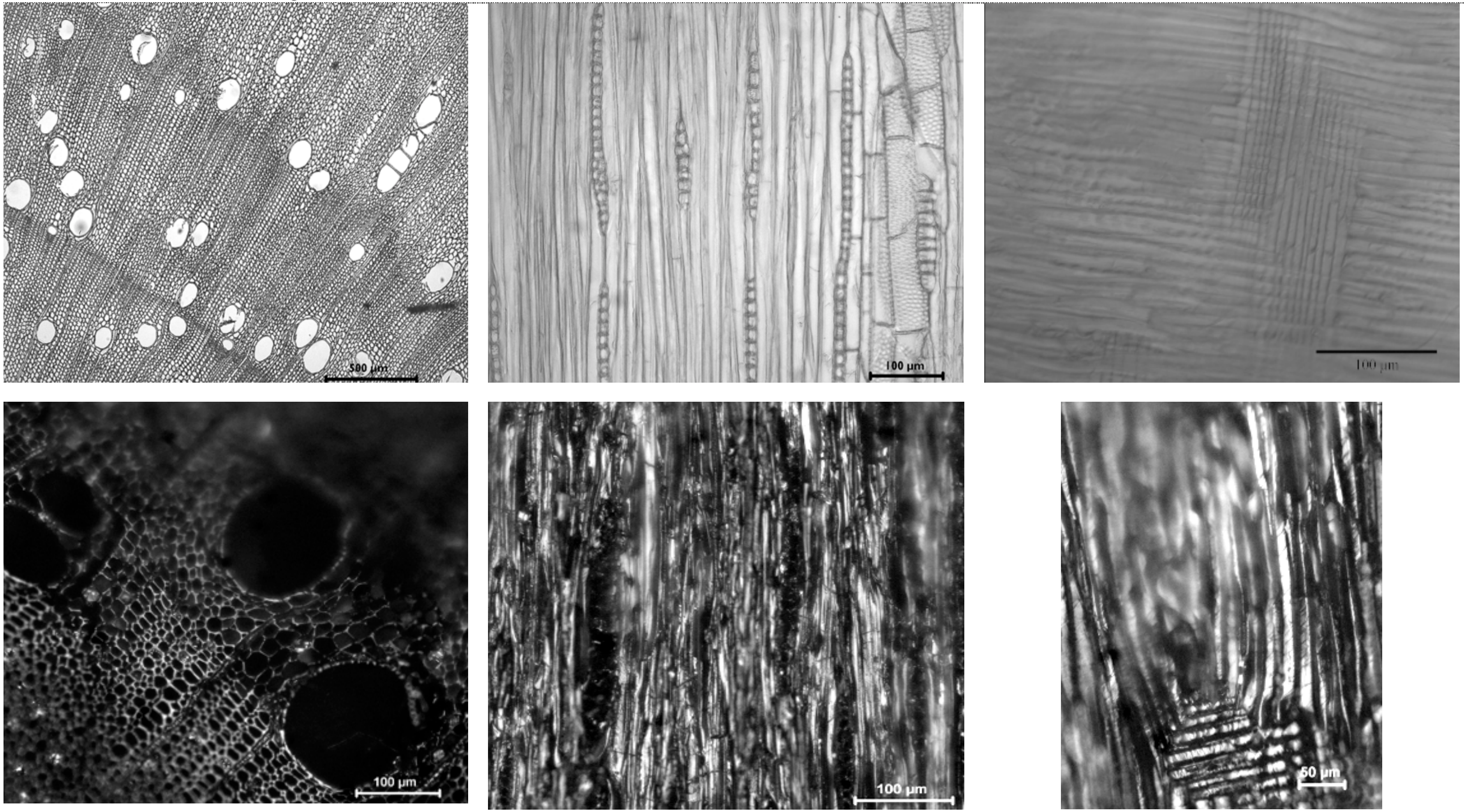

***Prosopis cineraria* (L.) Druce**

1. Growth ring boundaries distinct

3. Wood ring-porous

10. Vessels in radial multiples of 4 or more common

11. Vessels clusters common

13. Simple perforation plates

22. Intervessel pits alternate

23. Shape of alternate pits polygonal

25. Small – 4-7 µm

26. Medium – 7-10 µm

29. Vestured pits

30. Vessel-ray pits with distinct borders; similar to intervessel pits in size and shape throughout the ray cell

45. Vessels of two distinct diameter class, wood not ring-porous

61. Fibers with simple to minutely bordered pits

66. Non septate fibers present

69. Fibers thin- to thick-walled

79. Axial parenchyma vasicentric

81. Axial parenchyma lozenghe-aliform

83. Axial parenchyma confluent

85. Axial parenchyma in bands more than 3 cells wide

92. Four (3-4) cells per parenchyma strand

97. Ray width 1 to 3 cells

104. All ray cells procumbent

136. Prismatic crystals present

143. Prismatic crystals in fibres

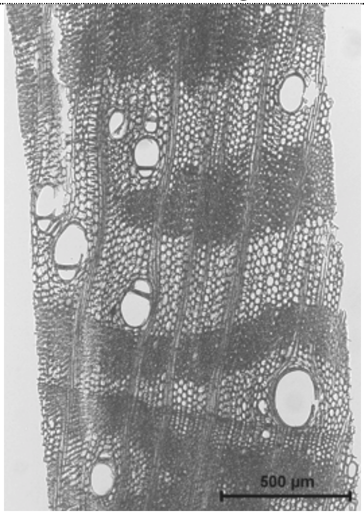
500 µm

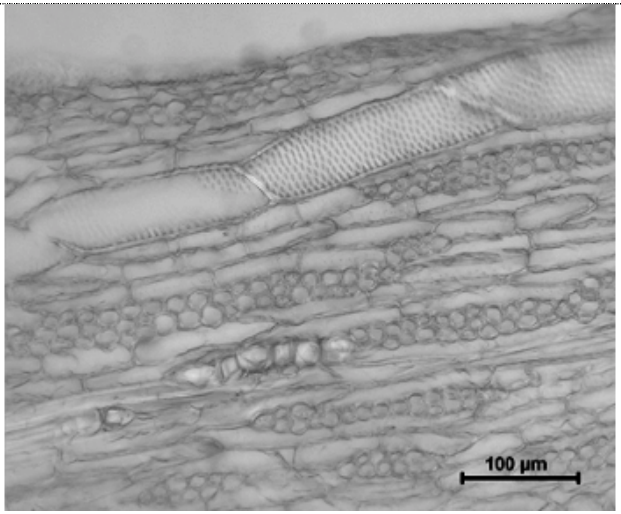
100 µm

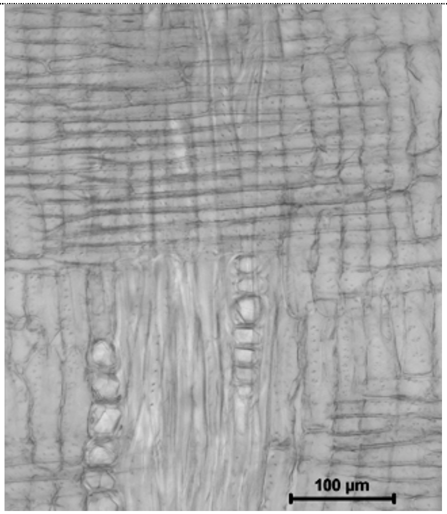
100 µm

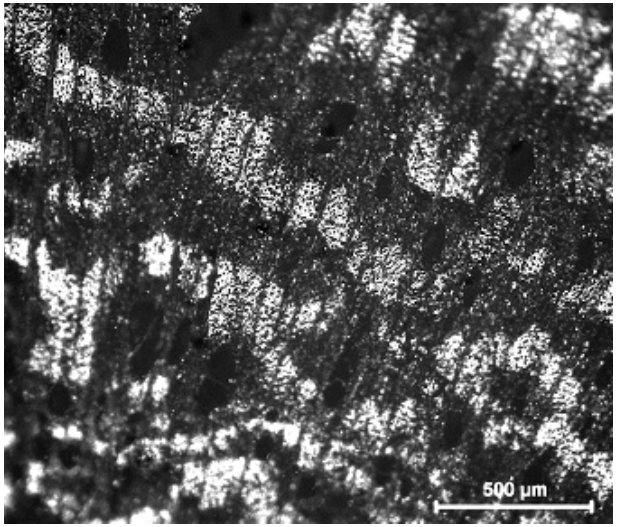
500 µm

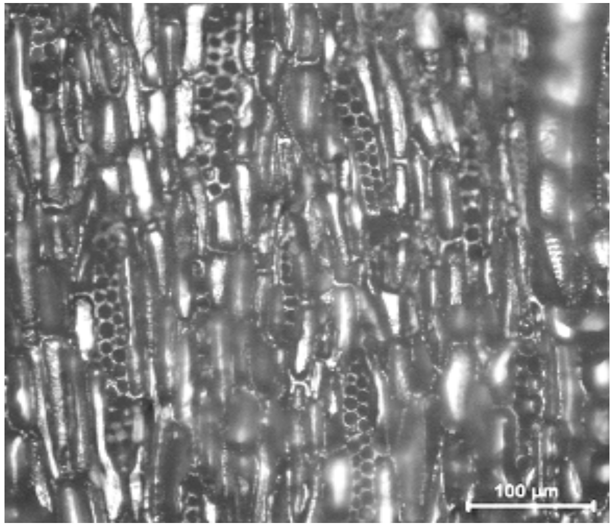
100 µm

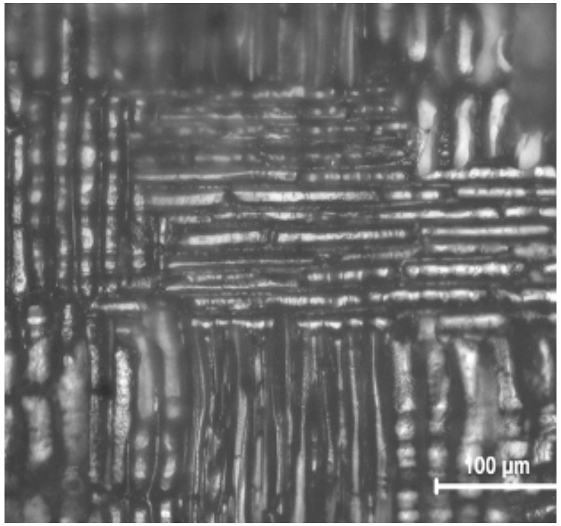
100 µm

Prosopis juliflora **(Sw.) DC.**

1. Growth ring boundaries distinct

2. Growth ring boundaries indistinct or absent

5. Wood diffuse-porous

13. Simple perforation plates

22. Intervessel pits alternate

23. Shape of alternate pits polygonal

25. Small – 4-7 μm

26. Medium – 7-10 μm

29. Vestured pits

30. Vessel-ray pits with distinct borders; similar to intervessel pits in size and shape throughout the ray cell

(36v. Helical thickenings in vessel elements present)

61. Fibers with simple to minutely bordered pits

70. Fibers very thick-walled

71. Mean fibers length ≤ 900 μm

79. Axial parenchyma vasicentric

80. Axial parenchyma aliform

81. Axial parenchyma lozenghe-aliform

83. Axial parenchyma confluent

136. Prismatic crystals present

142. Prismatic crystals in chambered axial parenchyma cells

S2: Wood and charcoal catalogue

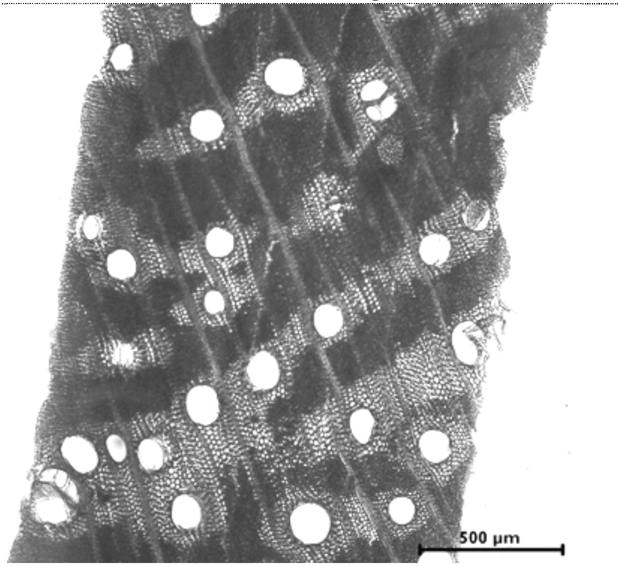

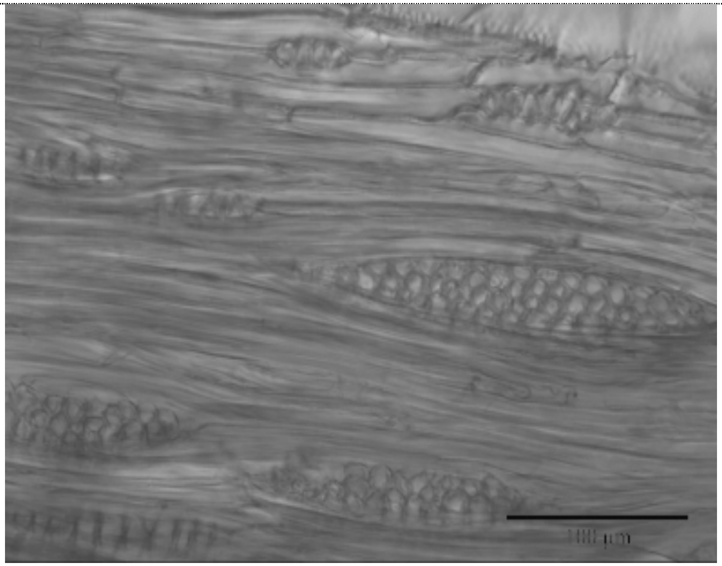

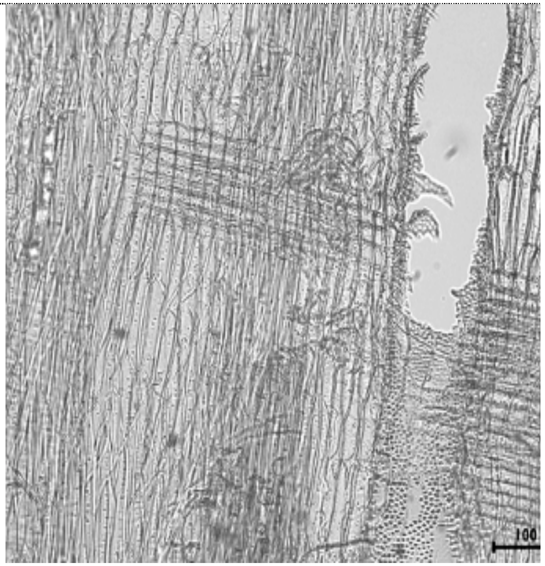

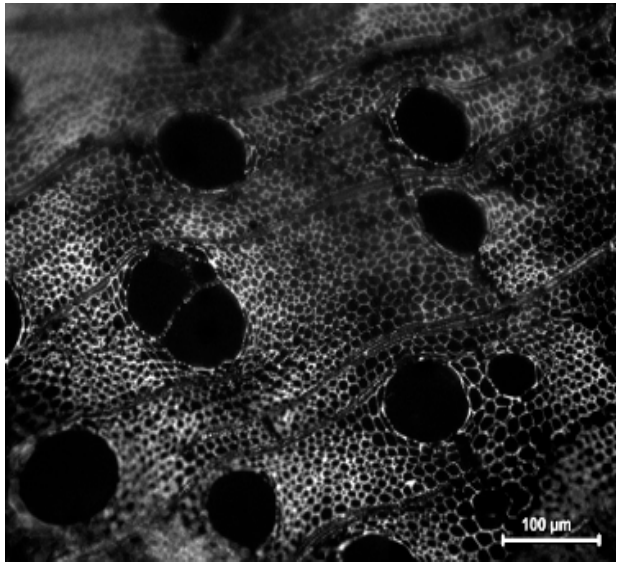

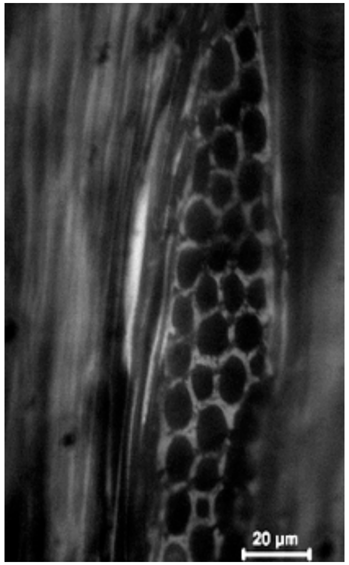

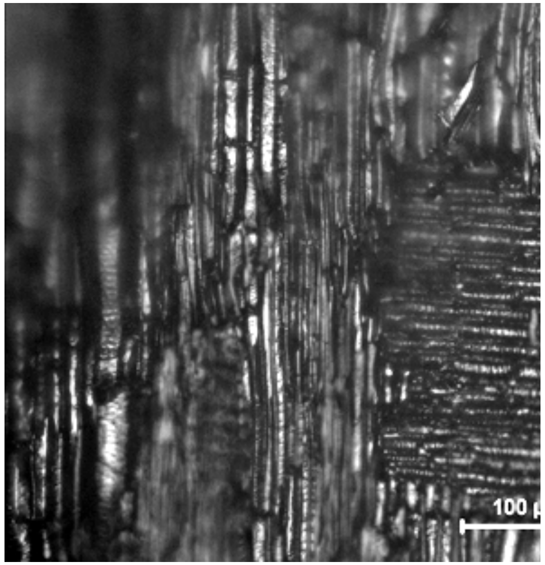

***Senna auriculata* (L.) Roxb.**

2. Growth ring boundaries indistinct or absent

5. Wood diffuse-porous

7. Vessels in diagonal and/or radial pattern

10. Vessels in radial multiples of 4 or more common

13. Simple perforation plates

22. Intervessel pits alternate

29. Vestured pits

30. Vessel-ray pits with distinct borders; similar to intervessel pits in size and shape throughout the ray cell

61. Fibers with simple to minutely bordered pits

66. Non septate fibers present

70. Fibers very thick-walled

79. Axial parenchyma vasicentric

80. Axial parenchyma aliform

83. Axial parenchyma confluent

85. Axial parenchyma in bands more than 3 cells wide

91. Two cells per parenchyma strands

92. Four (3-4) cells per parenchyma strand

97. Ray width 1 to 3 cells

104. All ray cells procumbent

136. Prismatic crystals present

142. Prismatic crystals in chambered axial parenchyma cells

Differences between Senna siamea and Senna auriculata:

- much more regularly banded and vasicentric parenchyma almost absent in Senna siamea

- crystals more abundant in Senna siamea

- RLS more regular and procumbent cells thinner in Senna siamea than in Senna auriculata

S2: Wood and charcoal catalogue

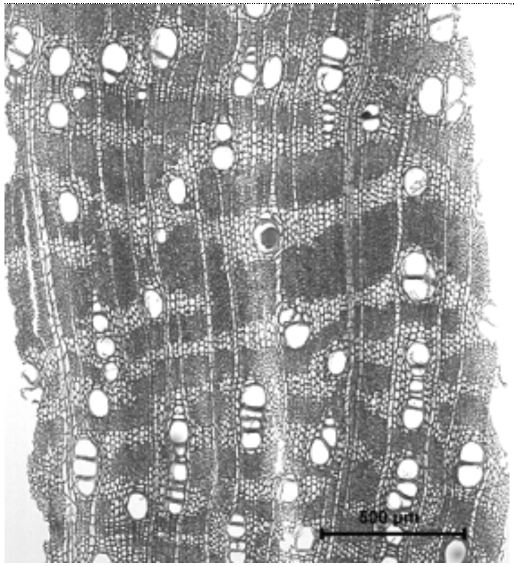

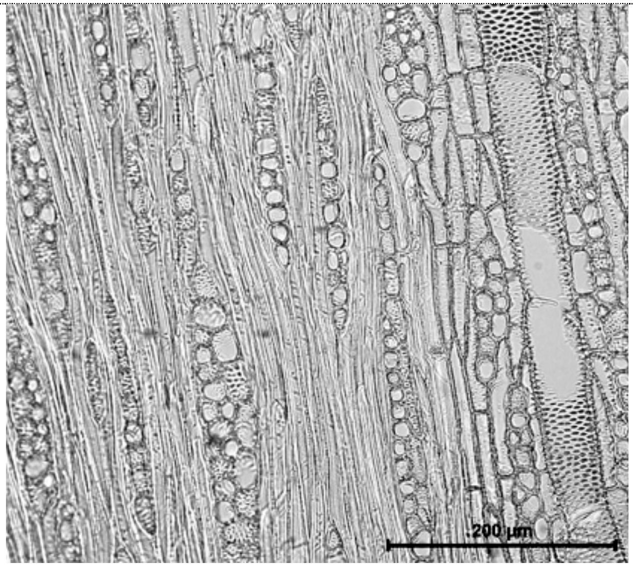

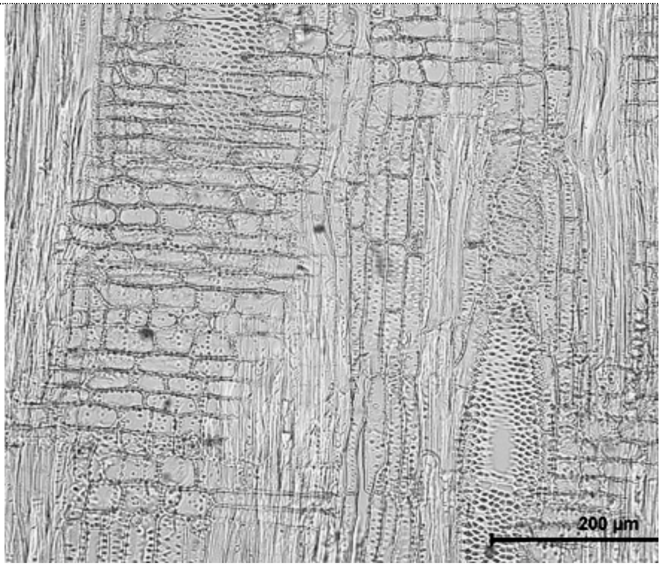

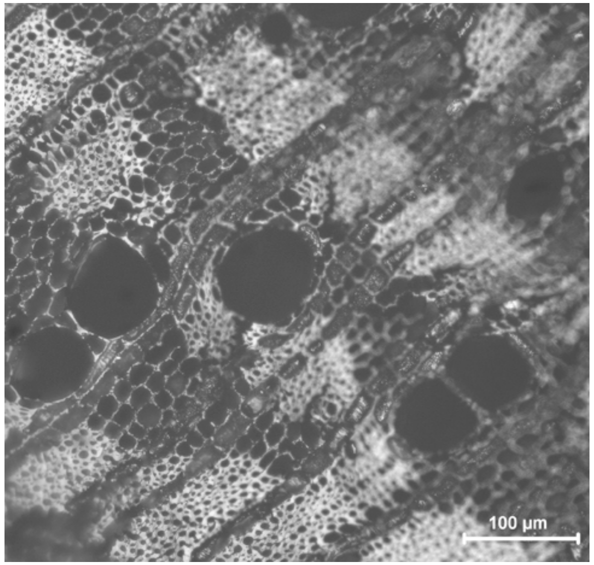

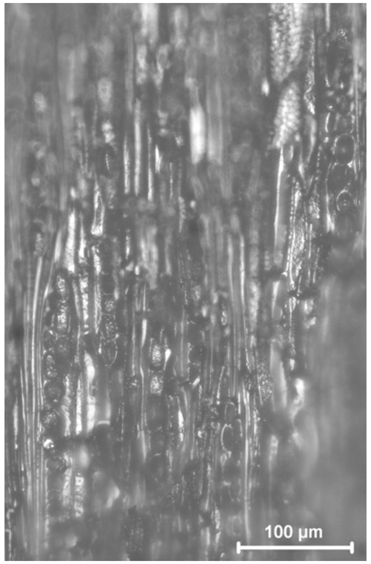

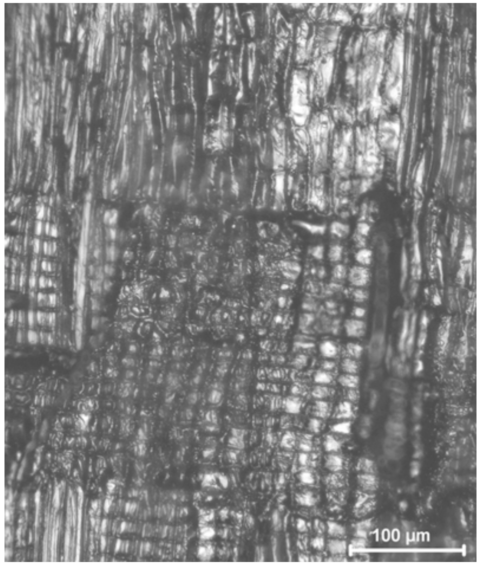

***Senna siamea* (Lam.) H.S. Irwin & Barneby**

2. Growth ring boundaries indistinct or absent

5. Wood diffuse-porous

7. Vessels in diagonal and/or radial pattern

10. Vessels in radial multiples of 4 or more common

13. Simple perforation plates

22. Intervessel pits alternate

23. Shape of alternate pits polygonal

26. Medium – 7-10 μm

27. Large - ≥ 10 μm

29. Vestured pits

45. Vessels of two distinct diameter class, wood not ring-porous

61. Fibers with simple to minutely bordered pits

66. Non-septate fibers present

70. Fibers very thick-walled

85. Axial parenchyma in bands more than 3 cells wide

88. Axial parenchyma scalariform

91. Two cells per parenchyma strands

92. Four (3-4) cells per parenchyma strand

97. Ray width 1 to 3 cells

104. All ray cells procumbent

136. Prismatic crystals present

142. Prismatic crystals in chambered axial parenchyma cells

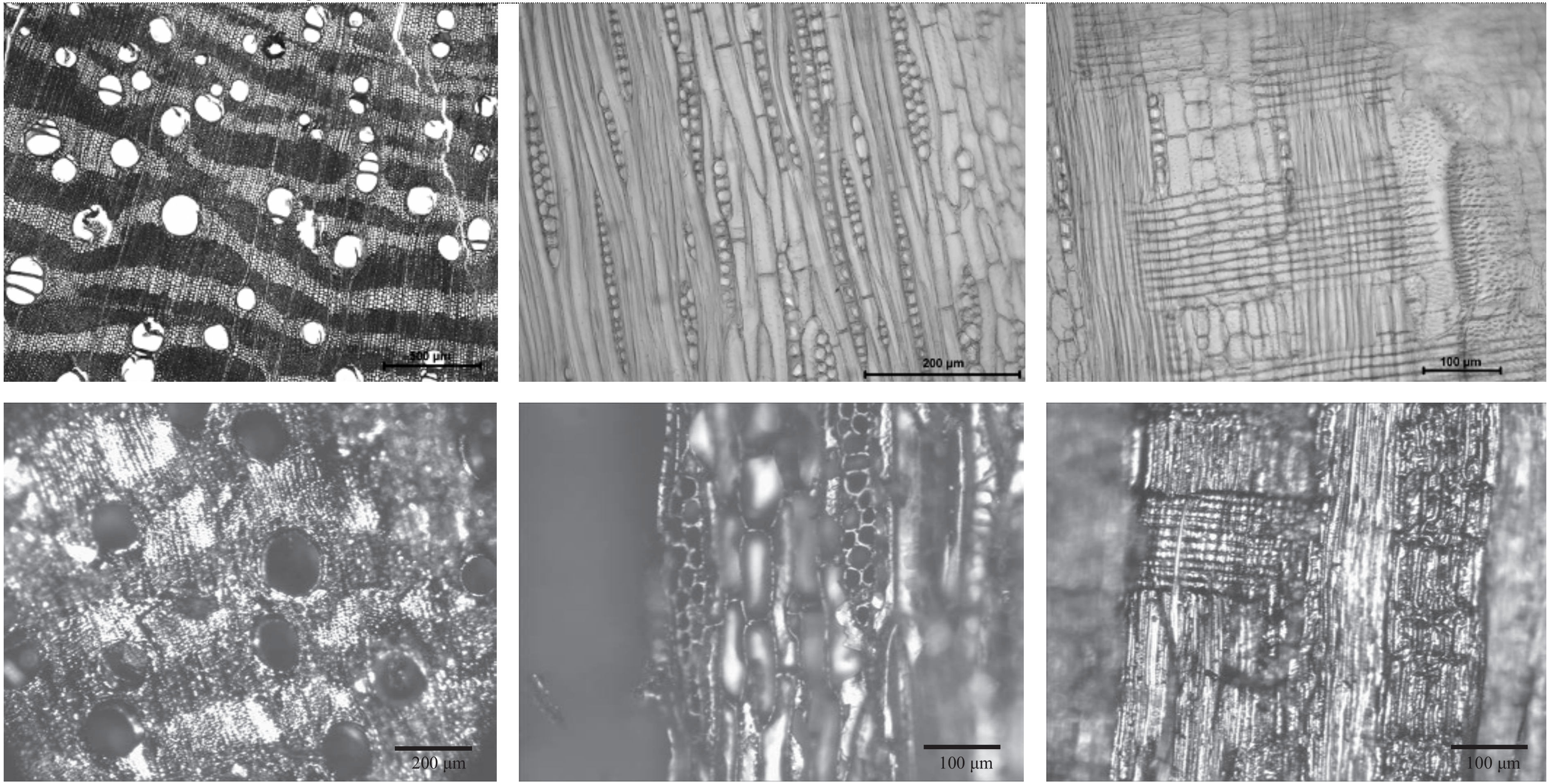
500 µm
200 µm
100 µm
200 µm
100 µm
100 µm

Clerodendrum sp.

1. Growth ring boundaries distinct
4. Wood semi-ring-porous
5. Wood diffuse-porous
6. Vessels in tangential bands
10. Vessels in radial multiples of 4 or more common
13. Simple perforation plates
22. Intervessel pits alternate
23. Shape of alternate pits polygonal
24. Minute - ≤ 4 µm
29. Vestured pits
30. Vessel-ray pits with distinct borders; similar to intervessel pits in size and shape throughout the ray cell
56. Tyloses common
61. Fibers with simple to minutely bordered pits
65. Septate fibers present
68. Fibers very thin-walled
75. Axial parenchyma absent or extremely rare
97. Ray width 1 to 3 cells
98. Larger rays commonly 4 to 10-seriate
109. Rays with procumbent, upright and square cells mixed throughout the ray
136. Prismatic crystals present
154. More than one crystal of about the same size per cell or chamber
155. Two distinct sizes of crystals per cell or chamber
157. Crystals in tylose

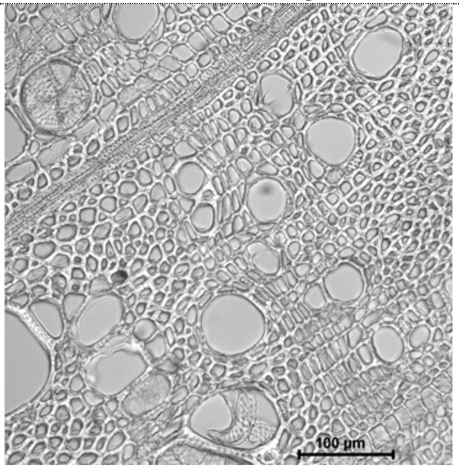
100 µm

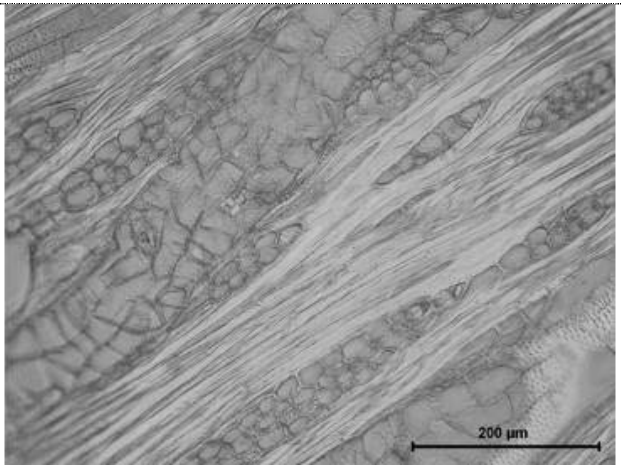
200 µm

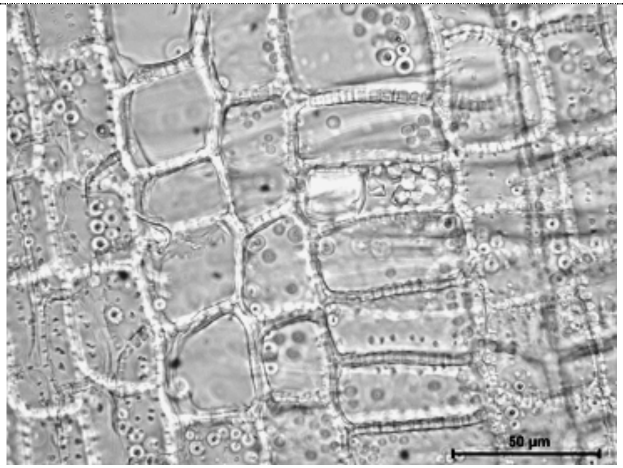
50 µm

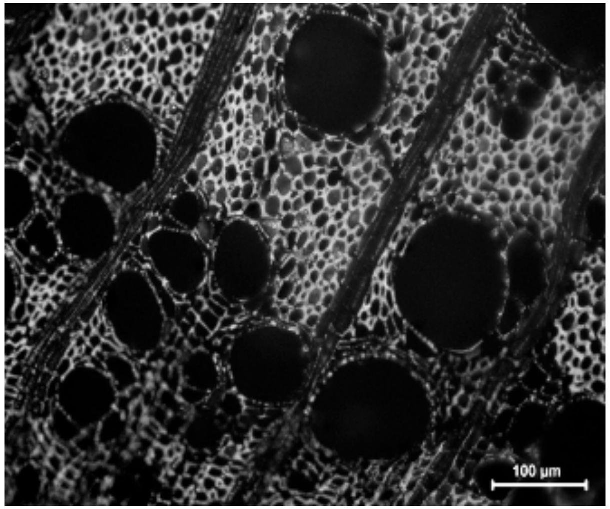
100 µm

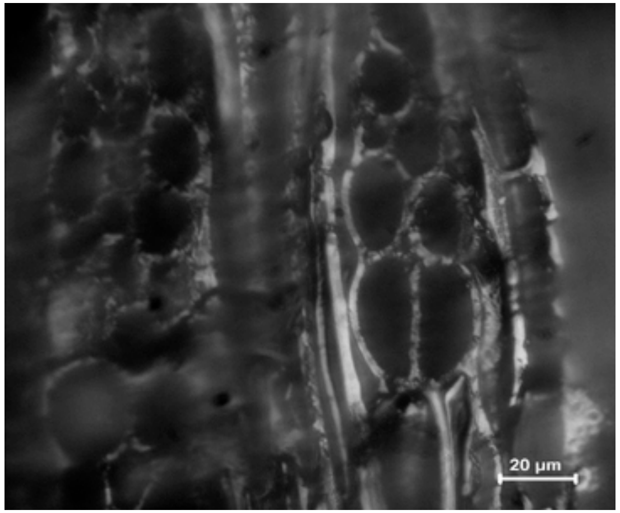
20 µm

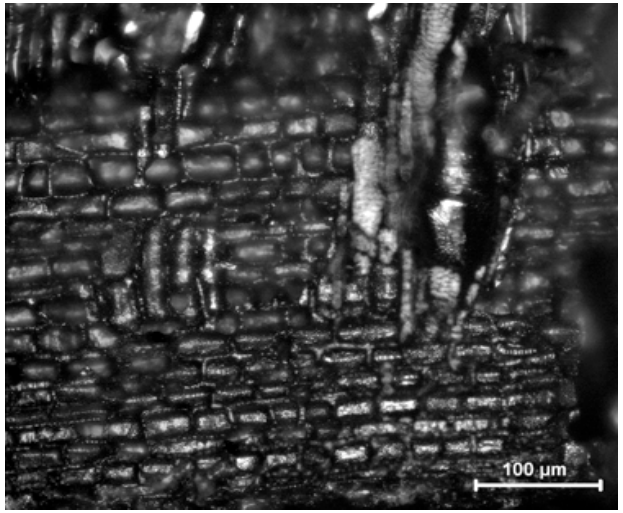
100 µm

Azadirachta indica **A. Juss.**

2. Growth ring boundaries indistinct or absent

5. Wood diffuse-porous

6. Vessels in tangential bands

11. Vessels clusters common

13. Simple perforation plates

22. Intervessel pits alternate

25. Small – 4-7 µm

30. Vessel-ray pits with distinct borders; similar to intervessel pits in size and shape throughout the ray cell

(36. Helical thickenings in vessel elements present)

(37. Helical thickenings throughout body of vessel element)

61. Fibers with simply to minutely bordered pits

66. Non-septate fibers present

69. Fibers thin- to thick-walled

79. Axial parenchyma vasicentric

85. Axial parenchyma in bands more than 3 cells wide

86. Axial parenchyma in narrow bands up to 3 cells

104. All ray cells procumbent

106. Body ray cells procumbent with one row of upright and/or square marginal cells

136. Prismatic crystals present

141. Prismatic crystals in non chambered axial parenchyma cells

142. Prismatic crystals in chambered axial parenchyma cell

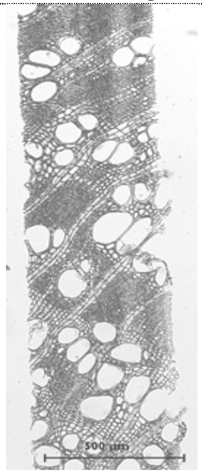
500 µm

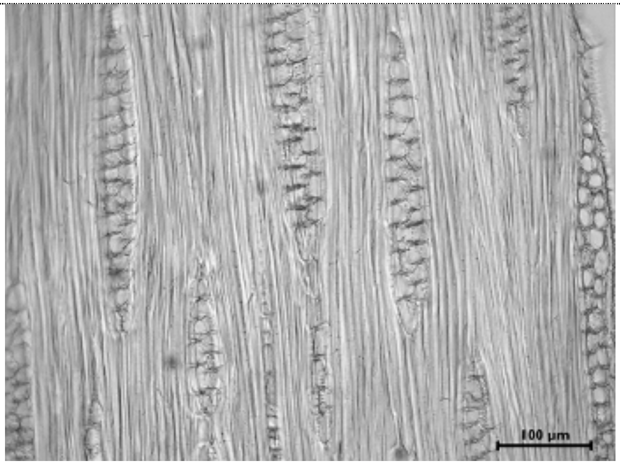
100 µm

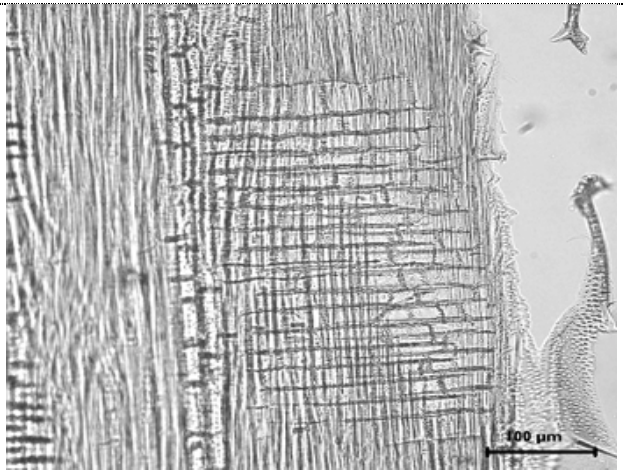
100 µm

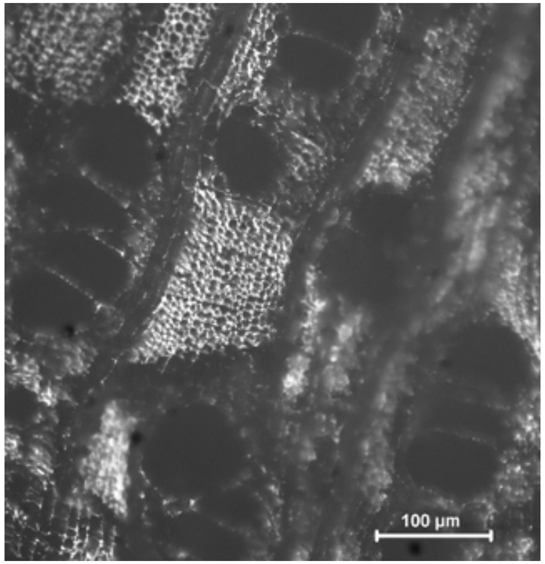
100 µm

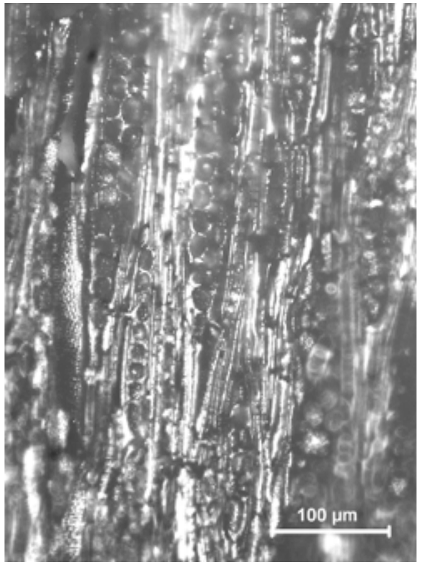
100 µm

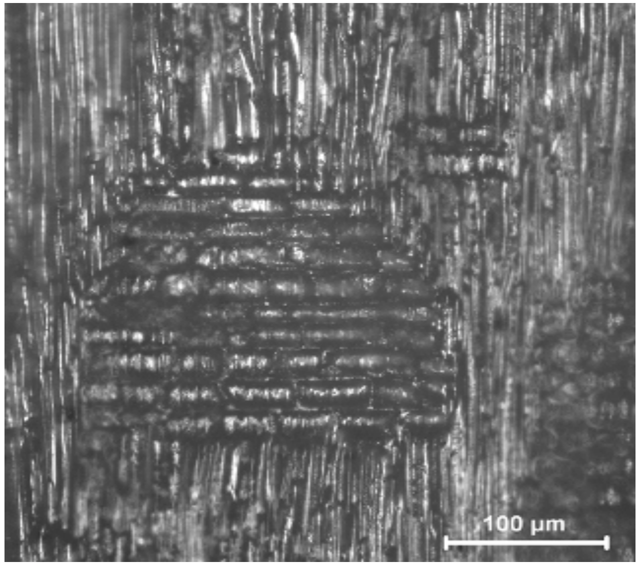
100 µm

***Ficus benghalensis* L.**

2. Growth ring boundaries indistinct or absent

5. Wood diffuse-porous

13. Simple perforation plates

22. Intervessel pits alternate

23. Shape of alternate pits polygonal

45. Vessels of two distinct diameter class, wood not ring-porous

56. Tyloses common

61. Fibers with simple to minutely bordered pits

62. Fibers with distinctively border pits

66. Non septate fibers present

69. Fibers thin- to thick-walled

85. Axial parenchyma in bands more than 3 cells wide

91. Two cells per parenchyma strands

92. Four (3-4) cells per parenchyma strand

93. Eight (5-8) cells per parenchyma strand

103. Rays of two distinct sizes

106. Body ray cells procumbent with one row of upright and/or square marginal cells

107. Body ray cells procumbent with mostly 2-4 rows of upright and / or square marginal cells

136. Prismatic crystals present

(137. Prismatic crystals in upright and/or square cells)

141. Prismatic crystals in non chambered axial parenchyma cells

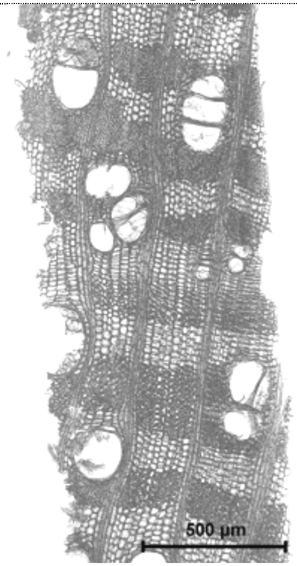
500 µm

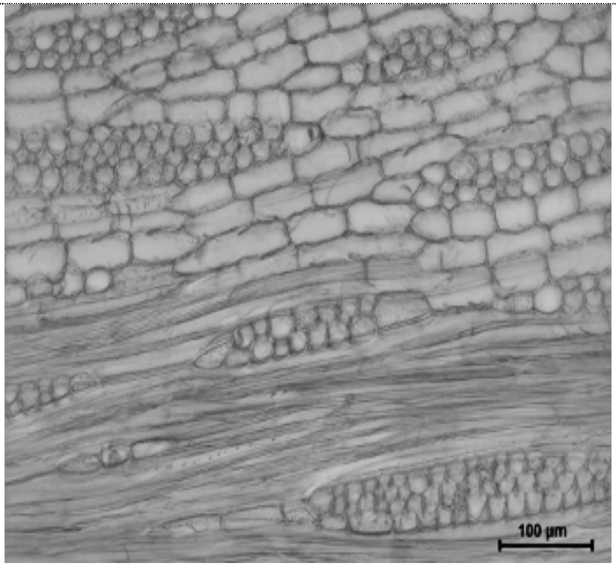
100 µm

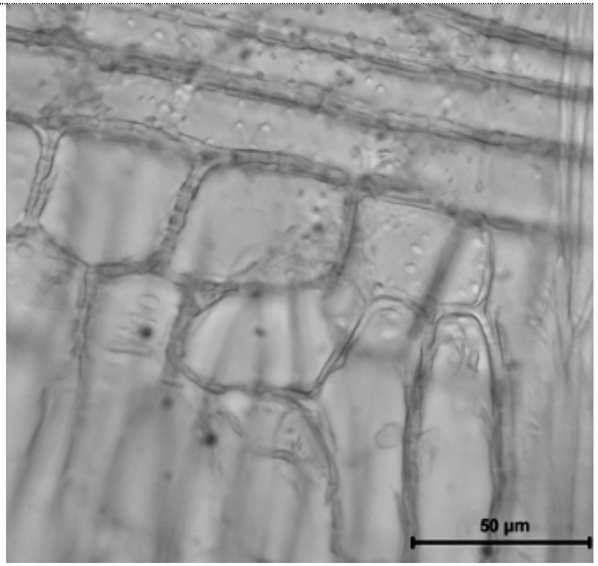
50 µm

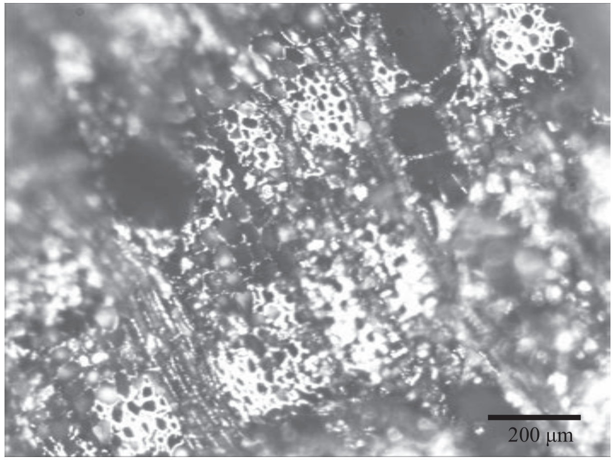
200 µm

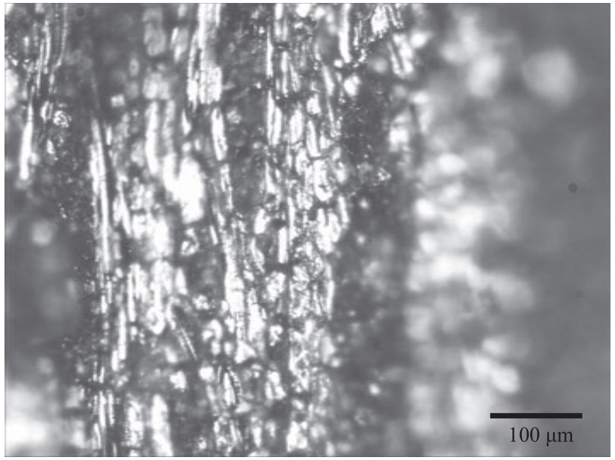
100 µm

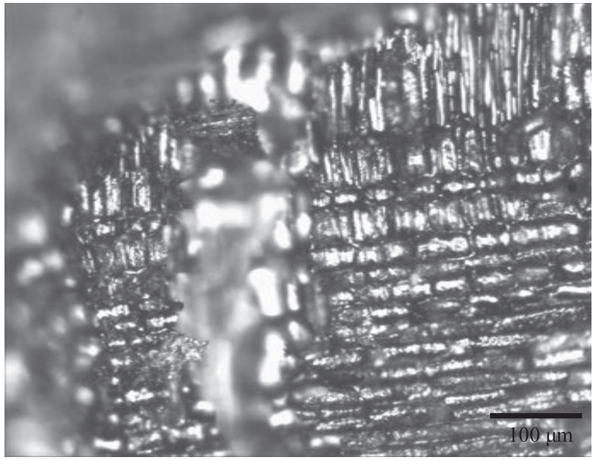
100 µm

***Ficus religiosa* L.**

2. Growth ring boundaries indistinct or absent

5. Wood diffuse-porous

7. Vessels in diagonal and/or radial pattern

10. Vessels in radial multiples of 4 or more common

13. Simple perforation plates

22. Intervessel pits alternate

26. Medium – 7-10 µm

32. Vessel-ray pits with much reduced borders to apparently simple: pits horizontal (scalariform, gash-like) to vertical (palisade)

65. Septate fibers present

66. Non septate fibers present

69. Fibers thin- to thick-walled

85. Axial parenchyma in bands more than 3 cells wide

98. Larger rays commonly 4 to 10-seriate (4-5 seriate in the sample)

106. Body ray cells procumbent with one row of upright and/or square marginal cells

107. Body ray cells procumbent with 2-4 rows of upright and/or square marginal cells

S2: Wood and charcoal catalogue

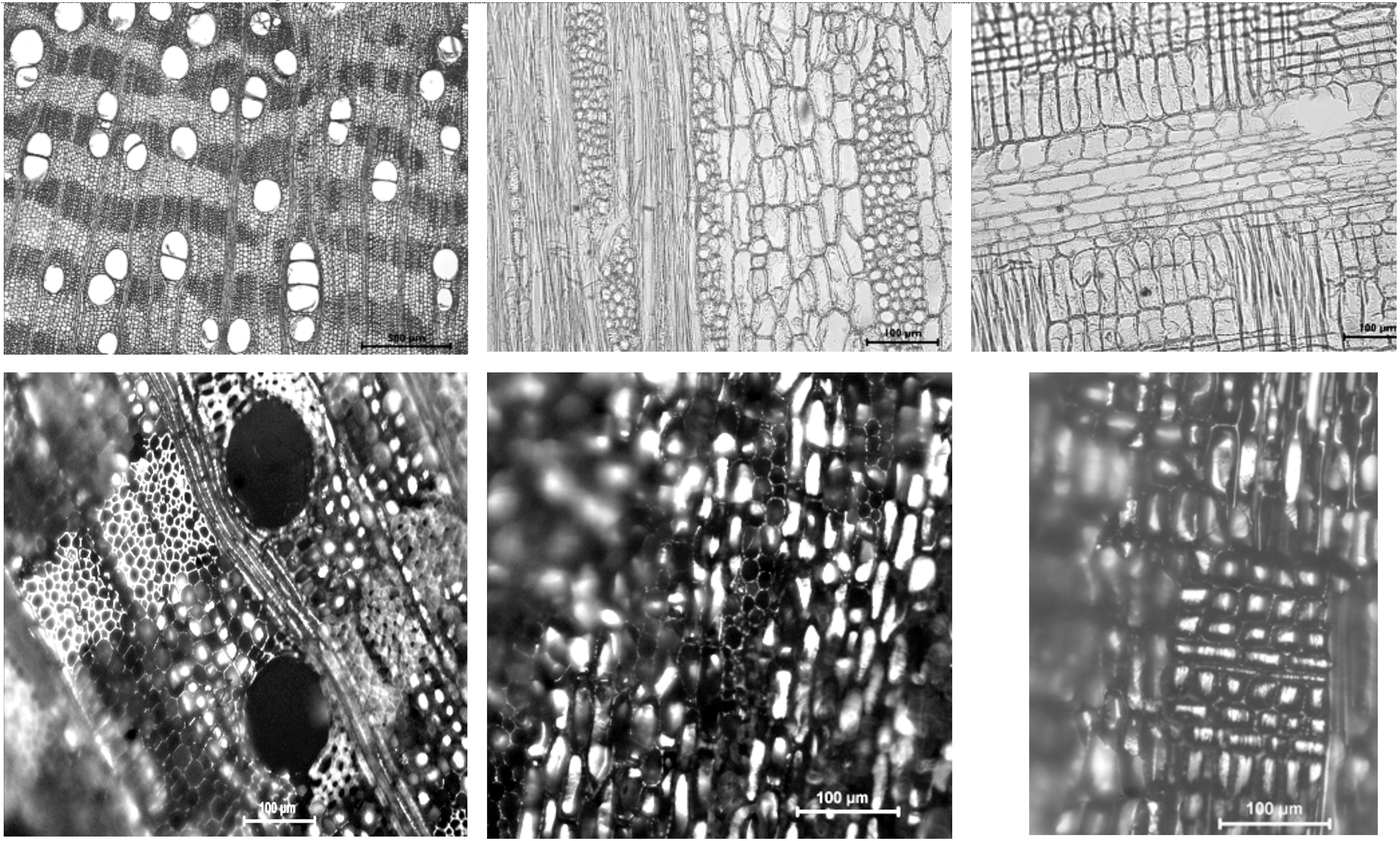

Ziziphus mauritiana **Lam.**

2. Growth ring boundaries indistinct or absent

5. Wood diffuse-porous

7. Vessels in diagonal and/or radial pattern

10. Vessels in radial multiples of 4 or more common

13. Simple perforation plates

22. Intervessel pits alternate

66. Non septate fibers present

68. Fibers very thin-walled

69. Fibers thin- to thick-walled

79. Axial parenchyma vasicentric

80. Axial parenchyma aliform

82. Axial parenchyma winged-aliform

96. Rays exclusively uniseriate

105. All ray cell upright and/or square

136. Prismatic crystals present

137. Prismatic crystals in upright and/or square ray cells

138. Prismatic crystals in procumbent ray cells

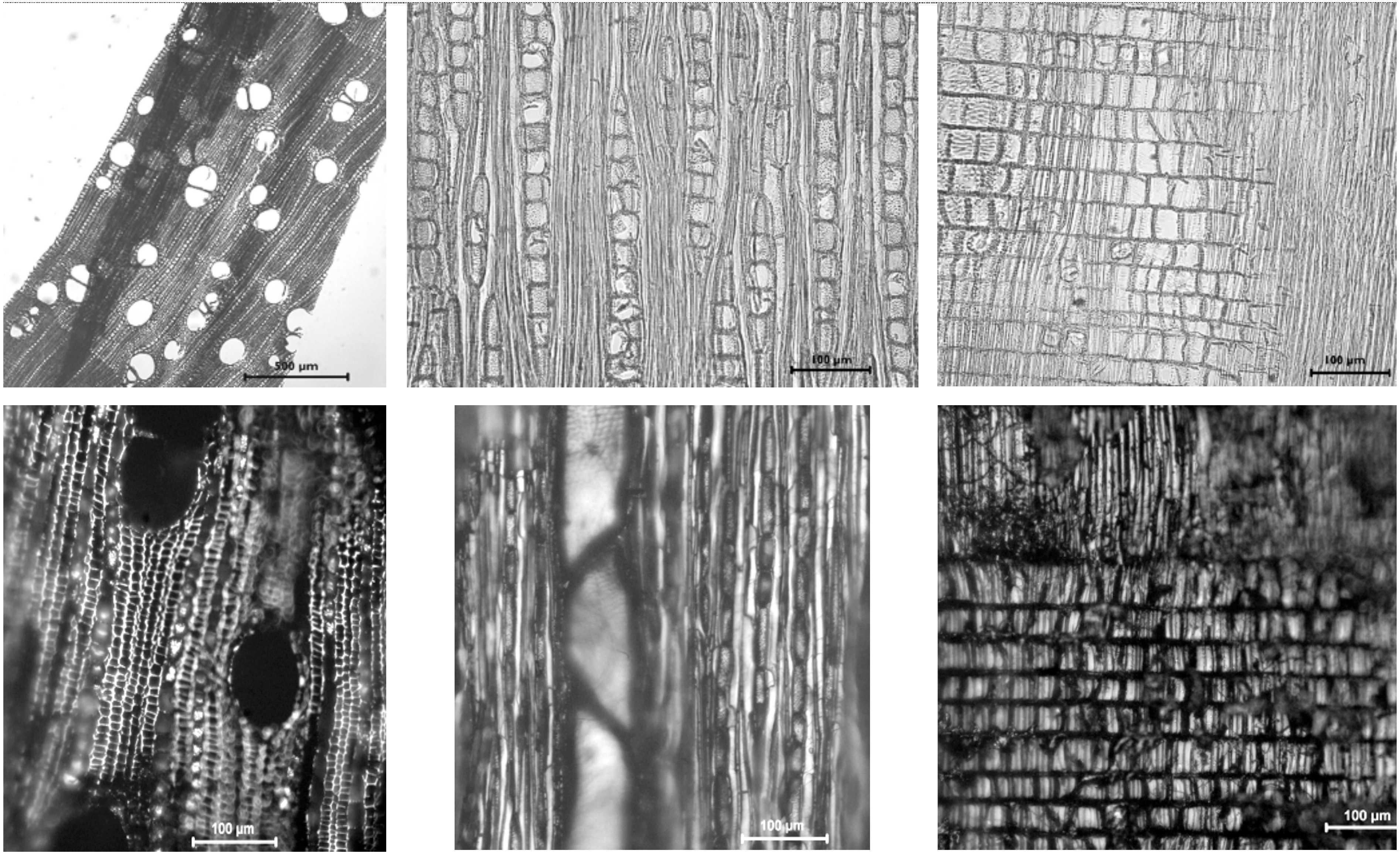
500 µm
100 µm
100 µm
100 µm
100 µm
100 µm

***Ziziphus nummularia* (Burn. f.) Wight & Arn**

2. Growth ring boundaries indistinct or absent

5. Wood diffuse-porous

7. Vessels in diagonal and/or radial pattern

10. Vessels in radial multiples of 4 or more common

13. Simple perforation plates

22. Intervessel pits alternate

29. Vestured pits

30. Vessel-ray pits with distinct borders; similar to intervessel pits in size and shape throughout the ray cell

66. Non septate fibers present

69. Fibers thin- to thick-walled

78. Axial parenchyma scanty paratracheal

83. Axial parenchyma confluent

84. Axial parenchyma unilateral paratracheal

86. Axial parenchyma in narrow bands or lines up to 3 cells wide

96. Rays exclusively uniseriate

102. Ray height > 1 mm

105. All ray cell upright and/or square

136. Prismatic crystals present

137. Prismatic crystals in upright and/or square cells

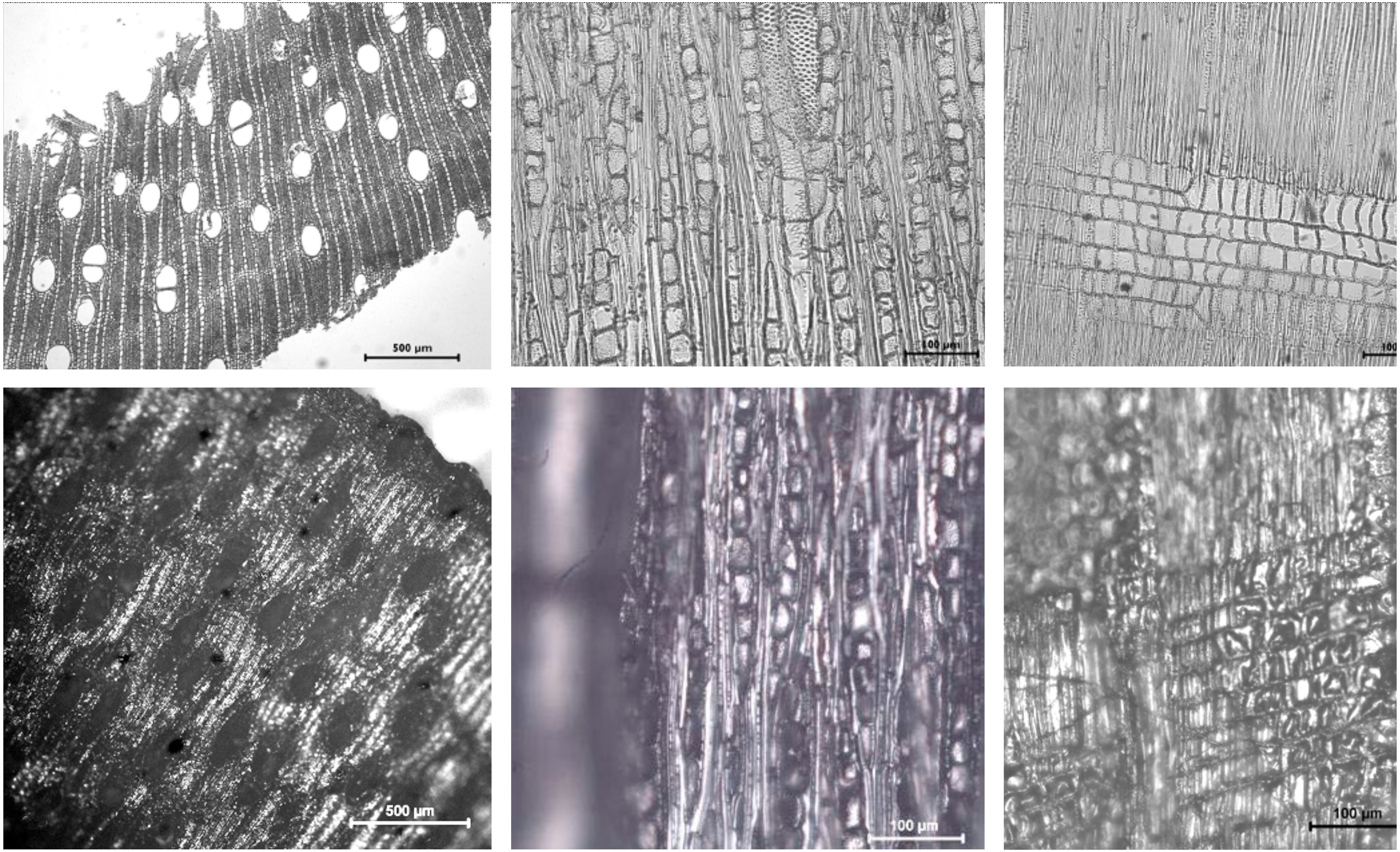
500 µm
100 µm
500 µm
100 µm
100 µm

***Salvadora oleoides* Decne**

2. Growth ring boundaries indistinct or absent

5. Wood diffuse-porous

10. Vessels in radial multiples of 4 or more common

11. Vessels clusters common

13. Simple perforation plates

22. Intervessel pits alternate

30. Vessel-ray pits with distinct borders; similar to intervessel pits in size and shape throughout the ray cell

45. Vessels of two distinct diameter class, wood not ring-porous

79. Axial parenchyma vasicentric

83. Axial parenchyma confluent

90. Fusiform parenchyma cells

91. Two cells per parenchyma strands

92. Four (3-4) cells per parenchyma strand

98. Larger rays commonly 4 to 10-seriate (2-4 seriate in the sample)

105. All ray cell upright and/or square

120. Axial parenchyma and/or vessel elements storied

136. Prismatic crystals present

137. Prismatic crystals in upright and/or square cells

S2: Wood and charcoal catalogue

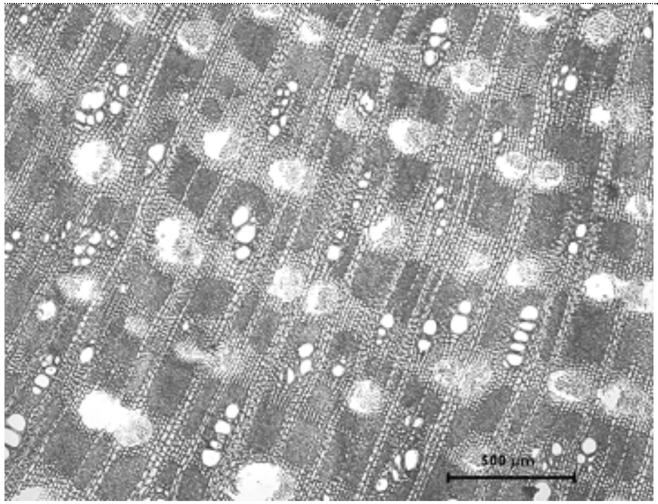

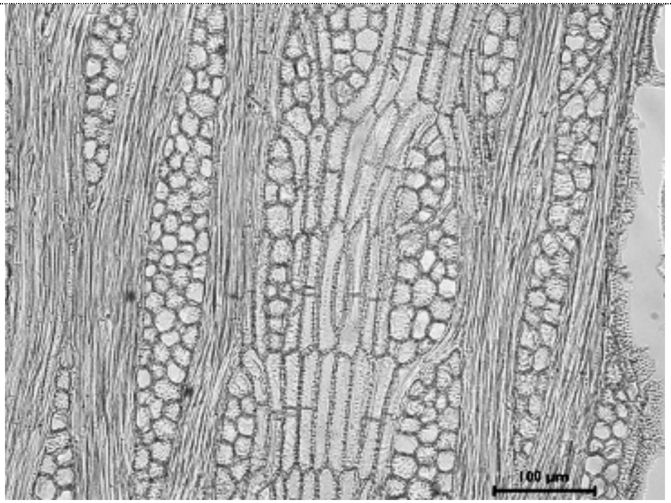

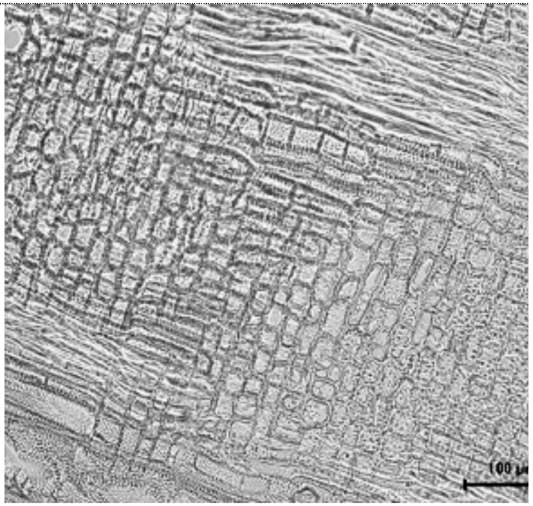

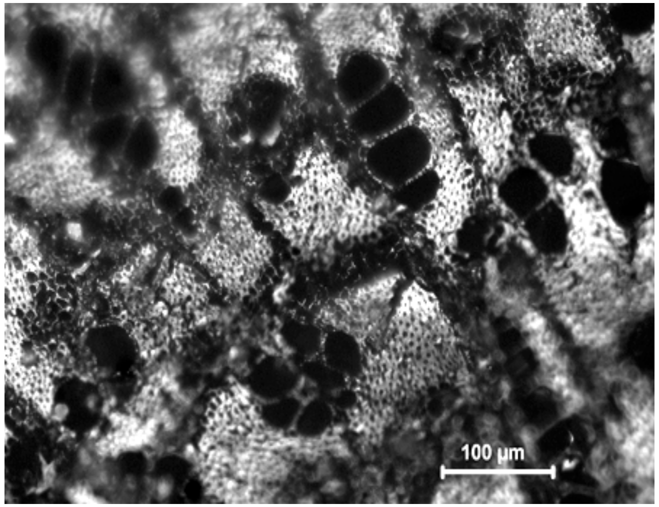

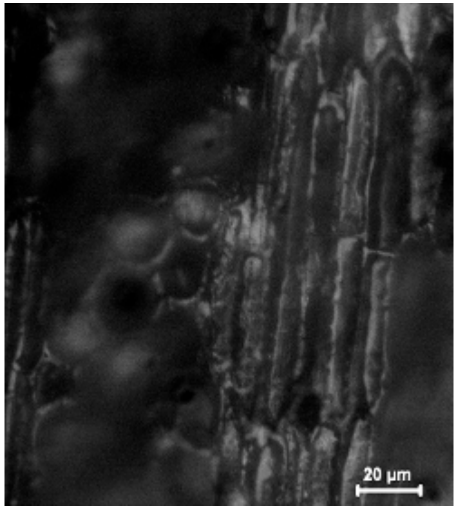

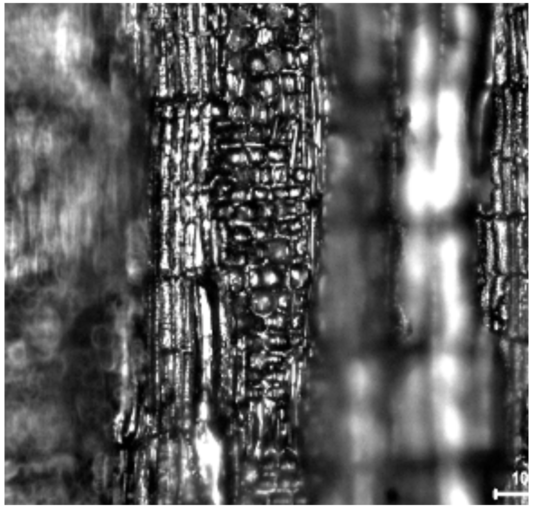

***Salvadora persica* L.**

1. Growth ring boundaries distinct
4. Wood semi-ring-porous
5. Wood diffuse-porous
11. Vessels clusters common
13. Simple perforation plates
22. Intervessel pits alternate
24. Minute - ≤ 4 µm
90. Fusiform parenchyma cells
91. Two cells per parenchyma strands
97. Ray width 1 to 3 cells
106. Body ray cells procumbent with one row of upright and/or square marginal cells
120. Axial parenchyma and vessel elements storied
136. Prismatic crystals present
137. Prismatic crystals in upright and/or square cells
138. Prismatic crystals in procumbent ray cells

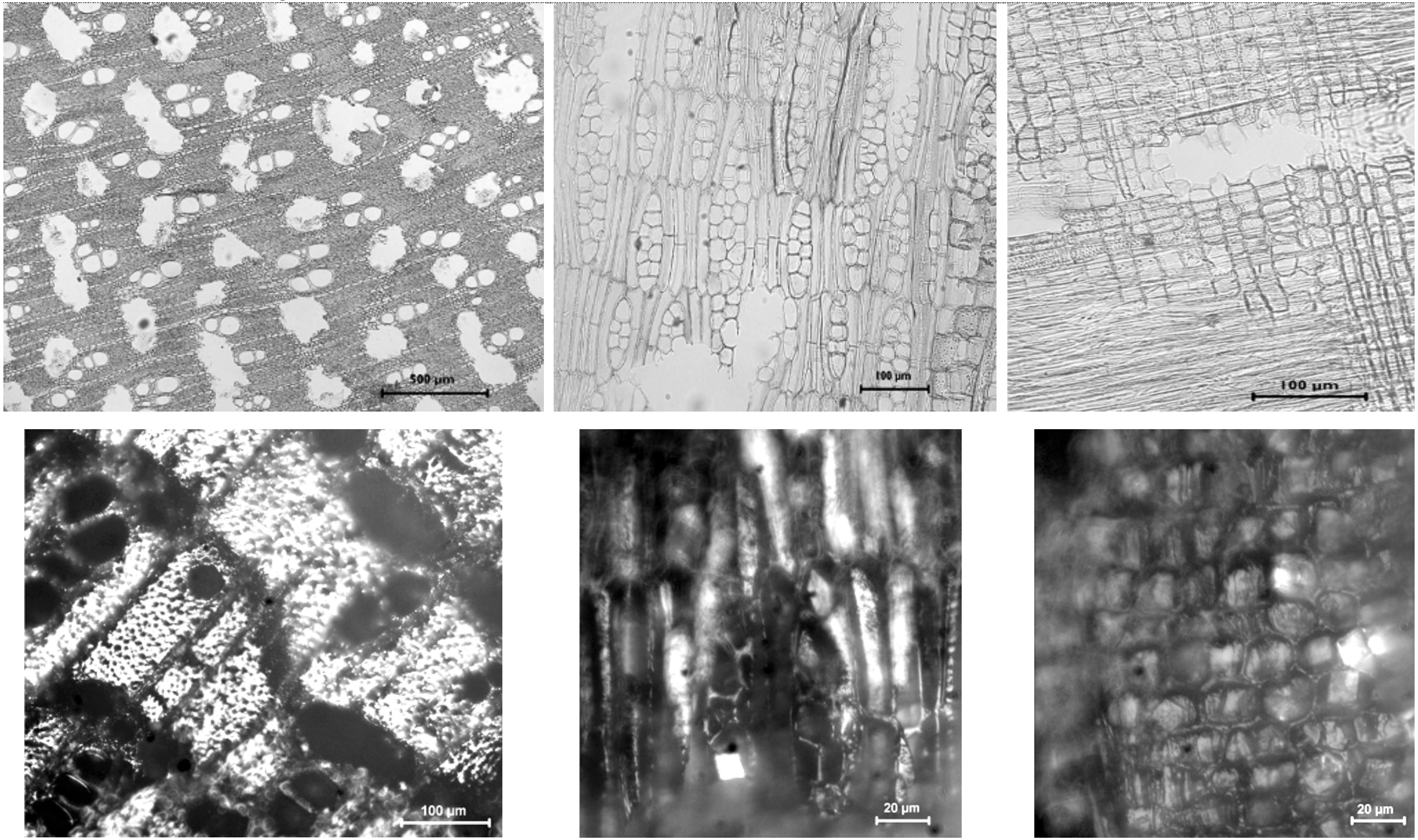
500 µm
100 µm
100 µm
100 µm
20 µm
20 µm

***Balanites aegyptiaca* (L.) Delile**

2. Growth ring boundaries indistinct or absent
5. Wood diffuse-porous
9. Vessels exclusively solitaire (90% or more)
13. Simple perforation plates
22. Intervessel pits alternate
45. Vessels of two distinct diameter class, wood not ring-porous
61. Fibers with simple to minutely bordered pits
62. Fibers with distinctively border pits (characteristic circular disposition)
70. Fibers very thick-walled
76. Axial parenchyma diffuse
98. Larger rays commonly 4 to 10-seriate
99. Larger rays commonly <10-seriate
102. Ray height > 1 mm
106. Body ray cells procumbent with one row of upright and/or square marginal cells
121. Fibers storied

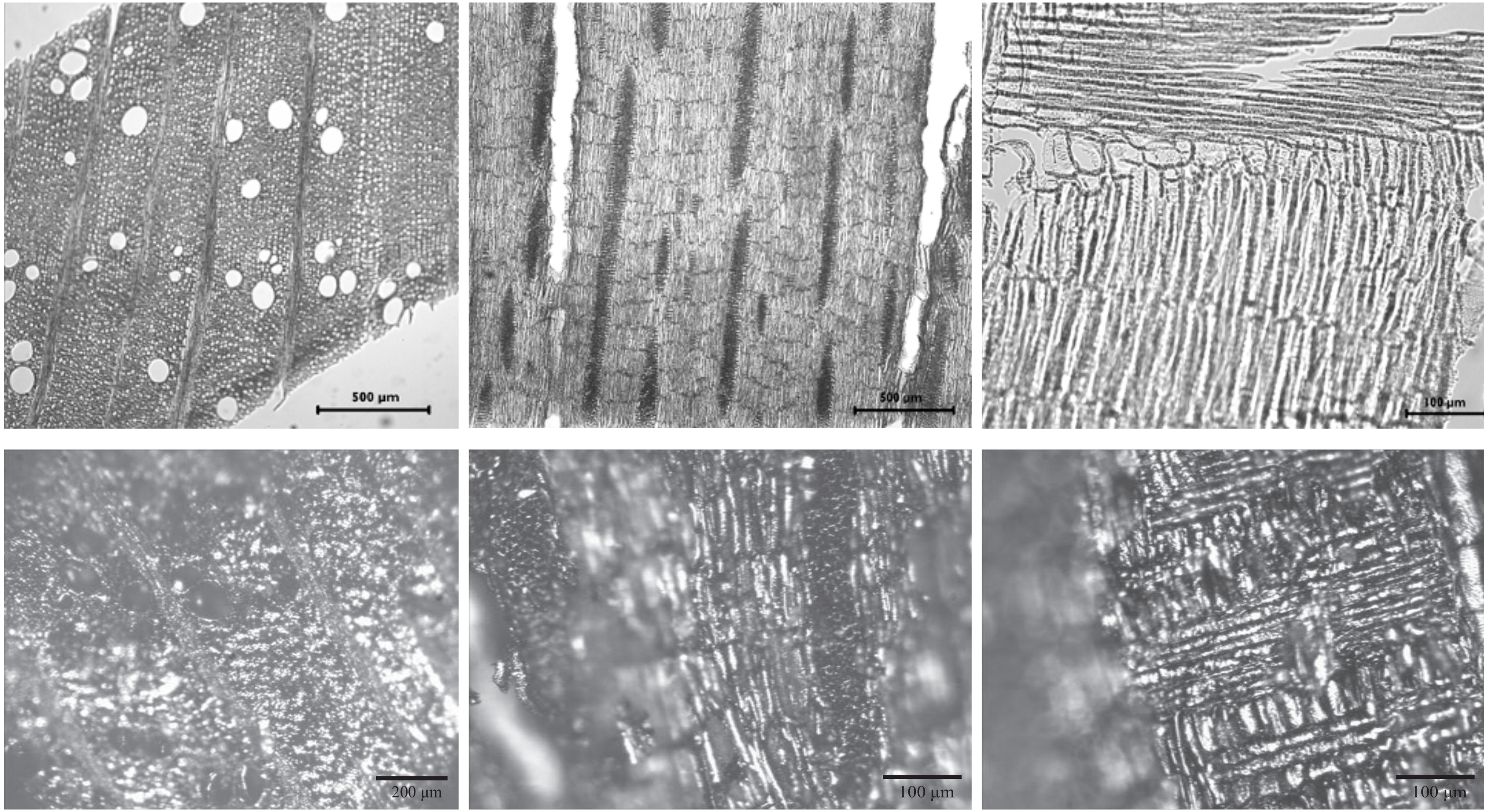
500 µm
500 µm
100 µm
200 µm
100 µm
100 µm
