# Supplementary material for: 'Not all that burns is wood'. A social perspective on fuel exploitation and use during the Indus urban period (2600-1900 BC)
Source: PLoS One. 2018 Mar 7;13(3):e0192364. doi: 10.1371/journal.pone.0192364 (PMC5841642; doi:10.1371/journal.pone.0192364)
Supplement: S1 Protocols — (DOC) [file pone.0192364.s010.doc]

# Protocols

In this section the protocols used for laboratory work are described. All the protocols are either in use at the George Pitt-Rivers Laboratory for Bioarchaeology, University of Cambridge, or were developed for this study modifying protocols used in the above or in other laboratories.

## Wood thin sections

**Equipment**

1. Microtome, sledge type.
2. Microtome blades, disposable type.
3. Small glass tubes (50mm x 24mm) with flat bottom and closures.
4. Tweezers.

**Chemicals**

1. Ethyl alcohol in 50, 70, 96 and 100% solution.
2. Sodium Hypochlorate 100%.
3. Acetic acid 10%.
4. Xylene

**Sample preparation**

The samples were cut in little blocks along the three planes of study: transverse, radial and tangential. It was tried to cut regular cubes as opposed to irregularly shaped ones as this facilitate the fixing in the microtome clamp. The best possible part of the wood was chosen, trying to avoid nodes, branches knots etc. If possible the cubes were cut as to have the sections exposed on the proper plane.

**Softening the wood**

The small cubes were placed in powder jars, filled with distilled water and placed in a drying cabinet at a temperature of *c.* 30 °C. The jars were capped in order to avoid evaporation and left in the drying cabinet for 5 weeks. Water was changed regularly (every 3 days) as to prevent the formation of fungi or mould. The addition of cold water to hot samples helped by creating a thermal shock that induced tenderness.

**Microtomy**

A Leica sledge microtome was used to section the wood samples. Disposable blades were used, fixed with an inclination of 30° and changed for every new specimen. The wood was kept moist by adding some distilled water and a mixture of ethyl alcohol and water (1:1) was spread on the blade in order to reduce curling. Around 20 sections for each plane were cut (when possible) and subsequently placed in a small glass vial filled with a solution of 50% ethanol.

**Section preparation**

The staining and de-hydration of the sections was done under a fume cupboard wearing lab-coat and gloves and using a set of beakers as to reduce to minimum the samples handling. The sets of passages followed are given in the table below (Table 1.1).

Table 1.1. Staining and de-hydration procedure applied to the wood thin sections (according to the protocol in use at the Tervuren xylarium, Brussels, Belgium)

| **Method** | **Time** |
| --- | --- |
| Remove sections form the water-alcohol solution |  |
| Bleach (increase the clarity of the wood) | 5 min.s (max.) |
| 4x rinses with distilled water | 5 min.s each (min.) |
| Acetic acid 10% vol. (purifies and soften the wood) | 5 min.s (max.) |
| 4x rinses with distilled water | 5 min.s each (min.) |
| Ethanol 50% vol. | 5 min.s (min.) |
| Safranine 1% (colours the wood) | 10 min.s (max.) |
| 2x 50% vol. Ethanol (start dehydration) | 5 min.s each (min.) |
| 70% vol. Ethanol | 5 min.s (min.) |
| 96% vol. Ethanol | 5 min.s (min.) |
| 96% vol. Ethanol | 30 min.s (min.) |
| 100% vol. Ethanol | Overnight |
| Xylene (to remove Ethanol) | 2 to 3 hours (min.) |

**Mounting**

A glass microscopy slide was labelled with the name of the wood species and its surface cleaned with ethyl alcohol (even if taken form a new packet) to remove any possible grease. One specimen of each section was put on the slide and covered with few drop of resin (Styrolite or Entellan). The sections were then covered with a cover slip and a little weight applied in order to push eventual air out. The slides were then left under a fume cupboard to dry for *c.* three weeks.

## Wood charring

**Equipment**

- - - 1. Aluminium foil
      2. Muffle furnace
      3. Crucibles

**Sample preparation**

Portions of wood were wrapped in aluminium foil so to form closed parcels and create reducing conditions. This prevented oxygen to participate in the reaction and reduce the wood into ashes. The species were recorded by an incision on the aluminium foil and by noting their position on the tray.

**Heating procedure**

Samples were introduced into a pre-heated muffle furnace at 400 ºC and left for 3 hours. The samples were than taken off the furnace and allowed to cool before unwrapping them. Heating was carried out in the Physical Geography Laboratory of the Department of Geography, University of Cambridge using a Lenton Furnace AF 11/6 and a Carbolite Furnace ELF 11/14B.

## Charcoal compression experiments

**Equipment**

Workbench with clamp

Wood saw with very fine teeth

Sample bags

Hounsfield Low Load Electric Screw Machine

**Sample preparation**

Small cubes of wood were hand-cut along the three planes observation Transverse, Tangential and Radial. For each species, 12 samples were obtained: two to be compressed on the transverse section and two to be compressed along the longitudinal direction at three different temperatures each. Samples were then wrapped in aluminium foil and put into a muffle furnace according to the protocol outlined for wood charring outlined in section 1.2. Temperatures were set at 200ºC, 300ºC and 400ºC and the samples were inserted into the furnace without pre-heating. The heating time was 6 hours in order to obtain changes even in the samples heated at the lowest temperature.

**Compressions**

Samples were put into transparent zip-lock laboratory bags and compressed using a Hounsfield low load electric screw machine with a 5kN static uniform load. The compression speed was fixed at 0.5 millimetres per minute and it was applied until material failure, i.e. no compression length was set. If failure did not occur the experiment was stopped when the compression reached 4 millimetres. Fragments were collected into the laboratory bags and stored for further analysis. Data were exported to and analysed with Excel.

## Phytoliths extraction from soil

The procedure followed, is the one described by Madella et al (1998) using Sodium Polytungstate as mean for separating phytoliths form sediments. The procedure was slightly modified in order to calculate the AIF (Acid-Insoluble Fraction) so that the results of the present study can be compared with others that use a different extraction technique.

**Equipment**

1. Precision balance

2. Drying cabinet

3. Fume cupboard

4. 100 ml beakers

5. Hot-plate

6. Centrifuge

7. 50 ml centrifuge tubes

8. Oven

9. Pasteur pipettes

10. Sealed vials

**Chemicals**

1. Hydrochloric acid (HCl - solution 7% vol.)

2. Sodium hexametaphosphate (NaPO_6_ - solution 5% weight)

3. Hydrogen peroxide (solution 33% vol.)

4. Sodium polytungstate (specific gravity 2.35)

5. Methylated spirit or Methanol

**Sample preparation**

Soil was dried in the dry cabinet until no loss of weight was measured then 4 to 5 grams of material were separated form the rest and put into a 100 millilitres beaker. A series of steps was then performed to eliminate the unwanted mineral fractions, i.e. carbonates and clay and the organic matter. The entire procedure is summarised in Table 1.2.

Table 1.2. Phytolith extraction from soil (slightly modified after Madella et al., 1998)

| **Procedure** | **Effect** |
| --- | --- |
| 1. Pour *c.* 25 ml of HCl (7-10%) into the beakers | Eliminate the carbonates |
| 2. Put the beakers, immersed in sand, on a hot plate |
| 3. Leave until the HCl is about 5 ml and the reaction has stopped |
| 4. Label and weight 50 ml centrifuge tubes |  |
| 5. Transfer the samples into the tubes and fill with distilled water | Wash the HCl away from samples |
| 6. Centrifuge for 3 minutes at 1500 rpm |
| 7. Discard the supernatant, fill with water |
| 8. Repeat steps 6 and 7 three times (4 centrifuges in total) |
| 9. Discard the supernatant |  |
| 10. Add up to 50 ml of NaPO6 | Deflocculate clays |
| 11. Shake well and leave overnight or for a minimum of 8 hours |
| 12. Repeat steps 6, 7, 8 and 9 | Wash the NaP06 away from samples |
| 13. Add up to 25 ml of H2O2 | Digest organic matter |
| 14. Shake well and re-open the tubes |
| 15. Put tubes into oven at 35ºC overnight or at least 8 hours |
| 16. Repeat steps 6, 7, 8 and 9 | Wash the H2O2 away from samples |

After these steps are completed, the samples are left to dry until no weight loss occurs. The content of the tubes represents the Acid Insoluble Fraction (AIF), which is weighted and then used to calculate phytoliths concentration in the samples.

**Phytoliths extraction**

Phytoliths were extracted from the AIF by adding up to 25 ml of sodium polytungstate (SPT) at a specific gravity of 2.35 g/ml. Phytoliths are lighter than the SPT therefore float at the top of the solution. They are then recovered with a Pasteur pipette and transferred in new 50 ml centrifuge tubes. Here the density is lowered again by adding distilled water so that the phytoliths are deposited at the bottom. Once the sodium is washed away by means of centrifuging, discarding the supernatant and adding water, the remaining residue is transferred into sealed glass vials and left to dry. The entire procedure is summarised in Table 1.3

Table 1.3. Extraction of phytoliths from AIF through gravity separation with sodium polytungstate.

| **Procedure** | **Effect** |
| --- | --- |
| 1. Prepare new 50 ml centrifuge tubes |  |
| 2. Add *c.*25 ml of SPT to the AIF | Phytoliths are suspended |
| 3. Centrifuge at 1500rpm per 3 minutes |
| 4. Recover phytoliths with a Pasteur pipette and transfer them in the new tube |  |
| 5. Repeat steps 3 and 4 |  |
| 6. Add water to the new tubes up to 50 ml | Pellet the phytoliths  at the bottom of the tube |
| 7. Centrifuge at 2000 rpm per 5 minutes |
| 8. Discard the supernatant and fill with water |
| 9. Repeat steps 6, 7 and 8 three times (four centrifuges in total) |
| 10. Label and weight 7 ml glass vials |  |
| 11. Transfer the phytoliths into sealed vials with methanol and leave to dry |  |

The residue in the vials is then weighted again to determine the total weight of silicates, a data that is also used to calculate phytoliths concentrations. After the residue is dried temporary or permanent slides can be mounted.

## Phytolith extraction from leaves

Two different methodologies were followed for phytolith extraction for modern plants. Most of the samples were treated directly on the field in order to reduce the volume that was transported back to the UK. These samples were ashed by putting them into a tin container and burned until reduced to ash. Part of the leaves was stored dry and treated in the lab according to the following procedure. Phytoliths from both extraction methods were compared in order to detect possible contamination in the samples treated on the field. The level of contamination was found insignificant therefore or the reference collection phytoliths extracted form the largest volume of leaves were used for analysis.

**Equipment**

1. Ultrasound bath
2. Furnace
3. Crucibles
4. Glass beakers
5. Fume cupboard
6. Centrifuge tubes (50 ml)
7. 7 ml glass vials

**Chemicals**

1. Sodium Hypochlorite (industrial or domestic bleach)
2. Methylated spirit or methanol

**Procedure**

Leaves were cut in small pieces, washed with distilled water and placed in beakers full of distilled water. The beakers were put into an ultrasonic bath for 15 minutes after which the water into the beaker was changed and the samples sonicated for other 15 minutes. After sonication the plants were rinsed again with distilled water and dried in the oven. These passages ensure that all particles deposited on the surface of the plants were removed before ashing in order to avoid contamination. The dry plants were weighted and then put into crucibles for ashing. Charring of plants was carried out in the Physical Geography Laboratory of the Department of Geography, University of Cambridge, using a Lenton Furnace AF 11/6 at 500ºC for 2 to 5 hours depending on their level of charring until they turned into white ash. The ash was then transferred into a 50 ml centrifuge tube and Sodium Hypochlorite was added and left overnight. The bleach was subsequently washed away by means of cycles of centrifuges with addition of distilled water. The last two centrifuges were performed by adding methanol instead of water and the residue left to dry and then mounted on slide. The entire procedure is summarised in Table 1.4.

Table 1.4. Laboratory procedure for the extraction of phytoliths form modern plant material.

| **Procedure** | **Effect** |
| --- | --- |
| 1. Cut plant parts in small pieces and wash with distilled water |  |
| 2. Place plants in a beaker filled with distilled water | Eliminate contaminants |
| 3. Place beakers in the ultrasound bath and sonicate for 15 minutes |
| 4. Change the water inside the beakers and sonicate for 15 minutes |
| 5. Rinse plant with distilled water and put in the oven to dry |
| 6. Weight dried plants |  |
| 7. Put plants into crucibles and cover with aluminium foil | Eliminate organic matter |
| 8. Furnace plants at 500ºC for 2 to 5 hours (until they turn to white ash) |
| 9. Label and weigh 50 ml centrifuge tubes |  |
| 10. Transfer ash into centrifuge tubes |  |
| 11. Add Sodium Hypochlorite and leave overnight or at least 8 hours | Clear dark material |
| 12. Centrifuge at 2000 rpm for 5 minutes, discard the supernatant and fill with water | Wash away bleach |
| 13. Repeat step 12 three times (4 centrifuge in total) |
| 14. Add methanol and centrifuge at 2000 rpm for 5 minutes | Dry ashes |
| 15. Discard the supernatant and repeat step 14 |
| 16. Label and weight 7 ml vials |  |
| 17. Transfer the residue in the vials and leave to dry |  |

## Phytolith extraction from dung cakes

Phytolith extraction from dung cakes followed a procedure similar to the one adopted for phytolith extraction from modern plant material. This is based on the removal of organic matter by burning the samples and then

**Equipment**

- - - 1. Furnace
      2. Tin foil trays
      3. Centrifuge tubes (50ml)
      4. Centrifuge
      5. Fume cupboard
      6. Oven
      7. 7 ml glass vials

**Chemicals**

Hydrochloric acid (HCl - solution 10% vol.)

Hydrogen Peroxide (H_2_O_2_ – solution 33% vol.)

**Sample preparation**

The dung cakes were weighted, broken into small pieces and placed into tin foil trays previously labelled. They were then burned at 400ºC for 24 and at 480ºC for additional 24 hours in a Lenton Furnace 11/6 in the Physical Geography Laboratory of the Department of Geography, University of Cambridge. The long exposure to high temperature was necessary in order to obtain pure white ash.

**Phytolith extraction**

The ash was transferred into previously labelled and weighted 50 ml centrifuge tubes and *c.* 10 ml of Hydrochloric acid were added and left for 15 minutes until the reaction stopped. The acid was then washed away with a series of centrifuges by discarding the supernatant and filling each time the tube with deionised water. Following the washes, *c.* 10 ml of Hydrogen Peroxide were added to the residue and the tubes were placed in the oven at 35ºC overnight. In the morning, the H_2_O_2_ was washed away with cycles of centrifuges and deionised water and the residue transferred into 7 ml glass sealable vials and left to dry. The entire procedure is summarised in Table 1.5.

Table 1.5. Laboratory procedure for the extraction of phytoliths from dung cakes.

| **Procedure** | **Effect** |
| --- | --- |
| 1. Weigh and crush the dung cake in small pieces | Facilitate burning |
| 2. Place the pieces in a tin foil tray |  |
| 3. Burn samples in furnace at 400ºC for 24 hours | Allow formation of white ash |
| 4. Stir the samples and burn them at 480ºC for 24 hours |
| 5. Label and weight 50 ml centrifuge tubes |  |
| 6. Transfer ash in the centrifuge tubes |  |
| 7. Add *c.* 10 ml of HCl (10% vol.) | Dissolve carbonates |
| 8. Leave for 15 minutes or until the reaction has stopped |
| 9. Fill the tube with water and centrifuge at 1500 rpm per 3 minutes | Wash away HCl |
| 10. Discard the supernatant |
| 11. Repeat steps 9 and 10 three times (four centrifuges in total) |
| 12. Discard the supernatant and add *c.* 10 ml of H_2_O_2_ | Whiten eventual dark residue |
| 13. Place tubes in oven at 35ºC overnight |
| 14. Repeat steps 9, 10, 11 and 12 | Wash away H_2_O_2_ |
| 15. Label and weight 7 ml glass vials |  |
| 16. Transfer samples in vials and leave to dry |  |

## Permanent slide mounting of phytoliths

**Equipment**

- - - 1. Fume cupboard
      2. Small spatula
      3. Pointed metal object (e.g., dentist metal probes or explorers)
      4. Microscopy slides
      5. 24x40 mm slide covers
      6. Markers
      7. Precision scale (at least 4 digits after the zero)

**Chemicals**

Methanol or Acetone

Entellan New®

Microscope slides were cleaned with Methanol or Acetone and then labelled and weighted. Few milligrams of silicates (obtained at the end of the extraction procedures), usually around 0.0005 grams, were placed on the slides, which were then re-weighted in order to calculate the exact weight of silicates on slides. Few drops of Entellan New® were placed on the residue, which was mixed with a pointed metal object and covered with a slide cover slip. The slides were then left to dry for a week. The entire procedure is summarised in Table 1.6.

Table 1.6. Laboratory procedure for permanent slide mounting of phytoliths.

| **Procedure** | **Effect** |
| --- | --- |
| 1. Clean, label and weight microscope slides |  |
| 2. With a spatula mix the silicates obtained through extraction | Homogenise the sample |
| 3. Place few mg of silicates on the slide |  |
| 4. Weight the slide with silicates | Determine sample weight on the slide |
| 5. Place few drop of Entellan New® on the slide |  |
| 6. Mix the silicates in the resin | Spread silicates evenly |
| 7. Place a cover slip on top of the slide |  |
| 8. Leave to dry for a week |  |

## Spherulites extraction from soil and dung cakes

**Equipment**

- - - 1. Sieve with very fine mesh (47 μm)
      2. Compressed air
      3. Microscopy slide
      4. Cover slides
      5. Pasteur pipettes

**Chemicals**

Methyl Salicicate (for direct observation)

Entellan New® for permanent mounting

Microscopy slides were labelled and weighted. Some soil was rubbed on the slide through the 47 μm mesh and the slide weighted again to determine the quantity of soil under analysis. The sample was then mounted with a few drops of Methyl Salicicate or Entellan New® (according to the type of mounting chosen (temporary or permanent) and covered with a slide cover. The slides were then observed under a transmitted light microscope with cross-polarised light.

This protocol was suggested by Dr M. Canti, geoarchaeologist and soil micromorphologist at the English Heritage in Portsmouth. The entire procedure is summarised in Table 1.7.

Table 1.7. Laboratory procedure for extraction and mounting of spherulites form modern dung cakes and archaeological sediments.

| **Procedure** | **Effect** |
| --- | --- |
| 1. Label and weight microscope slides |  |
| 2. Rub sample on a 47 μm mesh directly on the slide | Pulverize sample |
| 3. Weight the slide again | Determine sample weight |
| 4. With a Pasteur pipette place a few drops of mounting medium on the sample |  |
| 5. Cover with slide cover and let to dry |  |

## Soil chemical analysis

Multi-element chemical analysis was performed by the ALS Laboratory group in Seville, Spain. The method chosen was ME-ICP41, an ICP-AES analysis of 35 mineral and metal elements with Aqua Regia Digestion. Before analysis, the samples were finely ground in an agate mortar in order to reduce the particle size under 75 μm.

**REFERENCES**

MADELLA, M., POWER-JONES, A. H. & JONES, M. K. (1998) A simple method of extarction of opal phytoliths from sediments using a non-toxic heavy liquid. *Journal of Archaeological Sciences,* 25**,** 801-803.

WHEELER, E. A., BAAS, P. & P.E., G. (Eds.) (2007) *IAWA List of Microscopic Features for Hardwood Identification by an IAWA Committee,* IAWA Bulletin 10(3), 219-332.
